# The *n*,π* States of Heteroaromatics: When are They the Lowest Excited States and in What Way Can They Be Aromatic or Antiaromatic?

**DOI:** 10.1021/acs.jpca.4c02580

**Published:** 2024-05-24

**Authors:** Nathalie Proos Vedin, Sílvia Escayola, Slavko Radenković, Miquel Solà, Henrik Ottosson

**Affiliations:** † Department of Chemistry—Ångström Laboratory, Uppsala University, 751 20 Uppsala, Sweden; ‡ Institut de Quìmica Computacional i Catàlisi and Departament de Química, Universitat de Girona, C/Maria Aurèlia Capmany, 69, 17003 Girona, Catalonia, Spain; § Donostia International Physics Center (DIPC), 20018 Donostia, Euskadi, Spain; ∥ Faculty of Science, University of Kragujevac, P.O. Box 60, 34000 Kragujevac, Serbia

## Abstract

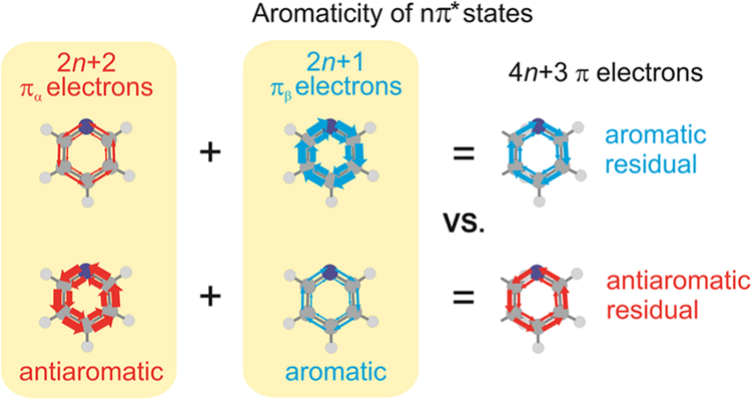

Heteroaromatic molecules are found in areas ranging from biochemistry to photovoltaics. We analyze the *n*,π* excited states of 6π-electron heteroaromatics with in-plane lone pairs (*n*_σ_, herein *n*) and use qualitative theory and quantum chemical computations, starting at Mandado’s 2*n* + 1 rule for aromaticity of separate spins. After excitation of an electron from *n* to π*, a (4*n* + 2)π-electron species has 2*n* + 2 π_α_-electrons and 2*n* + 1 π_β_-electrons (or *vice versa*) and becomes π_α_-antiaromatic and π_β_-aromatic. Yet, the antiaromatic π_α_- and aromatic π_β_-components seldom cancel, leading to residuals with aromatic or antiaromatic character. We explore vertically excited triplet *n*,π* states (^3^*n*,π*), which are most readily analyzed, but also singlet *n*,π* states (^1^*n*,π*), and explain which compounds have *n*,π* states with aromatic residuals as their lowest excited states (e.g., pyrazine and the phenyl anion). If the π_β_-electron population becomes more (less) uniformly distributed upon excitation, the system will have an (anti)aromatic residual. Among isomers, the one that has the most aromatic residual in ^3^*n*,π* is often of the lowest energy in this state. Five-membered ring heteroaromatics with one or two N, O, and/or S atoms never have *n*,π* states as their first excited states (T_1_ and S_1_), while this is nearly always the case for six-membered ring heteroaromatics with electropositive heteroatoms and/or highly symmetric (*D*_2*h*_) diheteroaromatics. For the complete compound set, there is a modest correlation between the (anti)aromatic character of the *n*,π* state and the energy gap between the lowest *n*,π* and π,π* states (*R*^2^ = 0.42), while it is stronger for monosubstituted pyrazines (*R*^2^ = 0.84).

## 1 Introduction

Approximately two-thirds of all compounds that were known at the end of the last century are fully or partially aromatic, and about half are heteroaromatic.^1^ The latter find applications in a wide array of areas including pharmaceutical chemistry, agrochemistry, and organic electronics.^1−8^ Thus, it is important to understand their electronic structures, and this applies to their singlet ground states (S_0_) as well as the first electronically excited states of singlet and triplet multiplicities (S_1_ and T_1_), where the latter states normally determine the photophysical and photochemical features. One characteristic is their extent of (anti)aromaticity,^1^ and in the lowest π,π* excited states, (anti)aromaticity is often given by Baird’s rule.^9−18^ This rule tells that annulenes with 4*n* π-electrons are aromatic in these states while those with 4*n* + 2 are antiaromatic. However, this form of excited state (anti)aromaticity is not valid for heteroaromatics with *n*,π* states as S_1_ and/or T_1_ states, e.g., pyrazine and *s*-triazine. Thus, how to assess and rationalize the potential aromatic or antiaromatic character of the *n*,π* states of heteroaromatics? Does the (anti)aromatic character influence which state is the lowest in energy, the *n*,π* or the π,π* state?

**Figure 1:**
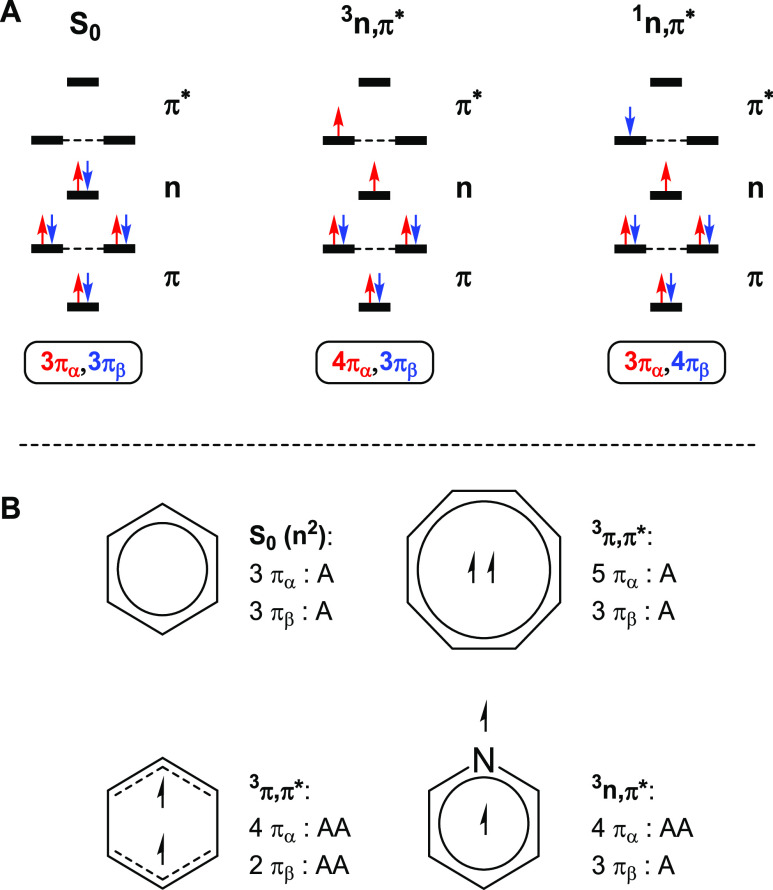
(A) Orbital occupancies in the S_0_ state (*n*^2^) and the triplet and singlet *n*,π* states, with α-electrons in red and β-electrons in blue. (B) Illustrations of Mandado’s rule for (anti)aromaticity of separate spins with aromaticity (A) and antiaromaticity (AA) components in the S_0_ and the triplet π,π* (T_1_) states of benzene, the triplet π,π* (T_1_) state of cyclooctatetraene, and the triplet *n*,π* state of pyridine.

A heteroaromatic molecule with six π-electrons in S_0_ (three of each spin) will in its *n*,π* state, where *n* is an in-plane orbital, have four π-electrons of one spin (α spin) and three of the other (β spin), and this applies to both the singlet and triplet *n*,π* state (Figure 1A). To understand their aromatic, nonaromatic, or antiaromatic characters, we utilize Mandado’s 2*n* + 1 rule for aromaticity of separate spins.^19^ With this rule, Hückel’s 4*n* + 2 rule for closed-shell singlet state aromaticity is fractioned into a 2*n* + 1 π_α_-electron part and a 2*n* + 1 π_β_-electron part (Figure 1B), while Baird’s 4*n* rule for the lowest π,π* triplet state of [4*n*]annulenes is fractioned into a 2*n* + 1 π_α_-electron and a 2*n* – 1 π_β_-electron part, all four numbers corresponding to aromaticity for separate spins. Conversely, the lowest π,π* triplet state of a species with 4*n* + 2 π-electrons is antiaromatic, having 2*n* + 2 π_α_- and 2*n* π_β_-electrons. A similar rule, derived by Valiev et al., tells that molecules are aromatic (antiaromatic) when having an odd (even) number of doubly and singly occupied π-orbitals,^20^ suggesting that Mandado’s rule should apply also to singlet excited states with the same electron configuration as the π,π* triplet state.

We have recently revealed the difficulty in proper assessment of excited state aromaticity through molecules which instead of Baird-aromatic are Hückel-aromatic or Hückel-Baird hybrid-aromatic in their lowest excited states.^21−23^ The situation also becomes more complex in heteroaromatics with in-plane lone pairs (*n*_σ_, herein labeled *n*). The *n*,π* state of such a (4*n* + 2)π-electron molecule has 2*n* + 2 π_α_- and 2*n* + 1 π_β_-electrons (or *vice versa*), and would at first glance be nonaromatic if the aromatic π_β_-component exactly cancels the antiaromatic π_α_-component. Yet, can the two parts combined instead lead to aromatic or antiaromatic residuals?

Although the (anti)aromatic characters of various heteroaromatics in their lowest excited states have been analyzed earlier through computations,^24−27^ the (anti)aromaticity of the *n*,π* states has to the best of our knowledge not been addressed, neither through qualitative theory nor quantitative computations. Such information can be important to rationalize characteristics of *n*,π* states. Compounds with *n*,π* states with aromatic residuals may have these as their T_1_ and S_1_ states, with potentially lower excitation energies and higher photochemical stabilities than (isomeric) compounds with *n*,π* states having non- or antiaromatic residuals. However, a number of additional factors also impact, as can be expected because the relative orbital energies and positions of the heteroatoms in the rings should also play a role.

Here, two recent computational findings are noteworthy. In the first, Foroutan-Nejad points out the lack of correlation between paratropicity and aromatic stabilization energy in monocyclic π-conjugated hydrocarbon radicals,^28^ and in the second, Zhu et al. uses the concept of adaptive aromaticity in 16- as well as 18-electron metallaaromatic compounds with lowest excited states of π,σ* or σ,π* character.^29−33^ Both monocyclic π-conjugated hydrocarbon radicals and the metallaaromatics in the states under consideration differ by one electron in the π_α_- versus π_β_-electron counts. Can our analysis approach be extended to these and other species with odd total numbers of π_α_- and π_β_-electrons? If so, one may utilize the analysis to species which are not traditionally regarded as heteroaromatics, e.g., carbenes of relevance for astrochemistry or as ligands in complexes for catalysts, or molecules with charge transfer states composed of cyclic radical cationic and anionic moieties.

**Figure 2:**
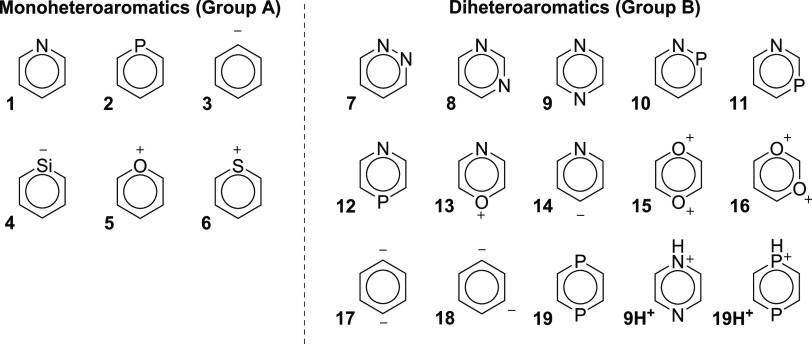
6-Membered ring (6-MR) heteroaromatics investigated herein. For a selection of 5-membered ring (5-MR) heteroaromatics and an analysis of their frontier orbital energies, see the SI (Section S3.7).

The extent of aromaticity in the S_0_ state of many heteroaromatics has already been reported.^34,35^ The compounds now investigated in their *n*,π* states (Figure 2) are grouped so as to allow us to explore the effects of heteroatom electronegativity, the number of heteroatoms, and their relative positions. We focus on six-membered ring (6-MR) heterocycles as we find that 5-MR heteroaromatics (e.g., furan and imidazole) always have *n*,π* states of high energies placed well above their first π,π* states, making them difficult to observe experimentally.^36^ Hence, the latter species are only discussed in the Supporting Information (SI, Section S3.7). Among the 6-MR species, the phenyl anion and dianions (**3**, **17**, and **18**) are not heterocycles, but we consider the anionic sp^2^ hybridized C atoms with in-plane lone pairs as heteroatoms. Thus, even though most of the compounds are common heterocycles, we included species that allow us to identify general trends. We focus on heteroaromatics with *n*,π* states among the lowest few excited states, whereby they are photochemically relevant. Yet, the *n*,π* states are not necessarily T_1_ and S_1_ for all compounds as that allows us to reveal trends. As far as we know, no investigation into which heteroaromatics have *n*,π* states as their lowest excited states and which ones have π,π* states has earlier been reported. Thus, the main objectives of our study are to resolve (if possible) which heteroaromatics have *n*,π* states as their lowest excited states and which ones have π,π* states as these states, and to what extent (anti)aromaticity plays a role for this.

As will be shown, by considering the residuals between the two spin components of (anti)aromaticity, we rationalize features of the heteroaromatics in their *n*,π* states, e.g., when these states are the lowest excited states. Hence, we provide a theoretical framework for the (anti)aromatic character of *n*,π* states that can be generalized to states with even π_α_- and odd π_β_-electron counts (or vice versa). We pinpoint why excited state (anti)aromaticity investigations require both quantitative computations and qualitative theory if one is to arrive at conclusions that are correct and comprehensive. Such fundamental knowledge should allow for more insightful design of molecules with targeted properties and applications, especially if linked to machine-learning approaches.

## 2 Qualitative Theory

Before discussing computational results, we describe the qualitative theoretical framework, and we focus on the triplet *n*,π* state as this is more readily analyzed computationally than the singlet *n*,π* state (*vide infra*). We foremost explore vertical triplet excitations because in the vertical *n*,π* state the π_β_-component should, viewed simplistically (Figure 1A), remain as aromatic as in S_0_, while the π_α_-component with four π_α_-electrons should be antiaromatic. Factors that may perturb this description are (i) a difference in electrostatics within the π-orbital frameworks of the S_0_ and *n*,π* states as there will be an increased Coulomb repulsion in the π-system due to the additional π-electron in the *n*,π* state, and (ii) a difference in the exchange interaction between the two states resulting from the change in the number of π_α_- and π_β_-electrons. A residual that tends toward aromaticity of a vertically excited n,π* state will result if there is a higher degree of aromaticity in the π_β_-component compared to the S_0_ state and/or a low degree of antiaromaticity in the π_α_-component. Similarly, a residual that tends toward antiaromaticity can stem from a lower aromaticity in the π_β_-component than in S_0_ and/or a high degree of antiaromaticity in the π_α_-component.

**Figure 3:**
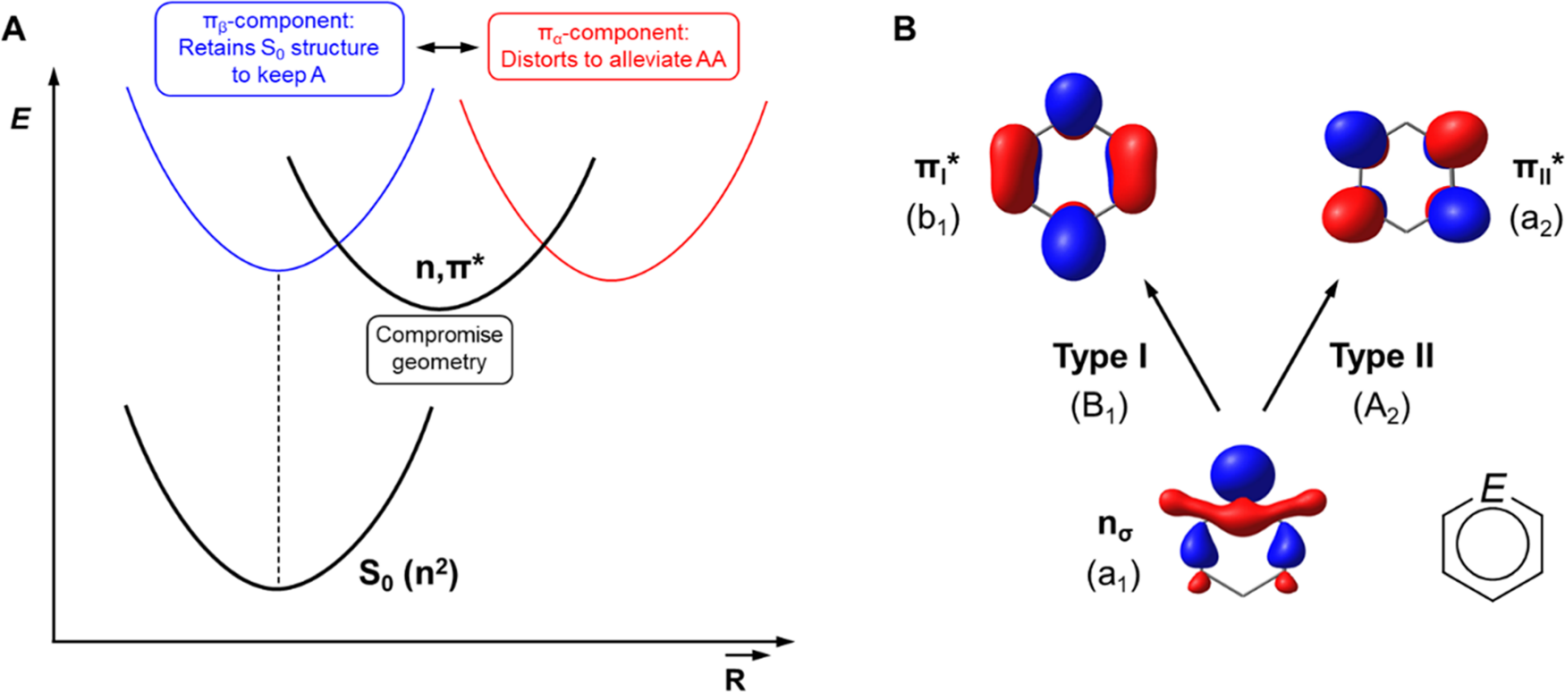
(A) Illustration of the tug-of-war between the aromatic (A) π_β_-component (blue) and the antiaromatic (AA) π_α_-component (red) in influencing the structure of the *n*,π* state. (B) The two general types of *n*,π* excitations for a heteroaromatic molecule with *C*_2*v*_ symmetry that can be the lowest *n*,π* state, as well as the orbital and state symmetries.

Next, when the molecule relaxes from the vertically excited *n*,π* state one can postulate that there will be a tug-of-war between the aromatic π_β_- and the antiaromatic π_α_-components. The first seeks to retain the (planar) S_0_ state geometry and the second strives to alleviate its antiaromaticity through geometric distortions (Figure 3A), i.e., to obtain a more bond length alternated and/or puckered structure. Thus, if the residual between the π_α_- and π_β_-(anti)aromaticity components corresponds to some aromatic character, we postulate that the molecule will be more prone to retain a planar and/or bond length equalized structure in the *n*,π* state, while it will pucker and/or become more bond length alternated if it has a residual with antiaromatic character. Yet, there can be factors that counteract these features, e.g., a preference of a particular heteroatom to have a more acute bond angle than the planar structure allows for. Hence, by jointly regarding the aromatic-antiaromatic character of the vertically excited *n*,π* state, we probe the hypothesis that molecules with *n*,π* states with aromatic residuals between the π_α_- and π_β_-components distort less while those with antiaromatic residuals distort more.

There are also various types of *n*,π* states with different state symmetries as there are two π* orbitals (the b_1_ and a_2_ orbitals, Figure 3B), which are degenerate in benzene. As the b_1_ π* orbital has a lobe at the *E* atom while the a_2_ π* orbital has a node there, it is apparent that the orbital energy gap and the energies of the two *n*,π* states (*E*(*n*,π*)) are affected differently by the electronegativity of atom *E*. This indicates that the *n*,π* excitation energies of heteroaromatic compounds will be affected by several features, where the extent of *n*,π* state (anti)aromaticity is only one, others being the S_0_ state (anti)aromaticity and differences in electrostatic repulsion, exchange interaction, and strain within the molecule when going from S_0_ to the excited state. Thus, a correlation between the (anti)aromatic residual of an *n*,π* state and the excitation energy is unlikely. Still, correlations may be observed within closely related compounds, e.g., among isomers or substituted derivatives of a specific heteroaromatic.

The situation becomes more complex with two (or further) heteroatoms *E* and *E*′ with in-plane lone-pair electrons because the excitation can be out of either the in-phase or out-of-phase combination of the local *n*(*E*) and *n*(*E*′) orbitals. Throughout we discuss the lowest *n*,π* state, yet in a few cases we explore also the second lowest to establish an unambiguous comparison between analogous *n*,π* states of a subset of the mono- and diheteroaromatics. Moreover, the lowest ^3^*n*,π* state is not necessarily the T_1_ state. Here, we found that 6-MR heteroaromatics often have T_1_ states of *n*,π* or π,π* character, while the T_1_ state of 5-MR heteroaromatics (as mentioned above) mainly are of π,π* character as the in-plane *n* orbitals are of lower and the π* orbitals of higher energy than in the 6-MR heteroaromatics (see Section S3.7, SI).

## 3 Results and Discussion

The mono- and diheteroaromatics of Figure 2 were analyzed separately. We primarily explored their lowest triplet *n*,π* state (^3^*n*,π*) as this allows us to use a larger portfolio of aromaticity descriptors. Yet, we also calculated several compounds in their singlet *n*,π* states (^1^*n*,π*) to probe if trends are the same for singlet and triplet *n*,π* states. Emphasis is placed on electronic aromaticity indices as these are readily separated into α- and β-components, although the spin-separate magnetically induced current densities (MICDs) were also analyzed.^37,38^ For electronic indices, we computed (spin-separated) multicenter indices (MCI) and electron density of delocalized bonds (EDDB),^39−42^ with focus on results from MCI known to provide the most accurate predictions in a series of aromaticity tests.^35,43^ We further computed nucleus-independent chemical shifts (NICS)^44−46^ for the ^3^*n*,π* states, although not spin-separated (see the Computational Details in the SI). Thereby, the NICS values cannot explain the cause of the (anti)aromatic character of an *n*,π* state as the magnitudes of the individual spin components are unknown. For some ^3^*n*,π* states, we also analyzed geometry-based parameters and the relaxation energies when going from the vertically excited to the relaxed ^3^*n*,π* states: Do they reflect the drive to relieve antiaromaticity of the π_α_-component or the strive of the π_β_-component to retain aromaticity? The analysis of this tug-of-war (Figure 3A) is done jointly on the mono- and diheteroaromatic compounds in a final section on generalizations.

Computations for the ^3^*n*,π* states were mainly performed with the long-range corrected CAM-B3LYP functional^47^ in the unrestricted Kohn–Sham (KS) formalism, but calculations with the B3LYP and BLYP functionals,^48−51^ as well as CCSD, BD,^52^ and CASSCF, were performed for selected compounds (see Sections S1, S2.1, S3.1, and S3.6, SI). Recently, the long-range corrected CAM-B3LYP and ωB97X-D functionals, and the Minnesota functional M06-2X, among 10 different functionals were found to perform best for the lowest excited states of benzene, pyridine, and the diazines.^24^ For the ^1^*n*,π* states, we used time-dependent (TD) density functional theory (DFT).^53^

### 3.1 Assessment Criteria

For essentially all compounds, the σ-contributions to the MCI are negligible and do not modify the conclusions (Table S14, SI), and thus, we consider the σ- and π-components combined. We explored for which compounds the MCI_β_-components of the ^3^*n*,π* states are smaller (less aromatic) than in S_0_ (i.e., half of the total MCI value in S_0_), and for which compounds they are larger. The MCI_α_-component will decrease to a very low value as it becomes antiaromatic, yet its antiaromatic character cannot be assessed as easily as the aromatic character of the MCI_β_-component. The residual of the ^3^*n*,π* state is the combined MCI_α_- and MCI_β_-components (MCI(^3^*n*,π*)_tot_) minus half the S_0_ value (MCI(S_0_)_tot_); for a nonaromatic ^3^*n*,π* state, the MCI(^3^*n*,π*)_tot_ should be close to half the total value in S_0_, provided that the MCI_α_-component is nearly zero while the MCI_β_-component remains as in S_0_. Moreover, we label the ^3^*n*,π* state to lean toward aromaticity if the MCI(^3^*n*,π*)_tot_ is at least 10% higher than half the MCI(S_0_)_tot_, and toward antiaromaticity if 10% lower.

We consider a 6-MR molecule to be aromatic in S_0_ if its MCI value is at least half the MCI value of benzene in S_0_ (i.e., 0.0716/2 = 0.0358). However, the vertically excited lowest triplet π,π* state of benzene is multiconfigurational and is therefore not (easily) comparable to those of the lower-symmetry heterocycles. Instead, we used the MCI and EDDB values of the lowest ^3^π,π* state of pyridine, which is single-configurational and antiaromatic. This state is an antiaromatic reference with a total MCI value of −0.0005, and MCI_α_- and MCI_β_-components of 0.0023 and −0.0028, respectively.

Thus, in the (anti)aromaticity assessments of the *n*,π* states of a compound, we use two measures; (i) the ratio between the MCI(^3^*n*,π*)_tot_ and MCI(S_0_)_tot_ of a specific compound and its deviation from 50%, representing nonaromaticity, and (ii) a comparison of the S_0_ or *n*,π* state of a specific heteroaromatic with the aromaticity of benzene in S_0_ (aromatic reference) and the antiaromaticity of the triplet π,π* state of pyridine (antiaromatic reference).

### 3.2 Monoheteroaromatic 6-MRs (Group A)

As postulated above, the split in energy between the two π* orbitals, b_1_ and a_2_, increases with the electronegativity of heteroatom *E* as the b_1_ orbital is lowered more. The 1^3^B_1_ state is the lowest ^3^*n*,π* state for all 6-MR monoheteroaromatics, yet, the computed energy difference to the other ^3^*n*,π* state (1^3^A_2_) varies from 0.20 eV for **3** to 2.05 eV for **5** (and similarly for the ^1^*n*,π* states, except for **3** where 1^1^A_2_ is 0.42 eV below 1^1^B_1_). It is, however, notable that the lowest ^3^*n*,π* states of **5** and **6** have multiconfigurational character (see T_1_ diagnostics, Table S22, SI).^54−56^ Yet, despite this, the ratios between the MCI values in the ^3^*n*,π* and S_0_ states of **5** and **6** obtained from (U)CAM-B3LYP, (U)CCSD, and (U)BD calculations are quite similar (see Tables S1, S4, and S5 in the SI), clarifying that (U)CAM-B3LYP produces reliable estimates of the decrease in aromaticity when going from S_0_ to the ^3^*n*,π* states of these species (see further Section S3.6 in the SI).

**Figure 4:**
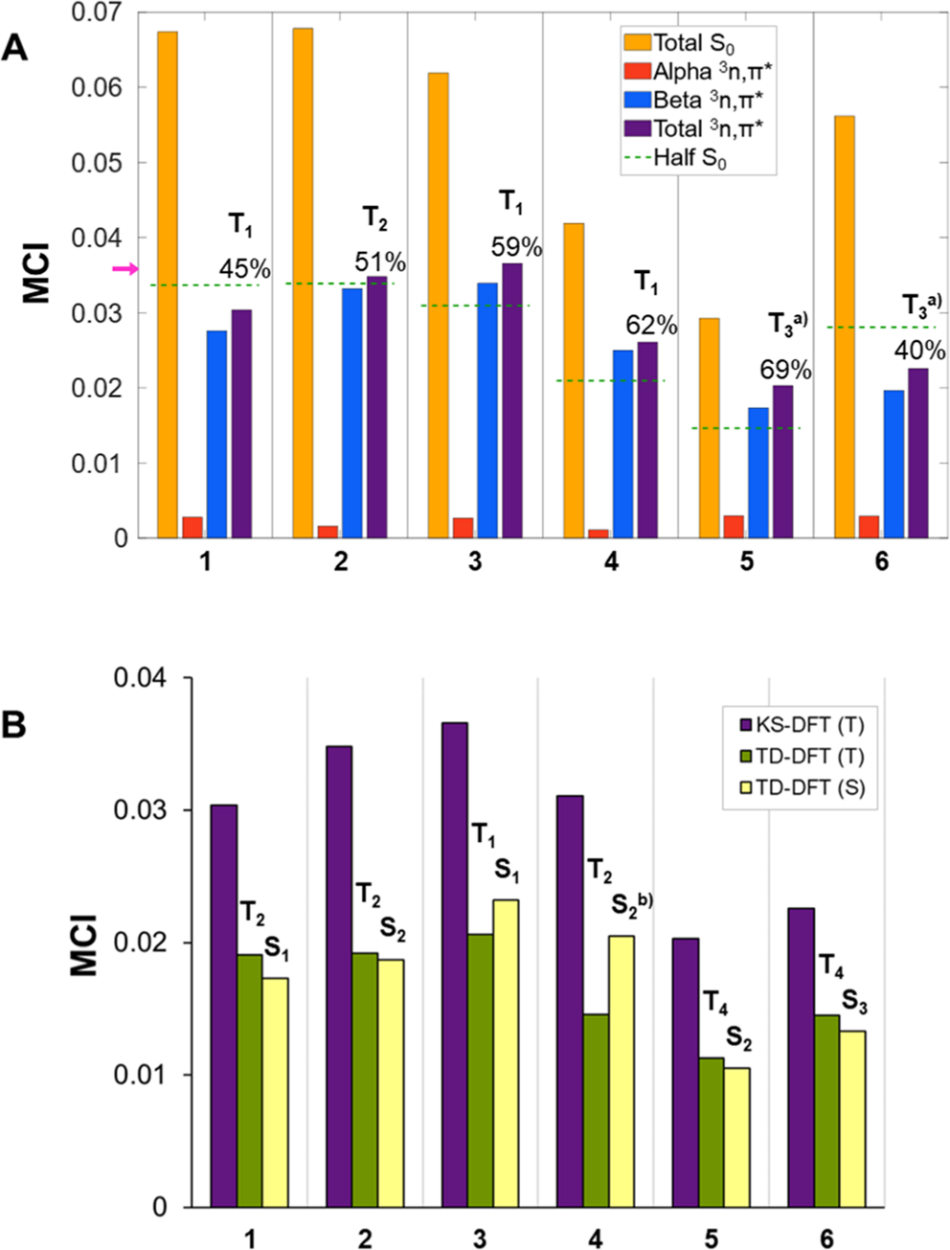
MCI results (in a.u.) of 6-MR monoheteroaromatics in (A) their *n*,π* triplet states (spin-separated MCI values) with KS-UDFT and (B) their singlet (S) and triplet (T) *n*,π* states at TD-DFT level, as well as the KS-UDFT results for the ^3^*n*,π* states for comparison. The purple arrow in (A) indicates the aromaticity threshold (0.0358). The state orders (T_*n*_ and S_*n*_, *n* = 1, 2, 3,···) given above the bars represent the *n*,π* states. ^a^T_3_ or higher. ^b^Mixed state. See SI Sections S1, S2.1, S3.1, and S3.2 for further details.

The lowest ^3^*n*,π* states of the phenyl and silaphenyl anions (**3** and **4**) have residuals with MCI that lean toward aromaticity; their MCI_β_-components are significantly higher than the corresponding S_0_ values (Figure 4A), and the MCI_α_-components resemble that of pyridine (**1**) in its lowest ^3^π,π* state (0.0026, 0.0011, and 0.0023 for **3**, **4**, and **1**, respectively). In contrast, the lowest ^3^*n*,π* states of **1** and the thiopyrylium cation (**6**) have residuals that lean toward antiaromaticity, while for phosphinine (**2**) the MCI_β_-component of its ^3^*n*,π* state resembles that of S_0_ and should be categorized as nonaromatic. In S_0_, the pyrylium cation (**5**) is nonaromatic because its MCI value is lower than our aromaticity threshold (0.0358). However, in its first ^3^*n*,π* state, the MCI_β_-component is somewhat higher than in the S_0_ state. Still, it is the lowest among the 6-MR monoheteroaromatics, and **5** in its ^3^*n*,π* state should be labeled nonaromatic. Looking instead at the MCI_α_-components of the lowest ^3^*n*,π* states, it is notable that these are similar or just slightly higher (0.0011–0.0030) than the corresponding components in the antiaromatic ^3^π,π* state of these compounds (the MCI_α_-components with four electrons are in the range of −0.0012–0.0034, Table S6, SI). Thus, the π_α_-components of the ^3^*n*,π* states are as antiaromatic as the corresponding component of the ^3^π,π* state of **1** acting as our antiaromaticity reference.

For the lowest ^1^*n*,π* states, we used TD-DFT, for which spin-separation is not possible. Therefore, to assess the quality of the results from TD-DFT, we computed MCI values for the ^3^*n*,π* states using both the KS-UDFT and the TD-DFT formalisms and found that MCI values from TD-DFT are consistently lower by a third to half of the values from KS-UDFT (Figure 4B). This trend is ascribed to the nature of the associated wavefunction in TD-DFT (see Computational Methods in the SI).

Despite this, the trends observed in the TD-DFT results for the lowest ^3^*n*,π* states are very similar to those of KS-UDFT, both in terms of excitation energies and relative (anti)aromaticity assessed by MCI. Thus, we are confident that the ^1^*n*,π* and ^3^*n*,π* states are explored on comparable footings.

The energetic order of the vertical ^1^*n*,π* states matches well that of the corresponding ^3^*n*,π* states, and the total MCI values show that the (anti)aromatic characters in the two states are similar for most 6-MR monoheteroaromatics. The exceptions are **3** and **4** for which the ^1^*n*,π* states are more aromatic than the ^3^*n*,π*, yet, further analysis is impossible as the MCI_α_- and MCI_β_-components cannot be separated in results from TD-DFT computations. Still, for **4** the lowest ^1^*n*,π* state (the S_2_ state) is of mixed *n*,π*/Rydberg character in contrast to the lowest ^3^*n*,π* state which is a pure valence excited state. On the other hand, for **3** the first ^1^*n*,π* state is the 1^1^A_2_ (Type II, Figure 3B), opposite to the other 6-MR monoheteroaromatics for which the lowest ^1^*n*,π* states are 1^1^B_1_. In summary, the extent of (anti)aromaticity of the lowest singlet and triplet *n*,π* states is similar in four of the six 6-MR monoheteroaromatics.

Now, are *n*,π* states with highly aromatic residuals normally T_1_ states, while the T_1_ and S_1_ states are of π,π* character for heteroaromatics which have lowest *n*,π* states with non- or antiaromatic residuals? Figure 4 clarifies that for 6-MR monoheteroaromatics this is the case for some, e.g., **3** with an aromatic residual and T_1_ and S_1_ states of *n*,π* character, and **6** with an antiaromatic residual and T_1_ and S_1_ states of π,π* character. However, **1** with a residual that leans toward antiaromaticity also has T_1_ and S_1_ states of *n*,π* nature. Noteworthy, the order between the lowest ^3^*n*,π* and ^3^π,π* transitions, as well as the type of ^3^*n*,π* state (B_1_ or A_2_), are nearly always the same with UDFT and TD-DFT (Tables S21 and S27, SI), except for **1** and **4** for which the order of ^3^*n*,π* and ^3^π,π* transitions are switched. In summary, the electronegativity of the heteroatom influences the order and energy difference between the *b*_1_ and *a*_2_ symmetric π* orbitals (see below), and consequently, it has a great impact on the order of the ^3^*n*,π* and ^3^π,π* transitions.

In the Introduction, we asked if a more aromatic (antiaromatic) residual of the ^3^*n*,π* state correlates with a lower (higher) vertical excitation energy. However, this hypothesis oversimplifies as the vertical excitation energy also depends on (de)stabilizing features of the S_0_ state, including (anti)aromaticity and several other factors (see Section 2). Still, the monoheteroaromatics with residuals that lean toward aromaticity (**3** and **4**) are the two with lowest *E*(^3^*n*,π*) (2.6 and 2.9 eV, respectively), while thiopyrylium (**6**), with its antiaromatic residual, and the S_0_ nonaromatic pyrylium (**5**) have *E*(^3^*n*,π*) of 5.5 and 5.8 eV, respectively. The same relationship is observed for the ^1^*n*,π* states (3.1 (**3**), 3.8 (**4**), 5.1 (**1**), 5.1 (**2**), 5.7 (**5**), and 6.1 eV (**6**)). However, the *E*(*n*,π*) values also vary with the *n* orbital energies as this orbital is of very low energy for **5** and **6** being the second below the highest occupied molecular orbital (HOMO–2) at 2.09 and 1.78 eV below the highest occupied π-orbital (HOMO), but high for **3** and **4** (for **3** it is as much as 1.89 eV above the highest occupied π-orbital, which is HOMO–1). Also, the highest π and lowest π* orbital energies vary between the heteroaromatics (Table S47, SI). As a result, for **3** the energy differences between the *n* and the b_1_ π* orbital versus the highest π and the b_1_ π* orbital are 7.77 and 9.66 eV, while for **5** these energy differences are 10.44 and 8.36 eV, respectively. Thus, electronegativity variations among the *E* atoms impact strongly on the *n*,π* excitation energies, and these are not necessarily related to (anti)aromatic character.

**Figure 5:**
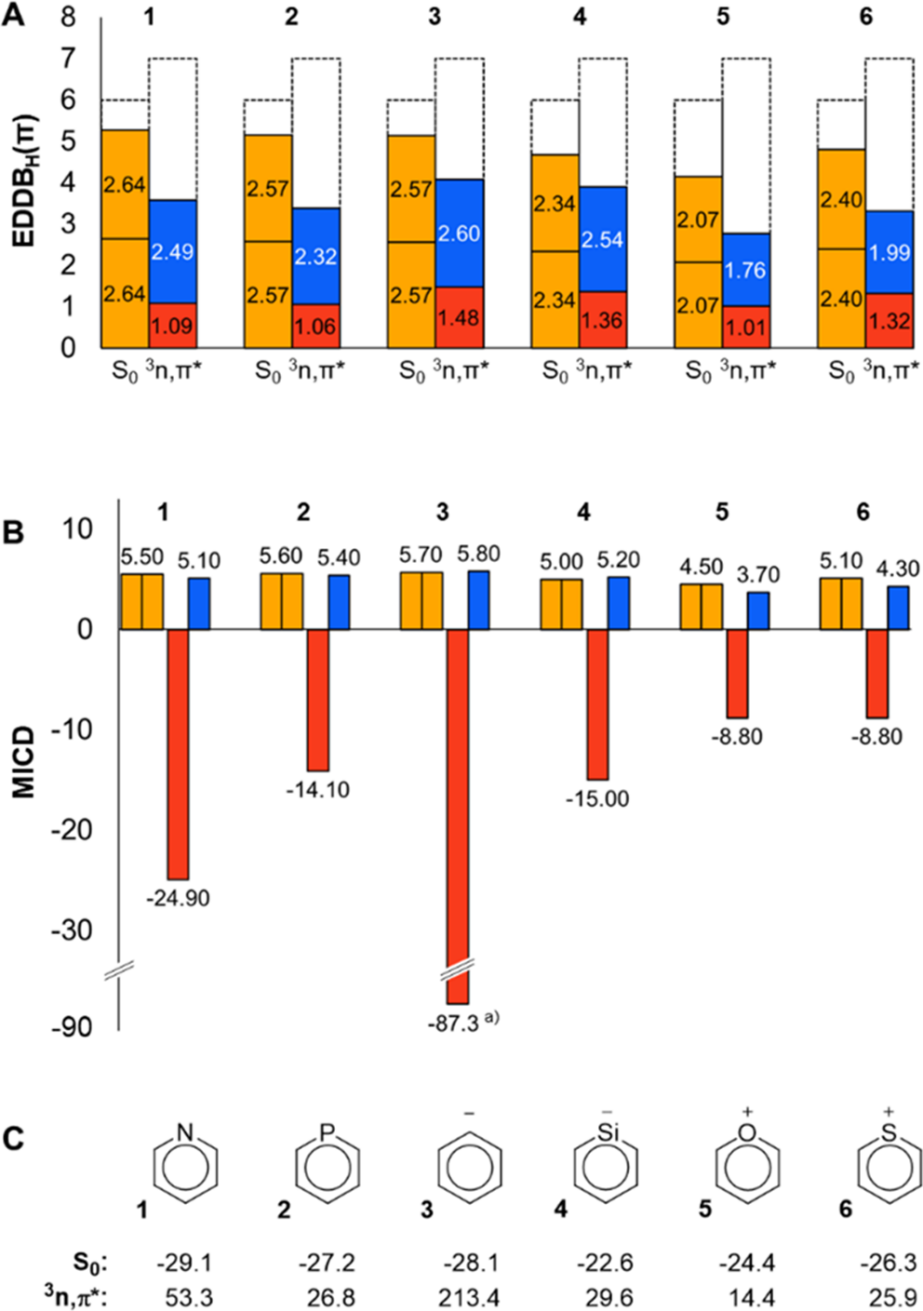
Aromaticity of the monoheteroaromatics in their S_0_ and ^3^*n*,π* states at the UCAM-B3LYP/6-311+G(d,p) level; (A) π-EDDB values (units are electrons), where red and blue bars correspond to, respectively, α- and β-electrons (as references, the total π-EDDB value is 5.33 e for benzene in its aromatic S_0_ state and 2.77 e for pyridine in its antiaromatic ^3^π,π* state, see Section S2.2 in the SI for more details, especially on spin-separated reference values). The dashed line bars show the total number of π-electrons in that state. (B) π-Electron bond current strengths (in nA T^–1^) calculated as the average of all bonds in the given ring, where red and blue bars represent α- and β-electron contributions, respectively. (C) NICS(1)_*zz*_ values (in ppm).

For the ^3^*n*,π* states, EDDB results (Figure 5A) are consistent with the MCI findings. The π_β_-components of **3** and **4** are slightly larger than half of the total π-S_0_ value, indicating aromatic character of the residuals of their ^3^*n*,π* states. In contrast, for the remaining 6-MR monoheteroaromatics, the π_β_-component is lower in the ^3^*n*,π* state than in S_0_. More comprehensive EDDB results, specifically dissecting σ and π contributions, are provided in Section S2.2 in the SI.

**Figure 6:**
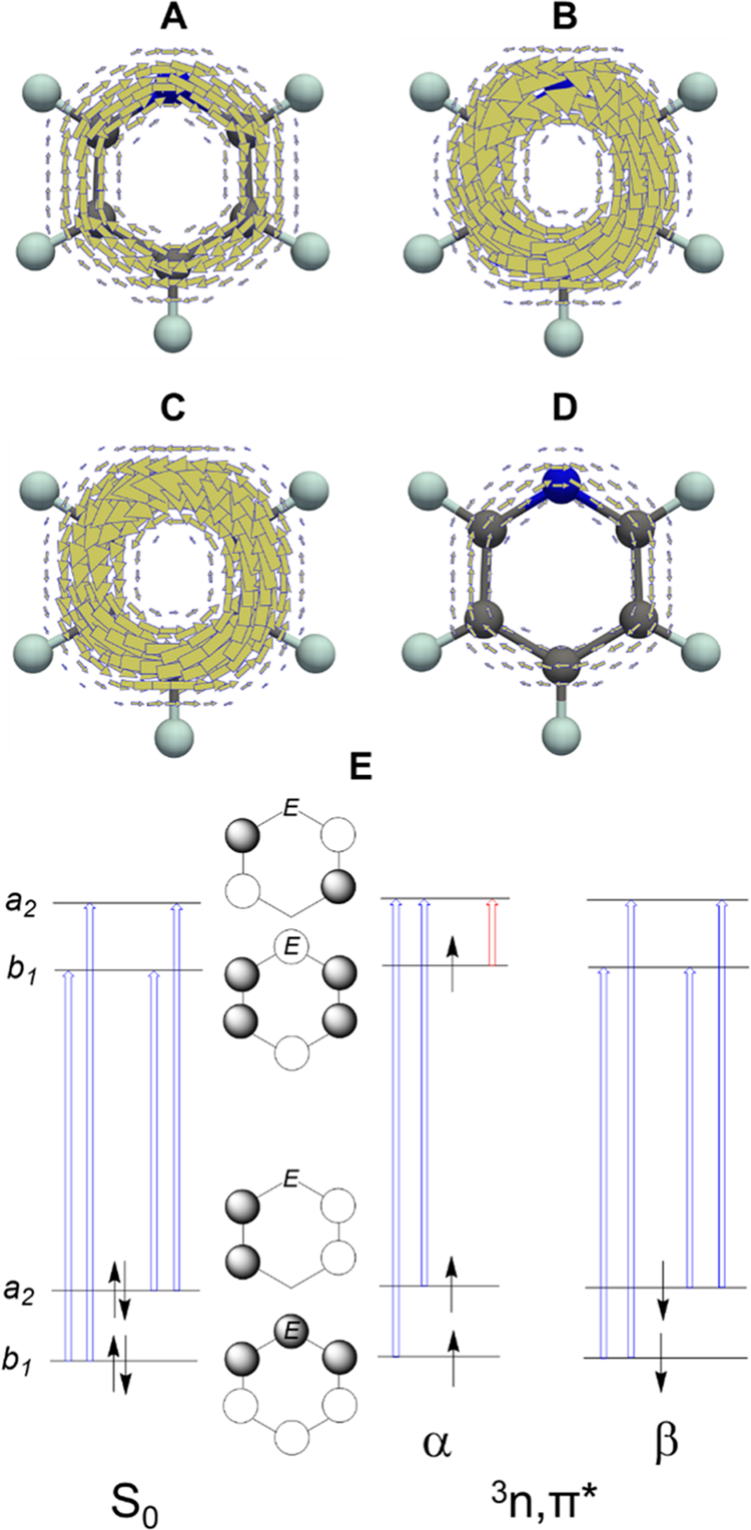
Maps of magnetically induced π-electron current densities calculated at 1 bohr above the molecular plane of **1**: (A) S_0_ state, (B) ^3^*n*,π* state, (C, D) π_α_- and π_β_-electron contributions to the ^3^*n*,π* state. Clockwise (anticlockwise) circulation corresponds to diatropic (paratropic) currents. (E) Qualitative energy level diagram for the frontier molecular orbitals in the S_0_ and ^3^*n*,π* states of monoheteroaromatics. Blue arrows indicate the translationally allowed transitions (inducing diatropic currents), and the red arrow indicates the rotationally allowed transition (inducing paratropic currents). Only the most relevant transitions, based on the values of the linear and angular momentum matrix elements, were selected.

The trend in MICD results (Figure 5B) resembles that of the electronic indices, but the values are markedly offset toward antiaromaticity since the π_α_-components give strong paratropic influences. As an illustration, Figure 6 displays the spin-separated π-electron MICD maps of the S_0_ and ^3^*n*,π* states of **1**, as well as the orbital transition scheme which provides a qualitative rationalization.^57−59^ In S_0_, the π-electrons of all monoheteroaromatics induce diatropic currents due to translational transitions between the occupied b_1_ and a_2_ orbitals and the unoccupied b_1_ and a_2_ orbitals (blue arrows, Figure 6E).^60^ Analogous transitions are found within the π_β_-electron stack of the ^3^*n*,π* state, giving diatropic current contributions. However, the π_α_-electrons induce strongly paratropic currents arising from the rotational transition from the highest occupied b_1_ orbital (the α-SOMO) to the empty a_2_ orbital (red arrow). Although the α-SOMO-1 (a_2_) and α-SOMO-2 (b_1_) contribute to diatropic currents through translational transitions to the unoccupied a_2_ orbital, these contributions are small in comparison to the paratropic current contributions.

Here it should be noted that the relative importance of the various orbital transitions within the π_α_-electron stack is related to the energy differences between the respective occupied and unoccupied orbitals, related to the electronegativity of the *E* atom (see above) and not to (anti)aromaticity. As a consequence, the magnetic (anti)aromaticity of the *n*,π* state may not agree with the electronic, energetic, and geometric aromaticity aspects, similarly as reported for the ^3^π,π* state of B_4_N_4_H_8_^61^ and, in the S_0_ state, for (N_6_H_6_)^2+^ and C_2_N_4_H_6_.^62^ For a detailed analysis of the orbital transitions, see Table S20, SI. It can be noted that the NICS(1)_*zz*_ values (Figure 5C) closely follow the trend of the MICD values, but they provide less information as they are not spin-separated. As a result, **3** in its ^3^*n*,π* state is the most antiaromatic among **1**–**6** with NICS, while the most aromatic with MCI.

Next, we analyze why there is a variation in the (anti)aromatic character of the ^3^*n*,π* states of 6-MR monoheteroaromatics. Both the *E* atom electronegativity and the molecular charge play roles as one can see that a low electronegativity of *E* and a negative charge lead to stronger aromatic character of the residual. The variation also seems to depend on the local p_π_ orbital overlap which differs among the monoheteroaromatics. For this reason, we analyzed the degree of uniformity in the π-electron distribution in the ring by calculating the root-mean-square deviation of π-electron distribution (RMSD(π)) obtained from a natural population analysis (NPA). Interestingly, there are good correlations between the MCI and RMSD(π) values for both S_0_ and ^3^*n*,π*, indicating that the more uniformly distributed the π-electrons, the higher the MCI values (Figure S4, SI). It is further notable that there is a reasonable correlation with the change in the RMSD(π) of the π_β_-electron distribution when going from S_0_ to T_1_ and the degree of (anti)aromaticity of the residual, implying that if the π_β_-electron distribution becomes more (less) uniformly distributed upon excitation, the residual of the system will become more aromatic (antiaromatic).

### 3.3 Diheteroaromatic 6-MRs (Group B)

The analysis becomes more complex for molecules with two heteroatoms as (i) there is a variation in the S_0_ aromaticity with the relative positions of the two heteroatoms,^34,35^ (ii) with two lone pairs there are several *n*,π* states since there are two (near-degenerate) lone-pair orbitals in addition to the two (near-degenerate) π* orbitals (potentially leading to multiconfigurational character), and (iii) there can be a variation among diheteroaromatics as to which *n*,π* state is lowest in energy. To facilitate, we split the diheteroaromatics in two subgroups: one composed of those with two different heteroatoms (**10**–**14**) and one of those with two equal heteroatoms (**7**–**9** and **15**–**19**). Additionally, we consider the protonated species of two compounds in the latter subgroup (**9H**^+^ and **19H**^+^). Those with two different heteroatoms should, viewed simplistically, have the highest *n* orbital dominated by the least electronegative element, and their first *n*,π* state may resemble those of the monoheteroaromatics with the same heteroatom. Yet, we will see that this is not necessarily the case. We calculate MCI throughout the group and MICD for selected compounds.

#### Diheteroaromatic 6-MRs with *E*′ ≠ *E*

Starting with the three azaphosphinines **10**–**12** in their ^3^*n*,π* states computed at UCCSD(T) level, we found that these exhibit some multiconfigurational character (T_1_ diagnostics >0.044, the threshold for open-shell species).^54−56^ Accordingly, we explored these species also at UBD and CASSCF levels and found that multiconfigurational character does not impact on the aromaticity results (see Sections S2.1, S3.1, and S3.6 in the SI). Indeed, the trend in the MCI values computed with UCAM-B3LYP agrees with that of UBD.

**Figure 7:**
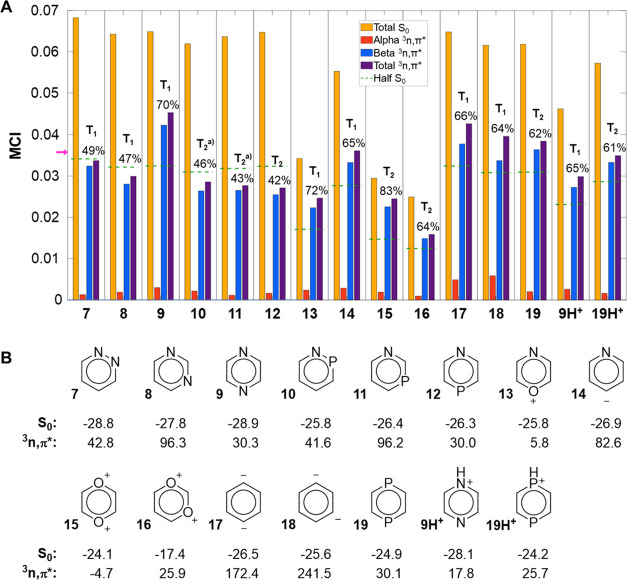
(A) MCI results (in a.u.) of diheteroaromatics in their triplet *n*,π* states (spin-separated) with UCAM-B3LYP/6-311+G(d,p). The purple arrow indicates the aromaticity threshold value for S_0_ (0.0358). The state orders T_*n*_ (*n* = 1, 2, 3,···) is given above the bars that represent the ^3^*n*,π* states. ^a^T_2_ or higher (see SI Sections S2.1 and S3.1 for further details). (B) NICS(1)_*zz*_ values in their S_0_ and ^3^*n*,π* states (in ppm).

**Figure 8:**
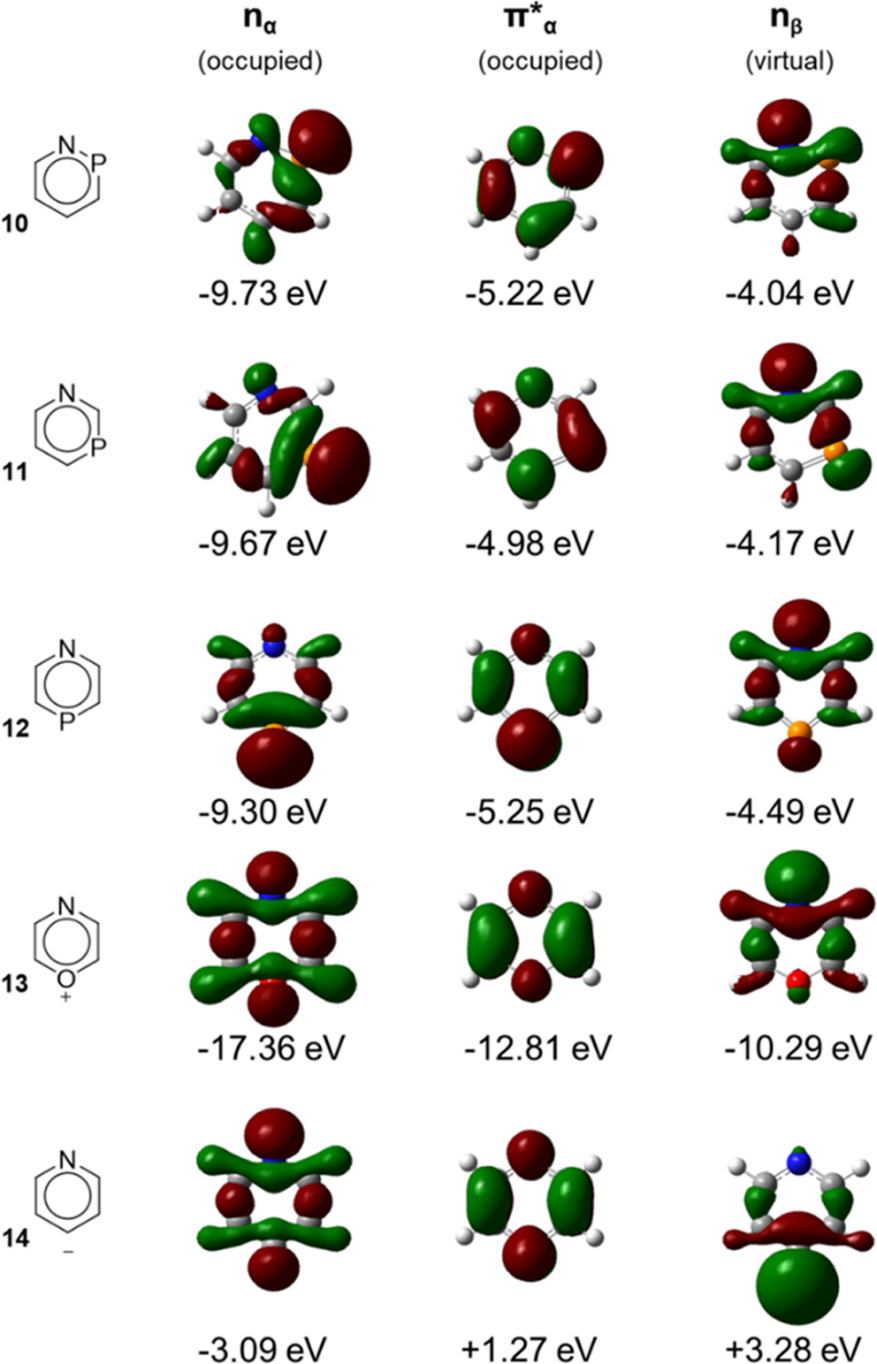
Singly occupied and unoccupied *n* and π* orbitals of **10**–**14** of the ^3^*n*,π* state, with orbital energies in eV. Noteworthy, the virtual lone-pair orbitals in the rightmost column resemble the doubly occupied lone-pair orbitals in S_0_. Isosurfaces of 0.040 au.

In S_0_, there is a minute increase in the aromaticity when going from **10** to **12** according to MCI, in line with earlier findings based on NICS,^45,63−65^ but in the lowest ^3^*n*,π* state, there is instead a minute decrease (Figure 7A). The MCI(^3^*n*,π*)_tot_ values of **10**–**12** are similar to those of **1** and **2** (Figure 4A); however, their residuals (with MCI(^3^*n*,π*)_tot_ at 42–47% of the MCI(S_0_) values) tend toward antiaromatic character whereby they resemble **1** more than **2**. This is consistent with the fact that both C and P have low electronegativity, and that P has been labeled a “carbon copy”.^66^ With regard to the lone-pair orbitals, which are singly occupied in the lowest ^3^*n*,π* states, they are more localized on P than on N (Figure 8), but if one instead views the lone-pair orbital which is unoccupied it resembles the corresponding orbital in S_0_ and is more localized on N than on P.

Although oversimplified, in Section 1, we put forth that the (anti)aromatic character of the residual and the vertical *E*(^3^*n*,π*) may correlate among isomers. Indeed, there is no extensive variation in the (anti)aromatic character neither in the S_0_ states of **10**–**12** nor in the residuals of their ^3^*n*,π* states, and the variation in their vertical *E*(^3^*n*,π*) is modest (3.34–3.60 eV), in line with the postulation. Furthermore, the ^3^*n*,π* states of **10**–**12** are not the T_1_ states but higher states (Figure 7A), a fact that might be explained by the non- or antiaromatic character of the residual of the *n*,π* state. The T_1_ states are instead of π,π* character.

Of the two other diheteroaromatics with *E* ≠ *E*′ (**13** and **14**), it is only **14** that exhibits a sufficiently aromatic character in S_0_ to satisfy our aromaticity criterion (MCI ≥ 0.0358). The ^3^*n*,π* state of **14** resembles that of **3**, whereby also this excitation can be described as that of a monoheteroaromatic. Further support for this interpretation comes from the shape of the formal *n* orbital involved in the *n*,π* excitation since it has a marked localization at the anionic C atom (Figure 8). With regard to **13**, it has a nonaromatic S_0_ state, yet, is still interesting as its MCI value in ^3^*n*,π* is significantly higher than half the S_0_ value. Thus, the MCI(^3^*n*,π*)_tot_ value is intermediate between that of **5** and **1**.

#### Diheteroaromatic 6-MRs with *E*′ = *E*

Among these species, the diazines and compounds **17** and **18** exhibit multiconfigurational character which should stem from near-degeneracy of the two lowest ^3^*n*,π* states (see Tables S25 and S53, SI). However, results from UBD calculations again corroborate that UCAM-B3LYP provides reliable aromaticity results and trends (Tables S8, S11, and S12, SI).

For the diazines **7**–**9**, the aromaticity in S_0_ (Figure 7A) follows the established order pyridazine (**7**) > pyrimidine (**8**) ∼ pyrazine (**9**).^67^ Their lowest ^3^*n*,π* states are the T_1_ states and we find that the excitations are of similar type (Table S24, SI), although a comparison is ambiguous due to their different structures and symmetries. According to MCI, the ^3^*n*,π* state of **9** has a residual with clear aromatic character, while **7** and **8** have nonaromatic residuals (i.e., approximately half of the S_0_ values). Interestingly, although the largest difference in the MCI values between **7**, **8**, and **9** is due to the MCI_β_-component, the MCI_α_ of **9** is also larger than those of **7** and **8**, indicating a weaker π_α_-antiaromatic contribution in the ^3^*n*,π* state of **9**. These findings also agree with those of a recent study on the lowest excited states of the three diazines, yet, where these states were not differentiated as *n*,π* or π,π* states.^24^ It was argued that the more aromatic a molecule is in its S_0_ state, the more antiaromatic it will be in its first excited states. Such relationships were earlier found among the π,π* states of substituted fulvenes and related hyperconjugated compounds,^68^ i.e., specific compound classes. However, we find that this is not valid for the diazines because **7** is more aromatic than **8** in both its S_0_ and T_1_ (^3^*n*,π*) states.

The diazines make it clear that the hypothesis that *E*(^3^*n*,π*) is correlated with the (anti)aromaticity difference between S_0_ and the ^3^*n*,π* states is also oversimplified when regarding only isomers. Among **7**–**9**, the largest difference in (anti)aromaticity between the two states is found for **7** while the smallest is found for **9** (Figure 7A), but **7** has the lowest *E*(^3^*n*,π*) (2.95 eV) and **8** the highest (4.13 eV). Further analysis reveals that the excitation energies are primarily influenced by the relative S_0_ energies of the three isomers because **8** is more stable than **7** by 1.03 eV (Table S24, SI), a feature that stems from repulsion between the lone-pair electrons of the two adjacent N atoms of **7**.^69^

Here we explore why the ^3^*n*,π* state of **9** exhibits a highly aromatic residual according to MCI, and if the same trend among the diazines is found with magnetic and energetic aromaticity descriptors. An energy-based evidence of a higher aromatic character of the ^3^*n*,π* state of **9** than those of **7** and **8** comes from the relative energies, because **7** and **8** in their ^3^*n*,π* states are higher in energy than **9** by, respectively, 0.21 and 0.34 eV. Additionally, and in agreement with the MCI results, the calculated MICD for **7**–**9** demonstrate that only **9** exhibits a somewhat stronger diatropic π_β_-electron ring current in its ^3^*n*,π* state compared to its S_0_ state. Moreover, the α-HSOMO of **9** has the least intensive paratropic contribution among **7**–**9**, in accordance with the values of the α-HOMO–LUMO gap (Table S20, SI). Finally, the NICS values reflect a lower paratropicity of **9** (Figure 7B). Taken together, electronic and energetic descriptors thus support that the ^3^*n*,π* state of **9** has some aromatic character, and the magnetic indicators reveal that **9** has the least antiaromatic T_1_ state among the diazines. In this context, it is notable that perfluoropyridazine in its S_1_ state, which is of ^1^*n*,π* character, photorearranges to the corresponding pyrazine,^70,71^ possibly driven by a gain in aromatic character. Rewardingly, the same trends are found for the S_1_ states (all ^1^*n*,π*) of the diazines as for their T_1_ states (all ^3^*n*,π*), with **9** being the most stable and aromatic isomer also in S_1_ (Tables S13 and S28, SI).

Further clarity on the cause of the aromatic residual is gained by looking into the distribution of the π-electrons. The π-electron population in S_0_ is more evenly spread in **7**, followed by **9**, and last by **8**, in line with the results of Figure 7. In contrast, in the ^3^*n*,π* state of **9** the π-electron distribution is clearly more uniform than in the other two species due to the high symmetry of the former (see Tables S35–S37, SI). In fact, the π_β_-electron population is even more evenly distributed in this state than in the S_0_ state. Looking into the spin distributions, there is an accumulation of the excess π_α_-electrons around the N atoms of **9**. For **7** and **8**, both the π_α_- and the π_β_-electrons are quite localized at the N atoms and at certain C atoms (different for each of the spins), leading to a less uniform π-electron distribution and lowered aromaticity for both spins. Conversely, in *D*_2*h*_ symmetric **9**, despite some accumulation around the N atoms, the distribution of π_β_-electrons is forced by symmetry to be more uniform (all C atoms have the same π-electron population), which explains the aromaticity increase of the MCI_β_-component.

Among the other compounds with *E* = *E*′, **15**, **17**, and **19** have residuals in their ^3^*n*,π* states with considerable aromatic character. Hence, it is obvious that the placement of two equal heteroatoms with in-plane lone-pairs in *para*-positions, leading to *D*_2*h*_ symmetric molecules, provides for *n*,π* states with strong aromatic character of the residuals. For **17** and **18**, it is also notable that the MCI_α_-components are significant, presumably a result of the fact that the excitation involves promotion of an α-electron into an orbital with diffuse character as a result of the low electronegativity of carbon. Although MICD was not computed for these species, NICS supports the MCI results as the *para*-isomers are the least antiaromatic isomers (Figure 7B).

One can note that the *E* atom electronegativity impacts on the S_0_ state aromaticity, and also on the total MCI value of the ^3^*n*,π* state. However, the main factor impacting the MCI value of the ^3^*n*,π* state, relative to the S_0_ state, is the placement of the heteroatoms as the *para*-isomers always have markedly aromatic residuals (Figure 7A). One can also note that the increase mainly occurs in the MCI_β_-components. Furthermore, one may expect that an increased electrostatic repulsion could lead to a more even distribution of the π_β_-electrons among atoms in the 6-MR. Yet, when regarding **9** and **17**, which both are strongly aromatic in S_0_, and which also have strong aromatic character of the residual of the ^3^*n*,π* state, the two species have different π* orbitals and their *n*,π* states are thus of different types (B_1_ in **9** and A_2_ in **17**). Hence, the type of π* orbital, and accordingly, the spatial distribution, seems unrelated to the aromatic character of the residual according to MCI. For these species, we did not explore spin-separated MICD, but with NICS the situation is opposite to that of MCI because with this index compounds **17** and **18** are strongly antiaromatic in their ^3^*n*,π* states (Figure 7B). This feature resembles that observed with NICS for **3** and stems from an extensive paratropic π_α_-contributions.

A further item to note is that compounds with electronegative *E* atoms (primarily O) which are weakly aromatic or nonaromatic in S_0_, gain some aromaticity in the MCI_β_-component in the *n*,π* state (i.e., they obtain values that are more than half of that in S_0_). These species have rather localized π-electrons in S_0_ because of the highly electronegative and electron-deficient O^+^. Upon excitation to the *n*,π* state, the delocalization of the π_β_-electrons increases due to the addition of one electron to the π-system, and the β-component of MCI increases (see Figures 4A and 7A).

Finally, for all diheteroaromatics, we also found a good correlation with the change in the RMSD(π) of the π_β_-electron distribution when going from S_0_ to the ^3^*n*,π* state and the degree of (anti)aromaticity of the residual (Figure S6, SI). In particular, the π-electron distributions in the ^3^*n*,π* states of **9** and **15**, with two equal *E* atoms in *para*-positions, are clearly more uniform than in their isomers due to their *D*_2*h*_ symmetry.

#### Protonated Para-Diheteroaromatics

For the diheteroaromatics with *E* = *E*′ atoms at *para*-positions and with aromatic S_0_ states (**9**, **17**, and **19**) we also explored the changes in (anti)aromatic character upon protonation of their ^3^*n*,π* states. This leads to **9H**^+^, **3**, and **19H**^+^, respectively. These three species remain aromatic in S_0_, but less than when unprotonated. Importantly, though, the residuals of their ^3^*n*,π* states remain aromatic in character in each of these species, indicating that protonation reduces the aromaticity of both the S_0_ and ^3^*n*,π* states to similar relative amounts. In contrast, when **14** is protonated at the negative C atom, leading to **1**, there is no aromatic but rather an antiaromatic residual. From this, we conclude that the two features that lead to an aromatic residual are high symmetry (*D*_2*h*_ or nearly so) and negative charge (excess of electrons).

### 3.4 Generalizations and Tentative Applications

The findings presented above can be generalized. In Section 2, we argued that in an *n*,π* state there is a tug-of-war between the antiaromatic π_α_-component and the aromatic π_β_-component. Is there support for this hypothesis when considering all investigated molecules? We also discussed the potential correlation between the excitation energy and the (anti)aromatic character of the residual but pointed out factors unrelated to (anti)aromaticity that also affect excitation energies. Still, is the hypothesis valid for a limited set of compounds, e.g., substituted derivatives of a certain heteroaromatic? Figures 4 and 7 reveal that heteroaromatics with *n*,π* states with aromatic residuals often have these states as T_1_ or S_1_ states. Yet, is there a connection to the (anti)aromaticity of the residual of the *n*,π* state and the energy difference between the lowest *n*,π* and π,π* states? We further test if the knowledge gained on the (anti)aromatic character of the *n*,π* state can be expanded to other species that are not traditionally seen as heteroaromatics. Finally, we address molecules and features that tentatively can be useful for applications.

Our hypothesis on the molecular relaxation of the ^3^*n*,π* states is shown in Figure 3; the aromatic β-component strives to keep the S_0_ geometry whereas the α-component prefers to distort to release antiaromaticity. To analyze the effect of structural relaxation on the aromaticity of the ^3^*n*,π* states, we optimized the ^3^*n*,π* geometries of **1**–**9**. Compound **5** is, however, not considered since it is nonaromatic in S_0_. In line with the tug-of-war hypothesis, two main behaviors were observed. First, in one set of heteroaromatics (**1**, **2**, **4**, **6**, and **8**, Section S3.5 in the SI), the antiaromaticity of the α-component is reduced significantly, but at the same time, the aromatic β-component is also clearly diminished. For example, during geometry optimization in its ^3^*n*,π* state, **1** puckers and becomes a mixed *n*,π*/π,π* state. In this process, the MCI(^3^*n*,π*)_tot_ relative to the MCI(S_0_) value decreases from 45 to 22%, mainly due to aromaticity loss in the β-component, whereas the paratropic π_α_-electron current decreases from −25.5 to −4.9 nA T^–1^ and the diatropic π_β_-electron current diminishes from 5.6 to 0.2 nA T^–1^. The change in α-contribution is not as pronounced with MCI, as this index cannot clearly differentiate between antiaromaticity and nonaromaticity (see discussions and examples in the MCI and EDDB sections in the SI). In the second set of compounds (**3**, **7**, and **9**), the aromatic β-component remains almost unaffected while the antiaromatic α-component is reduced slightly. For instance, **3** upon relaxation from its vertical ^3^*n*,π* state shifts to a planar, antiquinoidal structure. The residual remains unchanged (the MCI(^3^*n*,π*)_tot_ relative to MCI(S_0_) only decreases from 59 to 58%) while the paratropic π_α_-electron current decreases (from −89.4 to −36.8 nA T^–1^) and the diatropic π_β_-electron current remains almost the same (from 5.9 to 6.1 nA T^–1^). Unfortunately, classification in one or the other set is not related to the extent of (anti)aromatic character of the residual in the vertical ^3^*n*,π* state (e.g., **4** with an aromatic residual and **6** with a residual that tends toward antiaromaticity belong to the same set). Other factors, such as the preferred valence angle of a certain *E* atom or changes in exchange interaction upon excitation, should also play relevant roles.

**Figure 9:**
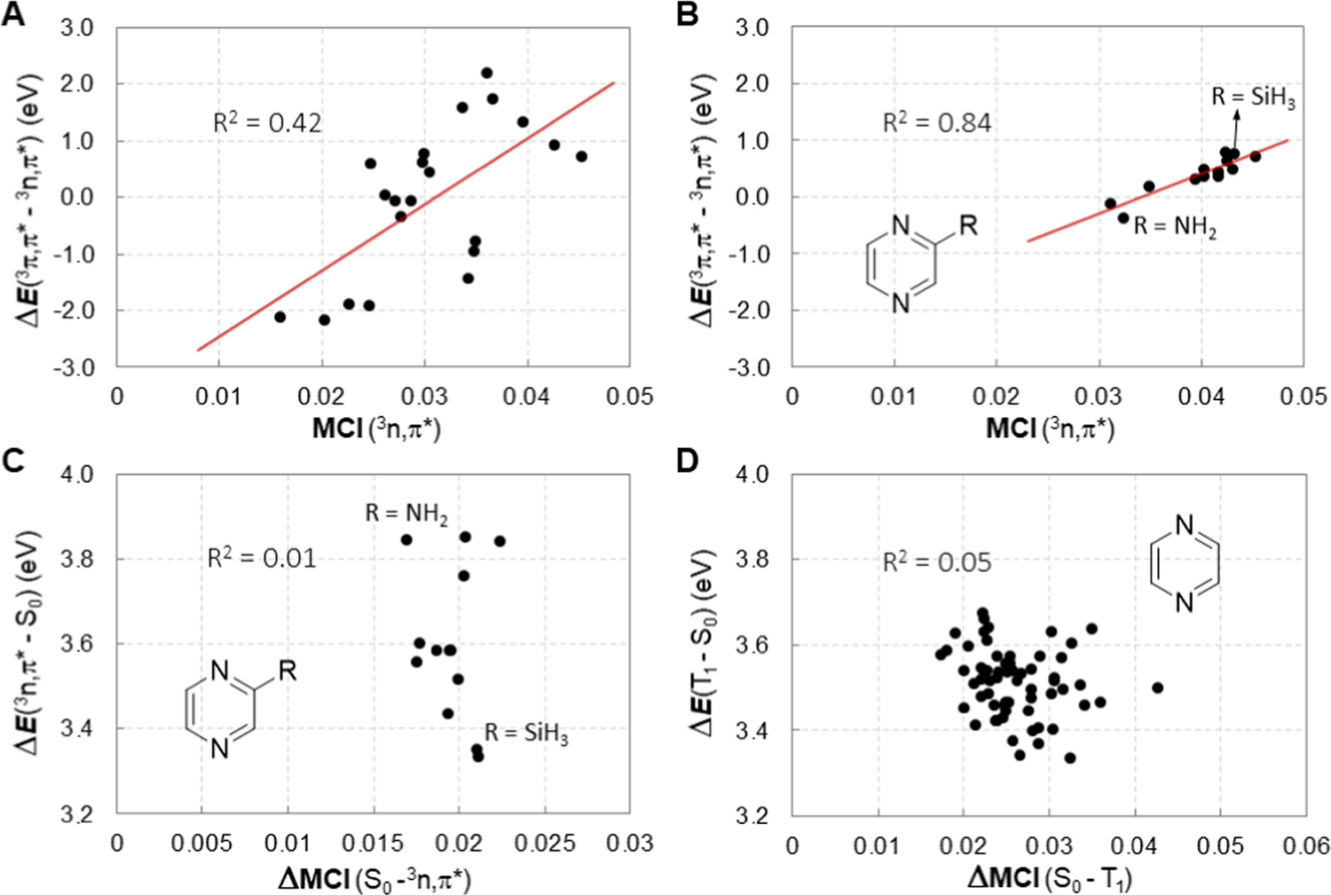
(A) Δ*E*(^3^π,π* – ^3^*n*,π*) vs MCI(^3^*n*,π*) for **1**–**19H**^+^, (B) Δ*E*(^3^π,π* – ^3^*n*,π*) vs MCI(^3^*n*,π*) for **9** and monosubstituted pyrazines, (C) *E*(^3^*n*,π*) vs ΔMCI(S_0_ – ^3^*n*,π*) for **9** and monosubstituted pyrazines, and (D) distorted structures of **9** obtained through a normal mode following algorithm.

By jointly considering the investigated mono- and diheteroaromatics, one can conclude that as a rule-of-thumb a molecule with a high MCI(^3^*n*,π*)_tot_ is likely to have its first ^3^*n*,π* state below the lowest ^3^π,π* state (Figure 9A), although the correlation is weak (*R*^2^ = 0.42). Yet, the energy difference between the ^3^*n*,π* and ^3^π,π* states should depend on both the (anti)aromatic character of the ^3^*n*,π* state and the destabilizing antiaromatic character of the ^3^π,π* state. Our focus herein is however on the *n*,π* states, while the π,π* states need a separate study.

At this point, one can ask if the order between the ^3^*n*,π* and ^3^π,π* states instead (primarily) can be related to orbital energy differences? Indeed, the energy difference between the *n* and lowest π* orbital as compared to the highest π and lowest π* orbital is larger for pyrazine than for pyridine (the orbital energy differences are 8.29 and 8.83 eV for pyrazine and 9.08 and 9.11 eV for pyridine, respectively, Table S48, SI). Yet, there is also a clear effect by the (anti)aromatic character of the ^3^*n*,π* state because for **9** and monosubstituted pyrazines there is a clear correlation (*R*^2^ = 0.84, Figure 9B) between the Δ*E*(^3^π,π* – ^3^*n*,π*) and MCI(^3^*n*,π*), and there is a corresponding correlation between Δ*E*(^3^π,π* – ^3^*n*,π*) and NICS(^3^*n*,π*) although weaker (*R*^2^ = 0.58, Figure S11, SI). Hence, the lowest ^3^*n*,π* states of the species which have these states as their T_1_ states are more aromatic than in the species which have these states as higher excited states (Tables S55–S56, SI). Most monosubstituted pyrazines have T_1_ states of ^3^*n*,π* character (Figure 9B), yet, the π-donating amino substituent leads to a pyrazine with the ^3^π,π* state as T_1_. However, as stated above, to only use the (anti)aromatic character of the ^3^*n*,π* state is an approximation because also the antiaromatic character and energy of the ^3^π,π* state is influenced by substituents.^72^

We also considered **9** and the monosubstituted pyrazines when analyzing the hypothesis on a correlation between the vertical *E*(^3^*n*,π*) and ΔMCI(S_0_ – ^3^*n*,π*), but in this case there is no correlation (Figure 9C). One reason can be partial delocalization of the excitation onto the substituent, similar to previous findings on the ^3^π,π* state of substituted benzenes (*vide supra*).^72^ Still, the amino substituent leads to a pyrazine with high *E*(^3^*n*,π*) while the silyl group does the opposite. We also explored this for **9** at distorted structures with relative energies at most 10 kcal/mol above the optimum, and also in this case we find no correlation (*R*^2^ = 0.05, Figure 9D), likely due to a more significant loss of aromaticity in T_1_ upon distortion than in S_0_ (see SI, Section S3.9).

**Figure 10:**
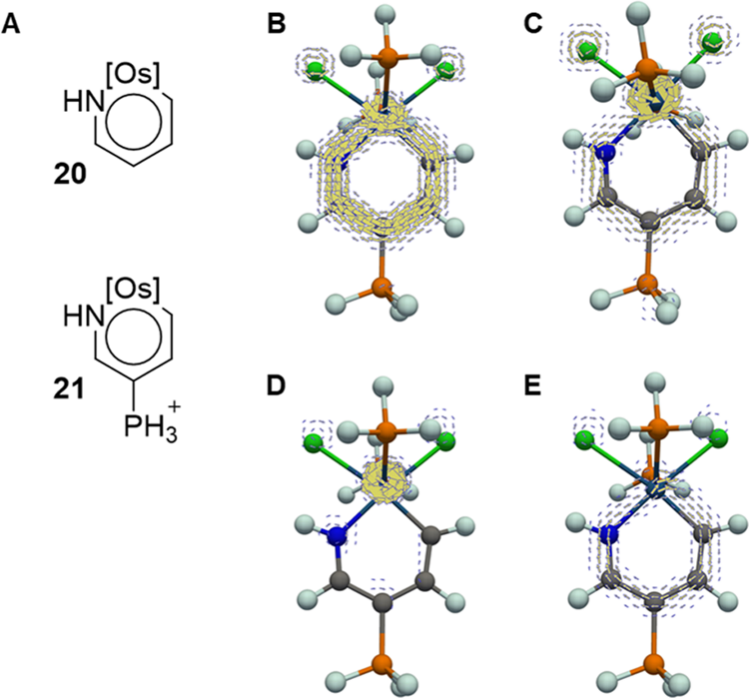
(A) Osmapyridinium complexes **20** and **21**, and (B–E) π-electron MICD plots calculated 1 bohr above the molecular plane of **21**: S_0_ state (B) and vertical ^3^σ,π* state (C) with the corresponding π_α_- and π_β_-electron contributions (D, E). Clockwise circulation corresponds to diatropic (aromatic) currents. Full-scale plots in the SI.

Our theoretical framework for analysis of *n*,π* states can be used to explore molecules with other types of excited states with an odd total number of π-electrons. Osmapyridinium complexes **20** and **21** (Figure 10A) have T_1_ states of ^3^π,σ* and ^3^σ,π* character, and were earlier found to be aromatic in both their S_0_ and T_1_ states (labeled *adaptive aromatic*).^30^ We now analyzed the spin-separated MCI and MICD of both vertical and relaxed ^3^σ,π* states (Figure 10 for MICD and Figure S14 in the SI for MCI results). Of the two species, **21** in ^3^σ,π* should be regarded as aromatic because its MCI(^3^σ,π*)_tot_ value is 57% of the MCI(S_0_) value, while this state for **20** tends toward antiaromaticity (MCI(^3^σ,π*)_tot_ is 39% of the MCI(S_0_) value), or alternatively, as nonaromatic if based on the previously reported MCI results.^30^ The results are mainly due to a relative increase and decrease of the MCI_β_-component, respectively (see Section S3.11 in the SI for more details). Thus, by considering the α- and β-spin components separately, it becomes clear that the phenomenon labeled adaptive aromaticity and used for some metalla-aromatics can be rationalized within the same framework as that of the *n*,π* states of regular heteroaromatics. Thus, adaptive aromaticity is not a new form of aromaticity but a result of an imperfect cancellation of the antiaromatic α- and aromatic β-components as given by Mandado’s rule. Such situations will generally occur for electronic states described as excitations from an in-plane orbital to an out-of-plane orbital (or *vice versa*), or as a one-electron excitation to (or from) one annulenyl fragment from (or to) another fragment of a molecule, e.g., a metal-to-ligand charge transfer state.

Returning to pyrazine, the findings above can explain what we recently found for the photolabile drug amiloride.^73^ Amiloride is a pyrazine derivative with one π-electron withdrawing and three π-electron donating substituents, and this substituent pattern explains why its T_1_ state is of π,π* instead of *n*,π* character. Indeed, its light-induced degradation goes via a sequential two-step photoionization where the ionization step occurs from the antiaromatic T_1_ state of π,π* character.^73^ Such destructive photoionization can possibly be avoided by substituent patterns that instead lead to T_1_ states of ^3^*n*,π* character. For further examples on the tuning of excited state character, see Section S3.10 of the SI on polyazaacenes such as quinoxaline.

Above we found that the high symmetry of pyrazine leads to an ^3^*n*,π* state with an aromatic residual. In this context, 1,3,5-triazine (*s*-triazine) is a *D*_3*h*_ symmetric N-containing heteroaromatic found in several agrochemicals.^8^ Interestingly, the *s*-triazine core in these agrochemicals is inert upon photoexcitation, whereas the substituents change positions. High-level computations also reveal that the lowest few excited states are of *n*,π* character.^74^ Thus, one may postulate that the photostability of *s*-triazine-based agrochemicals stems from aromatic residuals of their *n*,π* states. Yet, as these states are highly multiconfigurational (both the highest *n* orbitals and the lowest π* orbitals are doubly degenerate, Table S48, SI), a proper analysis requires an in-depth computational study.

## 4 Conclusions and Outlook

We examined the *n*,π* states of heteroaromatics with six π-electrons and in-plane lone-pairs (e.g., pyridine and the pyrylium ion), and applied Mandado’s 2*n* + 1 rule for aromaticity of separate spins. In their *n*,π* states, these species have four π_α_-electrons and three π_β_-electrons, which leads to a tug-of-war between the antiaromatic π_α_-component and the aromatic π_β_-component. The component that dominates varies between heteroaromatics and between aromaticity descriptors; the residuals between the two components can lean toward aromaticity or antiaromaticity. Yet, is there a pattern?

We first note that the *n*,π* states of 5-MR mono- and diheteroaromatics (e.g., thiophene and imidazole) lie far above the lowest excited states, which are of π,π* character, and we provide an explanation for this (see Section S3.7 in the SI). In contrast, for 6-MR heteroaromatics with one or two heteroatoms, the *n*,π* states that are T_1_ states often have rather aromatic character, mostly due to a larger β-component than in the Hückel-aromatic S_0_ state. Importantly, similar (anti)aromaticity trends were observed with both the magnetic and the electronic indices, but the antiaromatic character of the α-component is much more dominant in the results of the magnetic indices than in those of the electronic indices.

Also, the (anti)aromatic character of the residual is generally the same for the singlet and triplet *n*,π* states. The heteroaromatics that are likely to exhibit significant aromatic character in their lowest *n*,π* states are molecules with high symmetry (*D*_2*h*_), where electrons become more uniformly distributed, and/or with π-electron donating heteroatoms. Heteroaromatics with such *n*,π* states often have these as their T_1_ states. For the limited set of monosubstituted pyrazines, we observe a significant correlation between the energy difference between the lowest ^3^*n*,π* and ^3^π,π* states and the (anti)aromatic character of the ^3^*n*,π* state, yet, a comprehensive analysis must include also the antiaromatic destabilization of the ^3^π,π* state.

Although the vertical excitation energies of the *n*,π* states depend on several factors of both the S_0_ and *n*,π* states, the relative energies of isomeric heteroaromatics in their *n*,π* states vary in dependence of the aromatic character of the residuals. For example, the *n*,π* state of pyrazine (**9**) has a residual which is more aromatic than the *n*,π* state of pyrimidine (**8**), with the first being lower in energy than the latter by 0.34 eV.

Regarding the geometric relaxation of the *n*,π* state, there is a tug-of-war between the aromatic π_β_-component, which tries to maintain the S_0_ state geometry, and the antiaromatic π_α_-component, which seeks to lessen its antiaromaticity through geometric distortions. Here, we identified two possible scenarios. In some systems, the antiaromaticity of the α-component and the aromaticity of the β-component are reduced significantly, while in others, the aromatic β-component remains almost unaffected while the antiaromatic α-component is reduced only slightly.

The approach described here for *n*,π* states of heteroaromatics can be generalized to analyze the aromaticity of other types of states with an odd total number of π-electrons. It can be utilized to understand the (anti)aromatic character of metallaaromatics which are not only aromatic in S_0_ but also in their triplet σ,π* and π,σ* states, earlier labeled as adaptive aromatic.^29−33^ Our analysis can also be extended to π-conjugated radical anions and cations such as C_6_H_6_^+^ or C_8_H_8_^–^ with an even number of π_α_-electrons and an odd number of π_β_-electrons, or *vice versa*. Indeed, radical cations or anions were analyzed by Mandado and co-workers who found by spin-separated NICS that their π_α_-component were antiaromatic and the π_β_-components aromatic.^19^

Although the findings are fundamental in character, they can be applied. Earlier we found that amiloride-type drugs with central pyrazine moieties photodegrade along a two-step mechanism where the second step is a photoionization with an electron ejected from the antiaromatic T_1_ state of π,π* character.^73^ By knowing that the energetic order between the *n*,π* and π,π* states of pyrazines switch with substituents, and that the *n*,π* states have aromatic (stabilized) character, one may tailor amiloride-like compounds which have *n*,π* states as their T_1_ states. Moreover, with knowledge on how to rationally tune the order between the lowest *n*,π* and π,π* states through substituents, it also becomes clear how to design quinoxolines with lowest *n*,π* states, a feature that should be useful for the design of targeted species with interesting emission properties.

## Supporting Information Available

Aromaticity data; further analysis in terms of energy and electron distribution and additional data; including full-scale plots; input files; compound coordinates; and computational details (PDF)
