# Supplementary material for: The n,π* States of Heteroaromatics: When are They the Lowest Excited States and in What Way Can They Be Aromatic or Antiaromatic?
Source: J Phys Chem A. 2024 May 24;128(22):4493–506. doi: 10.1021/acs.jpca.4c02580 (PMC11163469; doi:10.1021/acs.jpca.4c02580)
Supplement: Supplementary file 1 — jp4c02580_si_001.pdf [file jp4c02580_si_001.pdf]

SUPPORTING INFORMATION

The n,π* States of Heteroaromatics: When are They the Lowest Excited States and in What Way Can They Be Aromatic or Antiaromatic?

Nathalie Proos Vedin,[a] Sílvia Escayola,[b,c] Slavko Radenković,[d]* Miquel Solà,[b]* and Henrik Ottosson[a]*

[a] Department of Chemistry - Ångström Laboratory, Uppsala University, 751 20 Uppsala, Sweden. [b] Institut de Quìmica Computacional i Catàlisi and Departament de Química, Universitat de Girona, C/ Maria Aurèlia Capmany, 69, 17003 Girona, Catalonia, Spain. [c] Donostia International Physics Center (DIPC), 20018 Donostia, Euskadi, Spain. [d] University of Kragujevac, Faculty of Science, P. O. Box 60, 34000 Kragujevac, Serbia.

Table of Contents

1. Computational Methods 3
2. Aromaticity Data 6
   2.1. MCI 6
   2.2. EDDB 13
   2.3. NICS 16
   2.4. MICD 17
3. Further Analysis 19
   3.1. Energies 19
   3.2. TD-DFT results 22
   3.3. Electron Distribution 24
   3.4. Orbital Energies 32
   3.5. Relaxation 33
   3.6. Benchmark Calculations 34
   3.7. 5-MRs 37
   3.8. Order and Energy of Excited States vs. MCI and NICS(1)$_{zz}$ 39
   3.9. Distorted pyrazines 42
   3.10. Polyazaacenes 44
   3.11. Osmapyridinium and Osmapentalene complexes 45
4. Full-Scale MICD Plots 53
5. Input Files and Procedures for Aromaticity Calculations 59
6. Cartesian Coordinates 64

References 65

1. Computational Methods

All geometry optimisations were performed with Gaussian 16 revision B.01 [1], using various density functional theory (DFT) functionals (CAM-B3LYP [2], B3LYP and BLYP [3-5]) and CCSD [6], with the 6-311+G(d,p) basis set of Pople and co-workers [7,8] for all atoms except Os, for which the LANL2DZ basis set was applied [9-11]. In the cases where the T_1 diagnostics values obtained with CCSD were found to be higher than 0.044, the compound was also investigated using Brueckner Doubles (BD), which is a coupled cluster method with only double excitations but allowing for optimization of the molecular orbital (MO) coefficients. In this approach, both the MOs and the coefficients of the expansion in the different excitations are optimized, which makes this method capable of including static correlation [12,13]. Restricted and unrestricted Kohn-Sham (KS) DFT was applied for the closed-shell singlet ground state and the triplet excited states, respectively. The lowest vertically excited states of both singlet and triplet multiplicity were also explored using time-dependent (TD) DFT with the same keywords as with KS-DFT. The triplet TD-DFT calculations were performed by setting the multiplicity to 1 and using the keyword td=triplets.

The general way in which the compounds were investigated was by initial optimisation of the S_0 state, using symmetry, and subsequent single-point calculation of the vertically excited triplet state. In some cases, the relaxed triplet states were also investigated. All minima were verified through frequency calculations. The Gaussian keywords 6d 10f were applied in all energy calculations. In the case where the obtained triplet excited state was not of n,π* character, the orbital ordering was changed using the keyword guess=alter to achieve the correct state symmetry. This is possible since it has been shown that the Hohenberg-Kohn DFT formalism can be generalized to the excited states of lowest energy in each symmetry [14]. In many cases, the other n,π* and the lowest π,π* states were also investigated using this approach. The wavefunctions were found to be stable in all cases except for when the orbital ordering had been altered. For these states, the orbital rotations carried out in the stability analysis forced the wavefunction to leave the symmetry to reach the lower state. Thus, we conclude that the wavefunctions of the states with the orbital order altered are stable within the considered symmetry. When the desired state was obtained, aromaticity calculations using different aromaticity indices, based on electronic, magnetic and geometric criteria, were performed, with separation of the α and β spins where possible. The π-electron distribution in the S_0 and lowest n,π* states was also explored through natural population analysis (NPA), where the natural atomic orbital occupancies were calculated using NBO version 3.1, implemented in Gaussian [15].

Multicenter index (MCI) [16,17], which measures the electron delocalization between different atoms in a compound, was calculated for all compounds and states using the AIMAll [18] and ESI-3D [19,20] packages. Because of the multiconfigurational character of the derived wavefunction in TD-DFT, the MCI value obtained for a given species at the TD-DFT level is always lower than those obtained with the UDFT method with the same basis set. In all cases, MCI is reduced when going from monodeterminantal to correlated wavefunctions due to the fact that correlation localizes the electrons and reduces the delocalization [20-22]. For instance, the MCI of benzene computed at the CAM-B3LYP/6-311+G(d,p) level is 0.0674 a.u. (Table S50) and is reduced to 0.0435 a.u. at the CASSCF(6,6)/6-311++G(d,p) (see Table 2 of Ref. 22).

The electron density of delocalized bonds (EDDB(r)) was used to study π-delocalization in groups A and B [23,24]. In particular, the $EDDB_H(r)$ function was applied, which includes only the contributions from heavy atoms. The charge and bond order matrix required to compute EDDB was calculated using Gaussian 09 revision D.01 together with the NBO 6.0 software package [25,26]. To obtain the dissected σ- and π-$EDDB_H(r)$ index was recomputed considering only the π-natural orbitals for bond delocalization (NOBD). Then, the σ-contribution was obtained as the difference between the total and the π values. The RunEDDB code (version 26-Jun-2021, available upon request: dszczpnk@gmail.com) was used to perform the EDDB analysis and Avogadro 1.2 was employed for the visualization of the NOBDs [27].

Nucleus independent chemical shifts (NICS) [28,29] were obtained using the gauge independent atomic orbitals (GIAO) method [30,31], with the keywords integral=(grid=ultrafine) cphf=(grid=fine) in Gaussian 16. The compounds were all planar and placed in the xy-plane, whereby the perpendicular zz-component of the shielding tensor could be extracted. Here, it is notable that, technically, NICS can be divided into α- and β-components, as done by Mandado [32] using an in-house, non-public code. However, this separation is not routine due to the complexity of the implementation and to specific requirements, and was, thus, only developed for that specific study.

Magnetically induced current densities (MICDs) were calculated using the CTOCD-DZ (continuous transformation of origin of current density method - diamagnetic zero) [33-36] method at CAM-B3LYP/6-311+G(d,p) level of theory. The external magnetic field was applied perpendicularly to the molecular plane. The current density maps were plotted 1 bohr above the ring plane by setting that clockwise/counterclockwise circulations represent diatropic/paratropic current densities. The bond current strengths [37] were obtained by numerical integration of the current densities passing through a rectangle bisecting the bond centre. The integration rectangle starts from the ring centre and extends 5 bohr from the bond centre outside the molecular ring. This rectangle spreads 5 bohr above and 5 bohr below the ring plane. Ring current strengths were calculated as the average current strengths of all bonds in the given ring.

The harmonic oscillator model of aromaticity (HOMA) was applied to a few states to assess the aromaticity with an indicator based on geometric properties [38,39].

All CASSCF calculations were performed using OpenMolcas (version 22.10-2021b) [40]. The (8,8) or (10,10) active spaces, including 6 π-orbitals (3 bonding and 3 antibonding) and the 2 n-orbitals, for pyridine (**1**) and pyrylium (**4**), or the 4 n-orbitals for the rest of the molecules, (see Figure S7) together with the 6-311G(d,p) basis set were used.

Wigner and thermal sampling were employed to study the effects of geometric distortion on the aromaticity of pyrazine. The ensemble of distorted geometries was generated using normal mode-following algorithms: JProgdyn [41] and Wigner sampling as implemented in SHARC package [42]. The first step involves a CAM-B3LYP/6-311+G(d,p) optimization and frequency analysis with G16. Stochastic displacements in each vibrational mode were performed based on both Boltzmann-distributed and Wigner-distributed initial conditions. The energy range for

the ground state of these distorted geometries spanned from 5.45 to 74.9 kcal/mol with respect to the equilibrium geometry, with a mean value of 22.29 kcal/mol.

Given that the Wigner sampling tends to give a broad range of distorted geometries [43], we found only a limited number of structures (out of the total 1600 generated geometries) with distortions under 10 kcal/mol. In order to increase the number of structures within our 10 kcal/mol threshold, we performed Boltzmann sampling to generate 300 structures, which yielded a narrower range of distortions. This approach allowed us to extract a larger set of geometries with distortions falling below the threshold yielding a total of 62 geometries, coming from both approaches, that have been used for the correlation in Figure 9D.

2. Aromaticity Data

2.1. MCI

In this section, the total (σ+π) MCI values for all compounds considered in the main text are tabulated. The results have been split into MCI_{α} and MCI_{β} contributions where possible (when an unrestricted DFT approach was used). At the end of the section, the MCI results were separated into σ and π contributions for selected compounds.

Electronic indices, such as MCI, measures the amount of delocalized electrons throughout the ring. A higher value indicates that the species is more aromatic. For example, the MCI value for benzene in its (aromatic) ground state is with CAM-B3LYP/6-311+G(d,p) calculated to be 0.0716, whereas that of its (antiaromatic) T_1 state is -0.0041. Similar results were also found by Feixas et al. [22] at the SA-CASSCF(6,6)/6-311++G(d,p) level of theory, with MCI being practically zero.

However, differentiating between antiaromaticity and nonaromaticity is less straightforward, as both situations are represented by lower values (more localized electrons), typically very small numbers close to zero. [44,45] As an example, cyclobutadiene (CBD) with its four π-electrons is most antiaromatic in S_0 at its square structure with D_{4h} symmetry. In order to alleviate part of this destabilization, the S_0 state structure distorts away from its quadratic geometry to a rectangular structure with D_{2h} symmetry. Upon further elongation of the longer side of the resulting rectangle, CBD is expected to become even more nonaromatic (less antiaromatic). Interestingly, elongation of the C–C single bond from 1.57 Å (the bond length at the optimized geometry) to 1.80 Å (keeping the C=C double bond length constant) results in a slight lowering the MCI value of the compound, from 0.0095 to 0.0068, at CAM-B3LYP/6-311+G(d,p) level of theory. Thus, the MCI value for the expanded CBD in S_0 would at first glance correspond to a slightly more antiaromatic character due to the slightly lower MCI value compared to that of the minimum geometry. However, it needs to be realized that at the extreme point for the rectangular distortion, corresponding to two infinitely separated acetylene molecules, the MCI value is exactly zero. That is, MCI cannot differentiate between a nonaromatic molecular system with disrupted electron delocalization and a fully conjugated antiaromatic one. In conclusion, differences in MCI values between antiaromatic and nonaromatic species are minor and, therefore, MCI does not differentiate between such compounds.

2.1.1. Group A (Monoheteroaromatics)

In Tables S1-S5, MCI results for the S_0 and the lowest vertical ^{3}n,π* state of the compounds of group A are presented. In S_0, the MCI_{α} and MCI_{β} are just half of the total value, which is shown in the third column of each table. For the ^{3}n,π* state, the spin-separated data is calculated and shown explicitly, along with how these compare with the corresponding S_0 value.

Table S6 shows the same, but for the lowest vertical $^3\pi,\pi^*$ states instead of the $^3n,\pi^*$ state. Although these values in most cases are found to be negative, the percentages are still kept to aid in the analysis.

In Table S7, the MCI data for the lowest singlet and triplet n,π* states obtained with TD-DFT are presented. In this case, there is no S_0 state data to compare with.

Table S1. MCI results of the S_0 and the lowest vertical $^3n,\pi^*$ state of group A, calculated at CAM-B3LYP/6-311+G(d,p) level of theory.

	**S_0**		**Lowest vertical $^3n,\pi^*$**					
**Compound**	**MCI**	**50% of MCI(S_0)**	**MCI**	**% of MCI(S_0)**	**MCI_α**	**% of MCI(S_0)**	**MCI_β**	**% of MCI(S_0)**
**1**	0.0674	0.0337	0.0304	45%	0.0028	4%	0.0276	41%
**2**	0.0678	0.0339	0.0348	51%	0.0016	2%	0.0332	49%
**3**	0.0619	0.031	0.0366	59%	0.0026	4%	0.0339	55%
**4**	0.0419	0.021	0.0261	62%	0.0011	3%	0.0250	60%
**5**	0.0293	0.0146	0.0203	69%	0.0030	10%	0.0173	59%
**6**	0.0562	0.0281	0.0226	40%	0.0029	5%	0.0197	35%

Table S2. MCI results of the S_0 and the lowest vertical $^3n,\pi^*$ state of group A, calculated at B3LYP/6-311+G(d,p) level of theory.

	**S_0**		**Lowest vertical $^3n,\pi^*$**					
**Compound**	**MCI**	**50% of MCI(S_0)**	**MCI**	**% of MCI(S_0)**	**MCI_α**	**% of MCI(S_0)**	**MCI_β**	**% of MCI(S_0)**
**1**	0.0679	0.0340	0.0314	46%	0.0028	4%	0.0286	42%
**2**	0.0686	0.0343	0.0352	51%	0.0015	2%	0.0337	49%
**3**	0.0624	0.0312	0.0368	59%	0.0027	4%	0.0341	55%
**4**	0.0441	0.0220	0.0272	62%	0.0013	3%	0.0259	59%
**5**	0.0304	0.0152	0.0218	72%	0.0031	10%	0.0187	62%
**6**	0.0566	0.0283	0.0266	47%	0.0029	5%	0.0237	42%

Table S3. MCI results of the S_0 and the lowest vertical $^3n,\pi^*$ state of group A, calculated at BLYP/6-311+G(d,p) level of theory.

	**S_0**		**Lowest vertical $^3n,\pi^*$**					
**Compound**	**MCI**	**50% of MCI(S_0)**	**MCI**	**% of MCI(S_0)**	**MCI_α**	**% of MCI(S_0)**	**MCI_β**	**% of MCI(S_0)**
**1**	0.0704	0.0352	0.0335	48%	0.0025	4%	0.0310	44%
**2**	0.0707	0.0353	0.0360	51%	0.0012	2%	0.0348	49%
**3**	0.0643	0.0321	0.0374	58%	0.0026	4%	0.0348	54%
**4**	0.0468	0.0234	0.0285	61%	0.0014	3%	0.0271	58%
**5**	0.0350	0.0175	0.0242	69%	0.0030	9%	0.0212	61%
**6**	0.0582	0.0291	0.0305	52%	0.0027	5%	0.0278	48%

Table S4. MCI results of the S_0 and the lowest vertical $^3n,\pi^*$ state of group A, calculated at CCSD/6-311+G(d,p) level of theory.

	S_0		Lowest vertical $^3n,\pi^*$					
**Compound**	**MCI**	**50% of MCI(S_0)**	**MCI**	**% of MCI(S_0)**	**MCI_α**	**% of MCI(S_0)**	**MCI_β**	**% of MCI(S_0)**
**1**	0.0443	0.0222	0.0206	47%	0.0021	5%	0.0185	42%
**2**	0.0431	0.0215	0.0237	55%	0.0012	3%	0.0226	52%
**3**	0.0415	0.0207	0.0250	60%	0.0020	5%	0.0230	55%
**4**	0.0275	0.0138	0.0168	61%	0.0009	3%	0.0159	58%
**5**	0.0188	0.0094	0.0110	59%	0.0022	11%	0.0089	47%
**6**	0.0384	0.0192	0.0132	34%	0.0021	5%	0.0111	29%

Table S5. MCI results of the S_0 and the lowest vertical $^3n,\pi^*$ state of group A, calculated at BD/6-311+G(d,p)//CCSD/6-311+G(d,p) level of theory.

	S_0		Lowest vertical $^3n,\pi^*$					
**Compound**	**MCI**	**50% of MCI(S_0)**	**MCI**	**% of MCI(S_0)**	**MCI_α**	**% of MCI(S_0)**	**MCI_β**	**% of MCI(S_0)**
**1**	0.0446	0.0223	0.0202	45%	0.0021	5%	0.0182	41%
**3**	0.0417	0.0209	0.0248	59%	0.0020	5%	0.0228	55%
**5**	0.0190	0.0095	0.0119	63%	0.0021	11%	0.0098	51%
**6**	0.0388	0.0194	0.0133	34%	0.0021	5%	0.0112	29%

Table S6. MCI results of the S_0 and the lowest vertical $^3\pi,\pi^*$ states of group A, calculated at CAM-B3LYP/6-311+G(d,p) level of theory. The labels a and b correspond to excitation out of the two possible π orbitals.

	S_0		Lowest vertical $^3\pi,\pi^*$					
**Compound**	**MCI**	**50% of MCI(S_0)**	**MCI**	**% of MCI(S_0)**	**MCI_α**	**% of MCI(S_0)**	**MCI_β**	**% of MCI(S_0)**
**1a**	0.0674	0.0337	-0.0005	-1%	0.0023	3%	-0.0028	-4%
**1b**	0.0674	0.0337	-0.0051	-8%	0.0026	4%	-0.0077	-11%
**2a**	0.0678	0.0339	-0.0103	-15%	-0.0001	0%	-0.0102	-15%
**2b**	0.0678	0.0339	-0.0010	-2%	0.0010	1%	-0.0020	-3%
**3a**	0.0619	0.0310	-0.0064	-10%	0.0022	4%	-0.0086	-14%
**3b**	0.0619	0.0310	0.0005	1%	0.0024	4%	-0.0019	-3%
**4a**	0.0419	0.0210	-0.0131	-31%	-0.0012	-3%	-0.0119	-28%
**4b**	0.0419	0.0210	-0.0010	-2%	0.0004	1%	-0.0014	-3%
**5a**	0.0293	0.0146	0.0042	14%	0.0034	12%	0.0008	3%
**5b**	0.0293	0.0146	-0.0012	-4%	0.0022	8%	-0.0034	-12%
**6a**	0.0562	0.0281	-0.0011	-2%	0.0021	4%	-0.0032	-6%
**6b**	0.0562	0.0281	-0.0060	-11%	0.0022	4%	-0.0082	-15%

Table S7. MCI results of the S_0 and the lowest vertical ^{1}n,π* and ^{3}n,π* states of group A, calculated at TD-CAM-B3LYP/6-311+G(d,p) level of theory. The states are all of type I (Figure 3B) unless otherwise stated.

Compound	State	MCI
**1**	T_2	0.0191
	S_1	0.0173
**2**	T_2	0.0192
	S_2	0.0187
**3**	T_1	0.0206
	S_1 (type II)	0.0232
	S_4 (type I)	0.0247
**4**	T_2	0.0146
	S_2	0.0205
**5**	T_4	0.0113
	S_2	0.0105
**6**	T_4	0.0145
	S_3	0.0133

2.1.2. Group B (Diheteroaromatics)

Table S8. MCI results of the S_0 and the lowest vertical ^{3}n,π* state of group B, calculated at CAM-B3LYP/6-311+G(d,p) level of theory. Compounds **17** and **18** are of a different type compared to the rest.

	S_0		Lowest vertical ^{3}n,π*					
**Compound**	**MCI**	**50% of MCI(S_0)**	**MCI**	**% of MCI(S_0)**	**MCI$_\alpha$**	**% of MCI(S_0)**	**MCI$_\beta$**	**% of MCI(S_0)**
**7**	0.0682	0.0341	0.0337	49%	0.0013	2%	0.0324	47%
**8**	0.0643	0.0321	0.0299	47%	0.0019	3%	0.0280	44%
**9**	0.0648	0.0324	0.0453	70%	0.0030	5%	0.0423	65%
**10**	0.0619	0.031	0.0286	46%	0.0022	4%	0.0264	43%
**11**	0.0636	0.0318	0.0277	43%	0.0011	2%	0.0265	42%
**12**	0.0647	0.0323	0.0271	42%	0.0016	3%	0.0255	39%
**13**	0.0342	0.0171	0.0247	72%	0.0024	7%	0.0223	65%
**14**	0.0553	0.0276	0.0361	65%	0.0029	5%	0.0332	60%
**15**	0.0294	0.0147	0.0245	83%	0.0019	7%	0.0225	77%
**16**	0.0249	0.0125	0.0159	64%	0.0010	4%	0.0149	60%
**17**	0.0648	0.0324	0.0426	66%	0.0049	8%	0.0377	58%
**18**	0.0616	0.0308	0.0395	64%	0.0059	10%	0.0337	55%
**19**	0.0618	0.0309	0.0342	55%	0.0015	2%	0.0327	53%
**9H$^+$**	0.0462	0.0231	0.0298	65%	0.0026	6%	0.0272	59%
**19H$^+$**	0.0573	0.0286	0.0349	61%	0.0016	3%	0.0333	58%

Table S9. MCI results of the S_0 and the lowest vertical $^3n,\pi^*$ state of group B, calculated at B3LYP/6-311+G(d,p) level of theory.

	S_0		Lowest vertical $^3n,\pi^*$					
**Compound**	**MCI**	**50% of MCI(S_0)**	**MCI**	**% of MCI(S_0)**	**MCI_α**	**% of MCI(S_0)**	**MCI_β**	**% of MCI(S_0)**
**7**	0.0694	0.0347	0.0346	50%	0.0014	2%	0.0332	48%
**8**	0.0655	0.0327	0.0309	47%	0.0019	3%	0.029	44%
**9**	0.0656	0.0328	0.0449	68%	0.0031	5%	0.0418	64%
**10**	0.0634	0.0317	0.0308	49%	0.0021	3%	0.0286	45%
**11**	0.0646	0.0323	0.0268	42%	0.0000	0%	0.0269	42%
**12**	0.0663	0.0332	0.0341	51%	0.0011	2%	0.0331	50%

Table S10. MCI results of the S_0 and the lowest vertical $^3n,\pi^*$ state of group B, calculated at BLYP/6-311+G(d,p) level of theory.

	S_0		Lowest vertical $^3n,\pi^*$					
**Compound**	**MCI**	**50% of MCI(S_0)**	**MCI**	**% of MCI(S_0)**	**MCI_α**	**% of MCI(S_0)**	**MCI_β**	**% of MCI(S_0)**
**7**	0.0722	0.0361	0.0364	50%	0.0013	2%	0.035	49%
**8**	0.0693	0.0347	0.0328	47%	0.0017	2%	0.0312	45%
**9**	0.0691	0.0346	0.0457	66%	0.0031	4%	0.0427	62%
**10**	0.0669	0.0334	0.0224	33%	0.0014	2%	0.0210	31%
**11**	0.0679	0.0339	0.0089	13%	-0.0013	-2%	0.0103	15%
**12**	0.0698	0.0349	0.0365	52%	0.0007	1%	0.0357	51%

Table S11. MCI results of the S_0 and the lowest vertical $^3n,\pi^*$ state of group B, calculated at CCSD/6-311+G(d,p) level of theory. Compound **19H$^+$** is modestly non-planar in its S_0 geometry, with the atoms of the P-H unit being slightly out-of-plane.

	S_0		Lowest vertical $^3n,\pi^*$					
**Compound**	**MCI**	**50% of MCI(S_0)**	**MCI**	**% of MCI(S_0)**	**MCI_α**	**% of MCI(S_0)**	**MCI_β**	**% of MCI(S_0)**
**7**	0.0436	0.0218	0.0248	57%	0.0009	2%	0.0239	55%
**8**	0.0420	0.021	0.0195	46%	0.0013	3%	0.0182	43%
**9**	0.0419	0.021	0.0290	69%	0.0023	5%	0.0267	64%
**10**	0.0394	0.0197	0.0190	48%	0.0017	4%	0.0172	44%
**11**	0.0398	0.0199	0.0199	50%	0.0013	3%	0.0185	47%
**12**	0.0410	0.0205	0.0159	39%	0.0013	3%	0.0146	36%

**13**	0.0217	0.0108	0.0166	76%	0.0016	7%	0.0149	69%
**14**	0.0364	0.0182	0.0246	68%	0.0022	6%	0.0224	61%
**15**	0.0184	0.0092	0.0169	92%	0.0013	7%	0.0156	85%
**16**	0.0157	0.0078	0.0103	66%	0.0007	4%	0.0096	61%
**17**	0.0432	0.0216	0.0283	65%	0.0036	8%	0.0247	57%
**18**	0.0414	0.0207	0.0282	68%	0.0039	9%	0.0243	59%
**19**	0.0370	0.0185	0.0205	55%	0.0010	3%	0.0195	53%
**$9H^+$**	0.0296	0.0148	0.0213	72%	0.0018	6%	0.0195	66%
**$19H^+$**	0.0331	0.0165	0.0238	72%	0.0013	4%	0.0225	68%

Table S12. MCI results of the S_0 and the lowest vertical ^{3}n,π* state of group B, calculated at BD/6-311+G(d,p)//CCSD/6-311+G(d,p) level of theory.

	**S_0**		**Lowest vertical ^{3}n,π***					
**Compound**	**MCI**	**50% of MCI(S_0)**	**MCI**	**% of MCI(S_0)**	**MCI_α**	**% of MCI(S_0)**	**MCI_β**	**% of MCI(S_0)**
**7**	0.0439	0.0220	0.0235	53%	0.0010	2%	0.0225	51%
**8**	0.0424	0.0212	0.0199	47%	0.0014	3%	0.0185	44%
**9**	0.0422	0.0211	0.0310	73%	0.0022	5%	0.0288	68%
**10**	0.0398	0.0199	0.0182	46%	0.0016	4%	0.0165	42%
**11**	0.0402	0.0201	0.0184	46%	0.0011	3%	0.0173	43%
**12**	0.0414	0.0207	0.0153	37%	0.0013	3%	0.0140	34%
**15**	0.0186	0.0093	0.0171	92%	0.0158	85%	0.0158	85%
**16**	0.0160	0.0080	0.0106	66%	0.0007	4%	0.0099	62%
**17**	0.0435	0.0217	0.0288	66%	0.0037	9%	0.0251	58%
**18**	0.0417	0.0209	0.0267	64%	0.0041	10%	0.0225	54%

Table S13. MCI results of the S_0 and the lowest vertical ^{1}n,π* and ^{3}n,π* states of group B, calculated at TD-CAM-B3LYP/6-311+G(d,p) level of theory.

**Compound**	**State**	**MCI**
**7**	T_1	0.0173
	S_1	0.0158
**8**	T_1	0.0186
	S_1	0.0176
**9**	T_1	0.0239
	S_1	0.0238

2.1.3. σ- and π-contributions

We find that σ-contributions to the MCI values are negligible (less than 2% in almost all systems and up to 5% and 7% for the ^{3}n,π* states of **6** and **12**, respectively), except for the ^{3}n,π* states of **5**, **15**, and **16** for which the $MCI_σ$ (basically $MCI_{σβ}$) represents up to 25-30% of the total MCI value. It is worth noting that the S_0 states of **5**, **15**, and **16** are nonaromatic and, therefore, the σ-contributions do not modify the conclusion about the lack of aromaticity of these ^{3}n,π*states.

Table S14. MCI results, dissected into σ- and π-contributions**,** of the S_0 and the lowest vertical ^{3}n,π* state of compounds **1**, **5**, **6**, **12**, **15**, **16**, and **17**, calculated at CAM-B3LYP/6-311+G(d,p) level of theory.

	**S_0**			**Lowest vertical ^{3}n,π***				
**Compound**	**MCI**	**$MCI_π$**	**$MCI_σ$**	**MCI**	**$MCI_{πα}$**	**$MCI_{πβ}$**	**$MCI_{σα}$**	**$MCI_{σβ}$**
**1**	0.0674	0.0665	0.0009	0.0304	0.0025	0.0273	0.0003	0.0003
**5**	0.0293	0.0286	0.0007	0.0203	0.0027	0.0124	0.0003	0.0049
**6**	0.0562	0.0554	0.0008	0.0226	0.0025	0.0189	0.0004	0.0008
**12**	0.0647	0.0640	0.0007	0.0271	0.0014	0.0238	0.0002	0.0017
**15**	0.0294	0.0288	0.0005	0.0245	0.0017	0.0154	0.0002	0.0071
**16**	0.0249	0.0247	0.0003	0.0159	0.0008	0.0102	0.0002	0.0047
**17**	0.0648	0.0639	0.0009	0.0426	0.0045	0.0341	0.0004	0.0036

2.2. EDDB

In archetypal small aromatic monocyclic systems EDDB takes values close to the number of π-electrons, *i.e.* 6 electrons for benzene. As described for MCI above, the quantification of antiaromaticity using indices based on electron delocalization is not straightforward, since antiaromatic molecules still exhibit delocalized electrons to a minor extent. In the case of EDDB, for antiaromatic molecules we obtain intermediate values between aromatic and nonaromatic (the latter present values close to 0). For instance, benzene at T_1 state ($^3\pi\pi^*$ state, where one electron has been promoted from the π_β to the π^*_α orbital, so it is predicted to be antiaromatic according to Baird's rule) gives EDDB values around half of the corresponding aromatic molecule at S_0 (Table S15). Likewise, in the spin-separated case, for an aromatic situation with three π_α (or π_β)-electrons one should expect a π-EDDB value slightly lower than three, whereas for an antiaromatic situation with four π_α (or π_β)-electrons, one should expect a π-EDDB value lower than two.

Table S15. Expected number of π-electrons and total (σ+π) $EDDB_H$ values and dissected into α- and β-components, for benzene in the S_0 and T_1 ($^3\pi,\pi^*$) states as well as pyridine in the T_1 ($^3\pi,\pi^*$) state. The calculations have been done at CAM-B3LYP/6-311+G(d,p) level of theory.

		**π-electrons**	**EDDB**	**$EDDB_\pi$**	**$EDDB_\sigma$**
Benzene	S_0	6	5.57	5.33	0.24
	α	3	2.78	2.67	0.12
	β	3	2.78	2.67	0.12
	T_1	6	3.27	3.02	0.25
	α	4	1.78	1.65	0.13
	β	2	1.49	1.37	0.12
Pyridine	T_1	6	3.21	2.77	0.44
	α	4	1.70	1.48	0.22
	β	2	1.51	1.29	0.22

Taking into account the previous considerations, we will analyze the data obtained for compounds of groups A and B. It is important to highlight that, while EDDB identifies from ~1.0 to 2.0 delocalized electrons out of the four π_α-electrons (Tables S16 and S17), the corresponding MCI values are near-zero. Thus, direct comparisons between EDDB and MCI can be misleading due to the different magnitudes of the two indices. However, EDDB and MCI indices can still be used in a similar manner to compare the aromaticity of compounds within a group. For group A, EDDB results are consistent with MCI (Tables S1 and S16).

Compounds **4-6** exhibit β-component values that in the $^3n\pi^*$ are slightly larger than half of the S_0 values (Table S16). Among these, **4** is the only one that exhibits a π_β-value larger than half of the π-S_0 value. While, in **5** and **6**, the larger β-component in $^3n\pi^*$ is due to an increase of delocalization of σ-electrons. Actually, the σ-delocalization is only considerable in the case of **5**, as shown in the representation of the $EDDB_\sigma$ surfaces (Figure S1). Moreover, the mean

values of the π-NOBD populations in ^{3}nπ* for systems **1-4** are close to 1, while for **5** and **6** these values are lower: 0.7 and 0.8, respectively (see Figure S2). These latter systems also present the lowest π-NOBD populations in the S_0 state.

Table S16. $EDDB_H$ of the S_0 and lowest ^{3}nπ* states for group A systems. S_0 values are dissected into σ- and π-components and ^{3}nπ* into σ, π, α, and β. The calculations were done at CAM-B3LYP/6-311+G(d,p) level of theory

	S_0			Lowest vertical ^{3}n,π*						
Compound	EDDB	$EDDB_\pi$	$EDDB_\sigma$	EDDB	$EDDB_\alpha$	$EDDB_\beta$	$EDDB_{\pi\alpha}$	$EDDB_{\pi\beta}$	$EDDB_{\sigma\alpha}$	$EDDB_{\sigma\beta}$
**1**	5.69	5.28	0.41	4.09	1.28	2.81	1.09	2.49	0.19	0.32
**2**	5.51	5.15	0.36	3.80	1.27	2.53	1.06	2.32	0.22	0.21
**3**	5.59	5.13	0.46	4.44	1.68	2.76	1.48	2.60	0.20	0.17
**4**	5.15	4.67	0.48	4.31	1.53	2.78	1.36	2.54	0.17	0.25
**5**	4.45	4.15	0.30	4.12	1.18	2.94	1.01	1.76	0.16	1.19
**6**	5.08	4.80	0.28	4.17	1.47	2.70	1.32	1.99	0.15	0.70

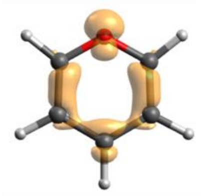

5 **6**

Figure S1. EDDBσ surfaces of systems **5** and **6** with an isocontour of 0.02. For all the other systems the surface was not visible with the present isocontour value.

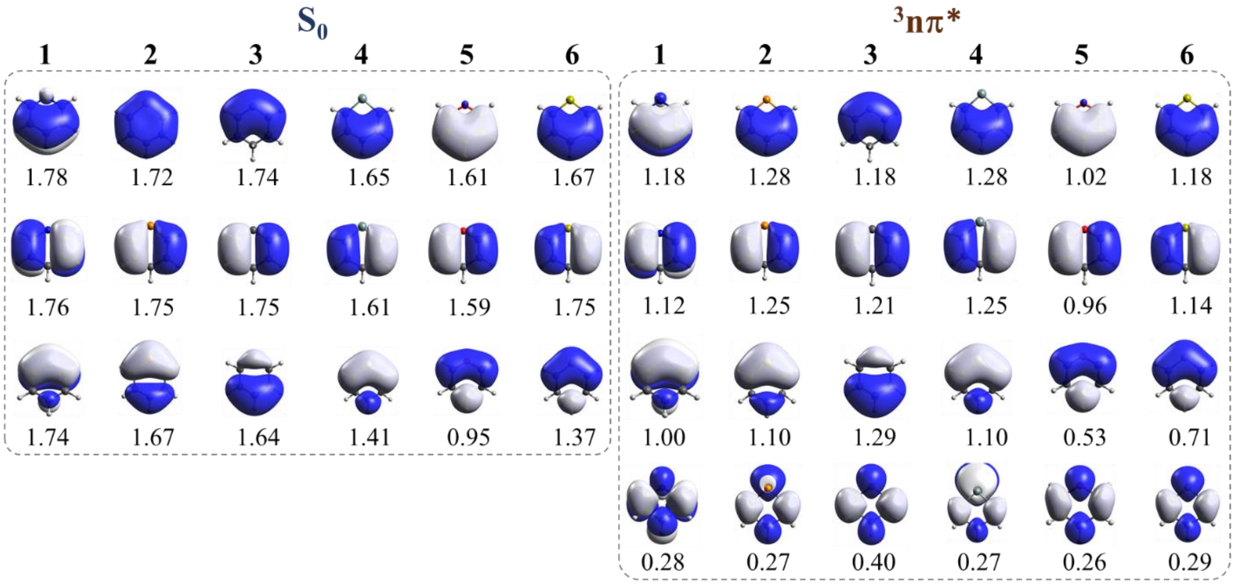

Figure S2. Representation of the π-natural orbitals for bond delocalization (with isocontour 0.02) and its populations in S_0 and ^{3}nπ* states for systems in group A.

For group B, EDDB predicts that all systems with the exception of **14** have β-components in the ^{3}nπ* state that are greater than half of the total S_0 state value (Table S17). The aromatic character is subtle in most of the cases, only **9**, **15** and **16** present slightly higher values of the residual. Although EDDB and MCI results differ in the assignation of the residual character, overall the order of the compounds from more to less aromatic is comparable, with the exception of **14**, for which EDDB in the ^{3}nπ* state gives a β-component of only 42% of that of the total S_0 value.

Similar to what was observed for **5** in group A, group B systems showing considerable residual aromaticity have π_β-values comparable to other molecules in the group but with a larger σ-component. Among them, **15** and **16** are the ones showing more σ-delocalization. These results are in good agreement with the separation into σ/π-contributions of MCI (Table S14).

Table S17. $EDDB_H$ of the S_0 and lowest ^{3}nπ* states for group B systems. S_0 values are dissected into σ- and π-components and ^{3}nπ* into σ, π, α, and β. The calculations have been done at CAM-B3LYP/6-311+G(d,p) level of theory.

	S_0			Lowest vertical ^{3}n,π*						
**Compound**	**EDDB**	**$EDDB_\pi$**	**$EDDB_\sigma$**	**EDDB**	**$EDDB_\alpha$**	**$EDDB_\beta$**	**$EDDB_{\pi\alpha}$**	**$EDDB_{\pi\beta}$**	**$EDDB_{\sigma\alpha}$**	**$EDDB_{\sigma\beta}$**
**7**	5.24	4.75	0.49	4.01	1.32	2.69	1.11	2.38	0.21	0.31
**8**	5.80	5.20	0.60	5.00	1.98	3.02	1.70	2.48	0.28	0.55
**9**	5.74	5.23	0.51	4.82	1.33	3.49	1.09	2.63	0.24	0.86
**10**	5.48	4.95	0.53	4.45	1.50	2.95	1.28	2.41	0.22	0.54
**11**	5.68	5.12	0.56	4.62	1.44	3.18	1.18	2.49	0.25	0.70
**12**	5.69	5.10	0.60	4.51	1.40	3.11	1.13	2.35	0.27	0.76
**13**	5.13	4.68	0.45	3.72	0.90	2.82	0.71	2.39	0.20	0.42
**14**	5.60	4.97	0.64	3.58	1.20	2.38	0.98	2.18	0.22	0.19
**15**	4.80	4.45	0.34	4.61	0.80	3.82	0.62	2.27	0.17	1.55
**16**	4.02	3.64	0.38	4.61	1.58	3.04	1.38	1.72	0.20	1.31
**17**	5.92	5.15	0.77	5.57	2.28	3.29	1.97	2.62	0.32	0.67
**18**	5.86	5.13	0.73	4.83	1.83	3.00	1.53	2.60	0.30	0.39
**19**	5.47	4.92	0.55	4.47	1.48	2.99	1.24	2.46	0.24	0.52
**9H+**	5.44	5.02	0.42	3.90	1.08	2.83	0.90	2.55	0.18	0.27
**19H+**	5.22	4.83	0.39	3.95	1.24	2.72	1.07	2.50	0.16	0.22

2.3. NICS

2.3.1. Group A (Monoheteroaromatics)

Table S18. NICS results of the S_0 and the lowest vertical $^3n,\pi^*$ state of group A, calculated at CAM-B3LYP/6-311+G(d,p) level of theory.

	S_0			Lowest vertical $^3n,\pi^*$		
**Compound**	**NICS(0)$_{zz}$**	**NICS(1)$_{zz}$**	**NICS(1.7)$_{zz}$**	**NICS(0)$_{zz}$**	**NICS(1)$_{zz}$**	**NICS(1.7)$_{zz}$**
**1**	-13.7	-29.1	-21.0	82.7	53.3	25.4
**2**	-12.5	-27.2	-21.7	44.7	26.8	12.6
**3**	-14.5	-28.1	-20.8	280.3	213.4	121.6
**4**	-9.3	-22.6	-19.0	48.9	29.6	15.0
**5**	-7.8	-24.4	-18.1	9.3	14.4	8.4
**6**	-10.2	-26.3	-20.8	18.1	25.9	18.5

2.3.2. Group B (Diheteroaromatics)

Table S19. NICS results of the S_0 and the lowest vertical $^3n,\pi^*$ state of group B, calculated at CAM-B3LYP/6-311+G(d,p) level of theory.

	S_0			Lowest vertical $^3n,\pi^*$		
**Compound**	**NICS(0)$_{zz}$**	**NICS(1)$_{zz}$**	**NICS(1.7)$_{zz}$**	**NICS(0)$_{zz}$**	**NICS(1)$_{zz}$**	**NICS(1.7)$_{zz}$**
**7**	-12.2	-28.8	-20.4	69.1	42.8	19.3
**8**	-11.2	-27.8	-19.8	142.4	96.3	47.9
**9**	-13.1	-28.9	-20.4	48.8	30.3	13.2
**10**	-10.7	-25.8	-20.1	57.9	41.6	21.4
**11**	-10.8	-26.4	-20.7	134.0	96.2	52.0
**12**	-10.6	-26.3	-20.6	46.8	30.0	14.2
**13**	-8.9	-25.8	-18.3	23.4	5.8	0.8
**14**	-13.0	-26.9	-19.6	121.1	82.9	43.3
**15**	-6.9	-24.1	-16.9	-2.2	-4.7	-4.5
**16**	2.5	-17.4	-13.8	30.2	25.9	13.2
**17**	-17.6	-26.5	-18.9	215.2	172.4	106.1
**18**	-15.7	-25.6	-18.5	304.9	241.5	149.5
**19**	-10.4	-24.9	-21.2	40.0	30.1	17.4
**9H+**	-11.5	-28.1	-19.9	40.4	17.8	5.2
**19H+**	-10.2	-24.2	-20.5	41.7	25.7	11.9

2.4. MICD

Within the CTOCD-DZ methodology, also known as the *ipsocentric* approach [36], the magnetically induced current density is calculated based on the first order perturbed wave function, which has contributions from the linear momentum ($\hat{p}$) and angular-momentum ($\hat{l}$) perturbation operators:

$$\Psi_n^{(1)}(r) = -\frac{1}{2}\left[\sum_{p>\frac{N}{2}} \Psi_p(r)\frac{\langle\Psi_p|\hat{l}(0)|\Psi_n\rangle}{\varepsilon_p - \varepsilon_n}\right]\cdot B + \frac{1}{2}\left[d \times \sum_{p>\frac{N}{2}} \Psi_p(r)\frac{\langle\Psi_p|\hat{p}|\Psi_n\rangle}{\varepsilon_p - \varepsilon_n}\right]\cdot B = \Psi_n^p(r) + \Psi_n^d(r)$$

where $\Psi_n^{(1)}$ is the first-order correction of an occupied molecular orbital Ψ_n, d is the origin of the vector potential that gives rise to the magnetic field B and the summations go over all unoccupied orbitals. The paratropic current comes from Ψ_n^p orbital corrections, and its intensity can be rationalized through the acceptability of rotational occupied-to-virtual orbital transitions. On the other hand, the diatropic current arises from Ψ_n^d, and its intensity is determined by the acceptability of translational occupied-to-virtual orbital transitions. The contribution of the given virtual orbital transition is more relevant as the energy gap between the occupied and unoccupied orbitals becomes smaller. In addition, the relevance of the occupied-unoccupied transition is also determined by the values of respective linear/angular momentum matrix elements: $\hat{p}_x$ and $\hat{p}_y$ for diatropic contributions and $\hat{l}_z$ for paratropic contributions (assuming that a molecule of interest lies in the *xy*-plane). Thereafter, the magnetic responses of a molecule can be qualitatively interpreted by combining symmetry-, energy- and spin-based selection rules.

All molecules from Group A belong to the C_{2v} point group, and their frontier molecular orbitals in the S_0 and nπ* states can be described by a simplified model displayed in Figure 6E. It should be mentioned here, that for the present analysis the order of unoccupied b_1 and a_2 molecular orbitals is irrelevant. In the S_0 state π-electrons give diatropic currents due to translational transitions between the occupied b_1 and a_2 levels and unoccupied b_1 and a_2 molecular orbitals. It should be noted that the transitions b_1 to a_2 and a_2 to b_1 are also rotationally allowed, but the calculated angular momentum matrix elements showed that these transitions negligibly contribute to paratropic currents. Thus, the occupied b_1 and a_2 π–molecular orbitals in the S_0 state give diatropic currents. For the ^{3}nπ* state α and β electrons are considered separately, and for π_β electrons completely analogous transitions are found as those in the S_0 state. On the other hand, according to the energy-based selection rule the π_α-electron current density will be dominantly determined by the b_1 orbital which was unoccupied in the S_0 state. The transition b_1 to a_2 within the π_α stack is both translationally and rotationally allowed since the symmetry product $b_1 \times a_2 = b_2$ contains the symmetry of the in-plane translations, as well as the symmetry of the in-plane rotation. However, for all Group A molecules it was found that the transition from the highest occupied b_1 level to the empty a_2 solely contribute to paratropic currents, which become very strong due to the small energy gap between these two levels.

The qualitative predictions of this simple model were confirmed in our calculations. Table S20 provides the π-electron ring current strengths for selected molecules in the S_0 and ^{3}nπ* states. For the triplet nπ* state, the π-electron ring current strengths were decomposed into α- and β-spin components, and in addition, π_α-electron ring current strengths were further calculated by

separating the contribution of the π_α-HSOMO level. Table S20 contains the π_α HOMO-LUMO energy gap, since this quantity is related to the intensity of the π_α-HSOMO current density contribution.

It was found that for all molecules from Group A the π-electrons sustain diatropic ring currents in the S_0 state. Similarly, π_β-component in the $^3n\pi^*$ states exhibit diatropic current density contribution. In addition, the found strong paratropic contribution of π_α electrons completely come from the π_α-HSOMO (highest singly occupied orbital). The contribution of the π_α-orbitals from which the π_α-HSOMO contribution was excluded is always diatropic, but significantly weaker than the corresponding contribution of π_α electrons in the S_0 state, which can also be deduced from Figure 6E. The intensity π_α-HSOMO current density contribution was found to be proportional to the energy gap between this orbital and lowest unoccupied π_α orbital (Table S20).

Full-scale plots of the magnetically induced current density maps are found in Section 4.

Table S20. π-electron ring current strengths (in nA T^{-1}) calculated as the average of all bonds in the given ring. For the vertical $^3n\pi^*$ state the total π_α–electron current strength was decomposed into the π_α-HSOMO contribution and the contribution of all other π_α orbitals (total - HSOMO). $\Delta E_{H\text{-}L}$ (in Hartrees) is the HOMO-LUMO energy gap for π_α orbitals.

Grp.	Comp.	**S_0**				**$^3n,\pi^*$**								
							π_α							
		π	$\pi_{\alpha/\beta}$	total	$total_{\alpha/\beta}$	π	total	HSOMO	total - HSOMO	$\Delta E_{H\text{-}L}$	π_β	total	$total_\alpha$	$total_\beta$
**A**	**1**	11.1	5.5	11.2	5.6	-19.8	-24.9	-27.3	2.4	0.19	5.1	-19.9	-25.5	5.6
	**2**	11.1	5.6	11.3	5.7	-8.6	-14.1	-16.7	2.7	0.19	5.4	-9.8	-14.4	4.6
	**3**	11.4	5.7	11.2	5.6	-81.6	-87.3	-90.3	3.0	0.14	5.8	-83.5	-89.4	5.9
	**4**	10.1	5.0	10.0	5.0	-10.2	-15.5	-18.3	2.8	0.16	5.2	-11.2	-16.0	4.8
	**5**	9.0	4.5	9.3	4.6	-5.1	-8.8	-10.9	2.0	0.23	3.7	-5.1	-9.0	3.8
	**6**	10.3	5.1	10.5	5.3	-4.6	-8.8	-11.1	2.3	0.23	4.3	-10.8	-9.0	-1.8
**B**	**7**	10.8	5.4	10.7	5.3	-18.5	-21.4	-23.6	2.2	0.20	2.9	-15.8	-21.9	6.1
	**8**	10.5	5.3	10.4	5.2	-35.0	-39.8	-41.9	2.1	0.18	4.8	-36.2	-40.8	4.6
	**9**	10.9	5.4	10.8	5.4	-13.2	-18.8	-21.2	2.4	0.21	5.5	-11.0	-19.3	8.3

3. Further Analysis

3.1. Energies

3.1.1. Group A (Monoheteroaromatics)

Table S21. Absolute energies of the S_0 and the lowest vertical ^{3}n,π* state, as well as the vertical excitation energies of the two lowest ^{3}n,π* and ^{3}π,π* states, respectively, of group A. The latter implies that there, for compounds **5** and **6**, may be more ^{3}π,π* states of lower energy than that of the lowest reported ^{3}n,π* state. The lowest ^{3}n,π* states are all of the same type (type I, Figure 3B). The energies were calculated at CAM-B3LYP/6-311+G(d,p) level of theory.

	Absolute Energy [Ha]		Vertical Excitation Energy [eV]			
**Compound**	**S_0**	**Lowest ^{3}n,π***	**^{3}n,π***		**^{3}π,π***	
**1**	-248.21310812	-248.05501668	4.30	5.26	4.74	4.80
**2**	-534.82546526	-534.67031797	4.22	5.50	3.28	4.51
**3**	-231.51950237	-231.42400185	2.60	2.80	4.34	5.30
**4**	-482.97083050	-482.86515859	2.88	3.82	2.90	4.99
**5**	-268.40403821	-268.19124960	5.79	7.84	3.64	4.62
**6**	-591.40141295	-591.19896362	5.51	7.39	3.63	3.74

Table S22. Absolute energies of the S_0 and the lowest vertical ^{3}n,π* state, as well as the vertical excitation energy of the latter, of group A. The energies were calculated at CCSD/6-311+G(d,p) level of theory and, thus, also the T_1 diagnostics values are given. The thresholds for T_1 diagnostics values are 0.02 and 0.044 for closed-shell and open-shell systems, respectively [46-48].

	Absolute Energy [Ha]		Vertical Excitation Energy [eV]	T_1 diagnostics	
**Compound**	**S_0**	**Lowest ^{3}n,π***	**Lowest ^{3}n,π***	**S_0**	**Lowest ^{3}n,π***
**1**	-247.6443945	-247.4803812	4.46	0.0125	0.0397
**2**	-533.8710306	-533.7110005	4.35	0.0126	0.0403
**3**	-230.9705111	-230.8723264	2.67	0.0144	0.0206
**4**	-482.0342769	-481.9273992	2.91	0.0142	0.029
**5**	-267.8095876	-267.5827810	6.17	0.0171	0.0596
**6**	-590.4248560	-590.2154455	5.70	0.0160	0.0453

Table S23. Absolute energies of the S_0 and the lowest vertical ^{3}n,π* state, as well as the vertical excitation energy of the latter, of group A. The energies were calculated at BD/6-311+G(d,p)//CCSD/6-311+G(d,p) level of theory.

	Absolute Energy [Ha]		Vertical Excitation Energy [eV]
**Compound**	**S_0**	**Lowest ^{3}n,π***	**Lowest ^{3}n,π***
**1**	-247.7648827	-247.6007225	4.47
**3**	-231.0902086	-230.9920203	2.67
**5**	-267.9300934	-267.7031386	6.18
**6**	-590.6451296	-590.4351539	5.71

3.1.2. Group B (Diheteroaromatics)

Table S24. Absolute energies of the S_0 and the lowest vertical ^{3}n,π* state, as well as the vertical excitation energies of the (where possible) two lowest ^{3}n,π* and ^{3}π,π* states, respectively, of group B. The latter implies that there, for compounds **10** and **11**, may be more ^{3}π,π* states of lower energy than that of the lowest reported ^{3}n,π* state. The lowest ^{3}n,π* states are all of the same type (Figure 3B), except in the case of compounds **17** and **18**. The energies were calculated at CAM-B3LYP/6-311+G(d,p) level of theory.

	Absolute Energy [Ha]		Vertical Excitation Energy [eV]			
**Compound**	**S_0**	**Lowest ^{3}n,π***	**^{3}n,π***		**^{3}π,π***	
**7**	-264.22090425	-264.11234275	2.95	4.04	4.55	4.78
**8**	-264.25890626	-264.10726270	4.13	4.67	4.91	5.06
**9**	-264.25165650	-264.11989603	3.59	4.92	4.3	5.12
**10**	-550.87247447	-550.74980681	3.34	4.25	3.27	-
**11**	-550.86309724	-550.73096481	3.60	-	3.26	-
**12**	-550.86657157	-550.73562227	3.56	4.81	3.51	4.19
**13**	-284.41283529	-284.32321890	2.44	5.08	3.05	5.01
**14**	-247.57907025	-247.49138447	2.39	3.03	4.59	5.05
**15**	-304.33266726	-304.19173759	3.83	7.08	1.93	6.40
**16**	-304.36280903	-304.15860722	5.56	6.68	3.44	7.40
**17**	-230.71927515	-230.60691508	3.06	3.25	3.98	4.20
**18**	-230.71288922	-230.61189920	2.75	2.87	4.08	4.22
**19**	-837.47725942	-837.33172940	3.96	5.22	2.53	4.24
**$9H^+$**	-264.59475763	-264.48536560	2.98	4.96	3.61	5.08
**$19H^+$**	-837.78903599	-837.66466394	3.38	4.78	2.62	4.18

Table S25. Absolute energies of the S_0 and the lowest vertical ^{3}n,π* state, as well as the vertical excitation energy of the latter, of group B. The energies were calculated at CCSD/6-311+G(d,p) level of theory and, thus, also the T_1 diagnostics values are given. Compound **19H⁺** is modestly non-planar in its S_0 geometry, with the atoms of the P-H unit being slightly out-of-plane. The thresholds for T_1 diagnostics values are 0.02 and 0.044 for closed-shell and open-shell systems, respectively [46-48].

	**Absolute Energy [Ha]**		**Vertical Excitation Energy [eV]**	**T_1 diagnostics**	
**Compound**	**S_0**	**Lowest ^{3}n,π***	**Lowest ^{3}n,π***	**S_0**	**Lowest ^{3}n,π***
**7**	-263.6287600	-263.5053121	3.36	0.0123	0.0586
**8**	-263.6663730	-263.5046188	4.40	0.0153	0.0565
**9**	-263.6590993	-263.5155166	3.91	0.0121	0.0576
**10**	-549.8917194	-549.7619442	3.53	0.0142	0.0509
**11**	-549.8843360	-549.7436484	3.83	0.0158	0.0549
**12**	-549.8878308	-549.7497556	3.76	0.0140	0.0481
**13**	-283.7953639	-283.7020366	2.54	0.0157	0.0381
**14**	-247.0048196	-246.9153050	2.44	0.0161	0.0265
**15**	-303.6934073	-303.5363363	4.27	0.0130	0.0163
**16**	-303.7248442	-303.5054285	5.97	0.0218	0.0416
**17**	-230.1675042	-230.0499342	3.20	0.0213	0.0554
**18**	-230.1618438	-230.0553197	2.90	0.0233	0.0449
**19**	-836.1124892	-835.9618365	4.10	0.0139	0.0194
**9H⁺**	-264.0062542	-263.8910039	3.14	0.0122	0.0290
**19H⁺**	-836.4292304	-836.3074228	3.31	0.0140	0.0317

Table S26. Absolute energies of the S_0 and the lowest vertical ^{3}n,π* state, as well as the vertical excitation energy of the latter, of group B. The energies were calculated at BD/6-311+G(d,p)//CCSD/6-311+G(d,p) level of theory.

	**Absolute Energy [Ha]**		**Vertical Excitation Energy [eV]**
**Compound**	**S_0**	**Lowest ^{3}n,π***	**Lowest ^{3}n,π***
**7**	-263.7490387	-263.6281586	3.29
**8**	-263.7867607	-263.6259552	4.38
**9**	-263.7795549	-263.6372552	3.87
**10**	-550.1158232	-549.9864452	3.52
**11**	-550.1083479	-549.9679636	3.82
**12**	-550.1119876	-549.9726844	3.79
**15**	-303.8138766	-303.6560860	4.29
**16**	-303.8447130	-303.6237538	6.01
**17**	-230.2863961	-230.1673057	3.24
**18**	-230.2804219	-230.1735967	2.91

3.2. TD-DFT results

3.2.1. Group A (Monoheteroaromatics)

Table S27. Energies of the lowest excited states of states of group A, calculated at TD-CAM-B3LYP/6-311+G(d,p) level of theory.

Compound	State	Character	Type	Symmetry	Excitation Energy [eV]
**1**	T_1	π,π^*		A_1	3.79
	T_2	n,π^*	I	B_1	4.28
	S_1	n,π^*	I	B_1	5.06
	S_2	n,π^*	II	A_2	5.44
	S_3	π,π^*		B_2	5.63
**2**	T_1	π,π^*		A_1	3.79
	T_2	n,π^*	I	B_1	4.28
	S_1	π,π^*		B_2	4.91
	S_2	n,π^*	I	B_1	5.14
**3**	T_1	n,π^*	I (mainly)	B_1	2.61
	T_2	n,π^*	II (mainly)	A_2	2.97
	T_3	n,Ry		A_1	3.07
	S_1	n,π^*	II	A_2	3.07
	S_2	n,Ry		A_1	3.13
	S_3	n,Ry		B_2	3.39
	S_4	n,π^*	I	B_1	3.49
**4**	T_1	π,π^*		A_1	2.66
	T_2	n,π^*	I	B_1	2.73
	S_1	Mix: n,π^*/π,Ry	(I)	B_1	3.73
	S_2	Mix: n,π^*/π,Ry	I	B_1	3.78
	S_3	Mix: π,π^*/n,Ry		A_1	4.02
**5**	T_1	π,π^*		B_2	3.39
	T_2	π,π^*		A_1	4.31
	T_3	π,π^*		A_1	4.67
	T_4	n,π^*	I	B_1	5.73
	S_1	π,π^*		B_2	5.33
	S_2	n,π^*	I	B_1	6.20
**6**	T_1	π,π^*		A_1	3.35
	T_2	π,π^*		B_2	3.40
	T_3	π,π^*		A_1	4.50
	T_4	n,π^*	I	B_1	5.48
	S_1	π,π^*		B_2	5.02
	S_2	π,π^*		A_1	5.61
	S_3	n,π^*	I	B_1	6.07

3.2.2. Group B (Diheteroaromatics)

Table S28. Energies of the lowest excited states of states of some of the compounds in group B, calculated at TD-CAM-B3LYP/6-311+G(d,p) level of theory.

Compound	State	Character	Type	Symmetry	Excitation Energy [eV]	Absolute Energy [Ha]
**7**	T_1	n,π*	I	B_1	2.87	-264,115615998
	T_2	π,π*		B_2	3.79	-
	T_3	n,π*	II	A_2	3.93	-
	S_1	n,π*	I	B_1	3.78	-264,082020636
	S_2	n,π*	II	A_2	4.41	-
	S_3	π,π*		A_1	5.81	-
**8**	T_1	n,π*	I	B_1	3.96	-264,113223244
	T_2	π,π*		A_1	4.02	-
	T_3	n,π*	II	A_2	4.53	-
	S_1	n,π*	I	B_1	4.56	-264,09133792
	S_2	n,π*	II	A_2	4.91	-
	S_3	π,π*		B_2	5.91	-
**9**	T_1	n,π*	I	B_{3u}	3.39	-264,127122093
	T_2	π,π*		B_{1u}	3.83	-
	T_4	n,π*	II	A_u	4.82	-
	T_5	n,π*		B_{2g}	4.84	-
	T_8	n,π*		B_{1g}	6.73	-
	S_1	n,π*	I	B_{3u}	4.17	-264,098238875
	S_2	n,π*	II	A_u	4.96	-
	S_3	π,π*		B_{2u}	5.50	-
	S_4	n,π*		B_{2g}	5.87	-
	S_6	n,π*		B_{1g}	6.87	-

3.3. Electron Distribution

In this section, the electron occupation of the out-of-plane atomic p-orbitals of the heterocycles are presented.

3.3.1. Group A (Monoheteroaromatics)

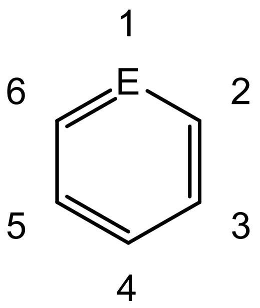

Figure S3. Atom numbering used for the NPA data of group A.

Table S29. Electron distribution of the S_0 and the lowest vertical ^{3}n,π* state of compound **1**, calculated at CAM-B3LYP/6-311+G(d,p) level of theory. The difference between the largest and smallest value is also displayed.

Compound 1	S_0	S_0(α/β)	^{3}n,π*	^{3}n,π*(α)	^{3}n,π*(β)
N1	1.16059	0.58030	1.57107	0.93102	0.64005
C2	0.92162	0.46081	1.08960	0.64999	0.43961
C3	1.01486	0.50743	1.02576	0.47393	0.55183
C4	0.95001	0.47501	1.14875	0.77981	0.36894
C5	1.01486	0.50743	1.02576	0.47393	0.55183
C6	0.92162	0.46081	1.08960	0.64999	0.43961
Max-min	0.23897	0.11949	0.54531	0.45709	0.27111

Table S30. Electron distribution of the S_0 and the lowest vertical ^{3}n,π* state of compound **2**, calculated at CAM-B3LYP/6-311+G(d,p) level of theory. The difference between the largest and smallest value is also displayed.

Compound 2	S_0	S_0(α/β)	^{3}n,π*	^{3}n,π*(α)	^{3}n,π*(β)
P1	0.90749	0.45375	1.47847	0.95191	0.52655
C2	1.07600	0.53800	1.20563	0.71090	0.49473
C3	0.96613	0.48307	0.96620	0.43193	0.53427
C4	0.98723	0.49362	1.12729	0.72328	0.40401
C5	0.96613	0.48307	0.96620	0.43193	0.53427
C6	1.07600	0.53800	1.20563	0.71090	0.49473
Max-min	0.16851	0.084255	0.51227	0.51998	0.13026

Table S31. Electron distribution of the S_0 and the lowest vertical ^{3}n,π* state of compound **3**, calculated at CAM-B3LYP/6-311+G(d,p) level of theory. The difference between the largest and smallest value is also displayed.

**Compound 3**	**S_0**	**S_0(α/β)**	**^{3}n,π***	**^{3}n,π*(α)**	**^{3}n,π*(β)**
**C1**	0.71525	0.35763	1.22386	0.83207	0.39179
**C2**	1.03989	0.51995	1.10964	0.56438	0.54525
**C3**	1.04282	0.52141	1.08193	0.56293	0.51901
**C4**	1.09609	0.54805	1.28439	0.81607	0.46832
**C5**	1.04282	0.52141	1.08193	0.56293	0.51901
**C6**	1.03989	0.51995	1.10964	0.56438	0.54525
**Max-min**	0.38084	0.19042	0.20246	0.26914	0.15346

Table S32. Electron distribution of the S_0 and the lowest vertical ^{3}n,π* state of compound **4**, calculated at CAM-B3LYP/6-311+G(d,p) level of theory. The difference between the largest and smallest value is also displayed.

**Compound 4**	**S_0**	**S_0(α/β)**	**^{3}n,π***	**^{3}n,π*(α)**	**^{3}n,π*(β)**
**Si1**	0.52424	0.26212	1.20429	0.91267	0.29162
**C2**	1.15452	0.57726	1.22289	0.65259	0.5703
**C3**	1.00161	0.50081	1.00282	0.48526	0.51756
**C4**	1.13551	0.56776	1.23524	0.71763	0.51761
**C5**	1.00161	0.50081	1.00282	0.48526	0.51756
**C6**	1.15452	0.57726	1.22289	0.65259	0.57030
**Max-min**	0.63028	0.31514	0.23242	0.42741	0.27868

Table S33. Electron distribution of the S_0 and the lowest vertical ^{3}n,π* state of compound **5**, calculated at CAM-B3LYP/6-311+G(d,p) level of theory. The difference between the largest and smallest value is also displayed.

**Compound 5**	**S_0**	**S_0(α/β)**	**^{3}n,π***	**^{3}n,π*(α)**	**^{3}n,π*(β)**
**O1**	1.59732	0.79866	1.79613	0.97078	0.82536
**C2**	0.82511	0.41256	1.06310	0.69936	0.36373
**C3**	0.97917	0.48959	0.99225	0.42279	0.56946
**C4**	0.77956	0.38978	1.06506	0.76349	0.30157
**C5**	0.97917	0.48959	0.99225	0.42279	0.56946
**C6**	0.82511	0.41256	1.06310	0.69936	0.36373
**Max-min**	0.81776	0.40888	0.80388	0.54799	0.52379

Table S34. Electron distribution of the S_0 and the lowest vertical ^{3}n,π* state of compound **6**, calculated at CAM-B3LYP/6-311+G(d,p) level of theory. The difference between the largest and smallest value is also displayed.

Compound 6	S_0	$S_0(\alpha/\beta)$	^{3}n,π*	^{3}n,π*(α)	^{3}n,π*(β)
**S1**	1.36679	0.68340	1.72530	0.97205	0.75324
**C2**	0.96304	0.48152	1.16578	0.74845	0.41734
**C3**	0.93307	0.46654	0.93868	0.39197	0.54671
**C4**	0.81985	0.40993	1.02877	0.72077	0.30801
**C5**	0.93307	0.46654	0.93868	0.39197	0.54671
**C6**	0.96304	0.48152	1.16578	0.74845	0.41734
**Max-min**	0.54694	0.27347	0.78662	0.58008	0.44523

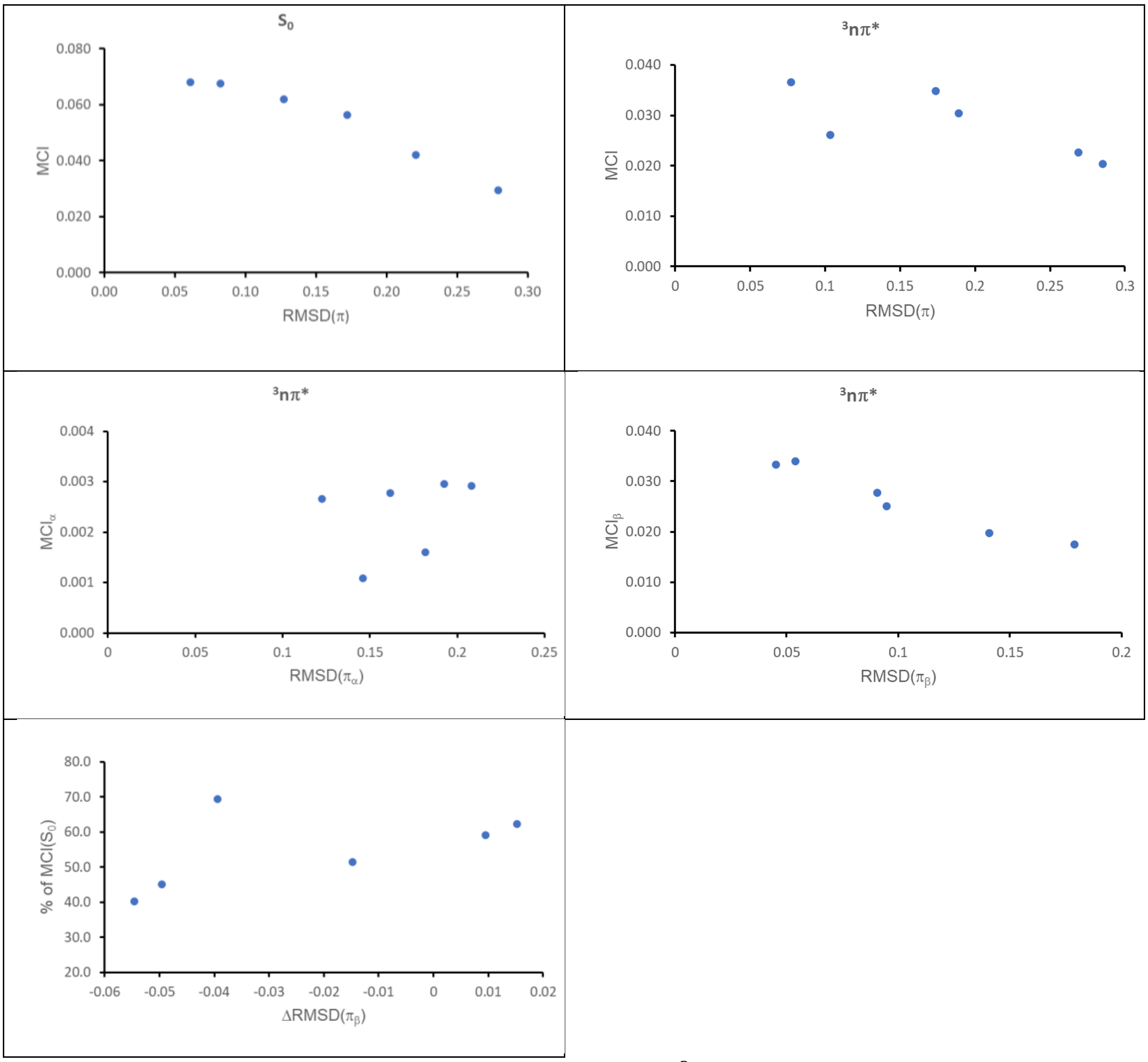

Figure S4. MCI results of the S_0 and the lowest vertical ^{3}n,π* state of the compounds of Group A versus the root mean square deviation of natural atomic p-orbital occupations (RMSD(π)), and % of MCI (S_0) versus the difference in RMSD(π_β) between the S_0 and the lowest vertical ^{3}n,π* states (ΔRMSD(π_β)).

3.3.2. Group B (Diheteroaromatics)

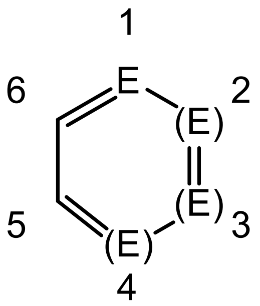

Figure S5. Atom numbering used for the NPA data of group B.

Table S35. Electron distribution of the S_0 and the lowest vertical ^{3}n,π* state of compound **7**, calculated at CAM-B3LYP/6-311+G(d,p) level of theory. The difference between the largest and smallest value is also displayed.

Compound 7	S_0	S_0(α/β)	^{3}n,π*	^{3}n,π*(α)	^{3}n,π*(β)
**N1**	1.09837	0.54919	1.43456	0.88079	0.55377
**N2**	1.09837	0.54919	1.43456	0.88079	0.55377
**C3**	0.93055	0.46528	0.94972	0.45250	0.49722
**C4**	0.96280	0.48140	1.09517	0.65033	0.44484
**C5**	0.96280	0.48140	1.09517	0.65033	0.44484
**C6**	0.93055	0.46528	0.94972	0.45250	0.49722
**Max-min**	0.16782	0.08391	0.48484	0.42829	0.10893

Table S36. Electron distribution of the S_0 and the lowest vertical ^{3}n,π* state of compound **8**, calculated at CAM-B3LYP/6-311+G(d,p) level of theory. The difference between the largest and smallest value is also displayed.

Compound 8	S_0	S_0(α/β)	^{3}n,π*	^{3}n,π*(α)	^{3}n,π*(β)
**N1**	1.17461	0.58731	1.42398	0.80013	0.62385
**C2**	0.85093	0.42547	0.84013	0.38673	0.45340
**N3**	1.17461	0.58731	1.42398	0.80013	0.62385
**C4**	0.87614	0.43807	1.13183	0.76614	0.36569
**C5**	1.02963	0.51482	1.00015	0.44132	0.55883
**C6**	0.87614	0.43807	1.13183	0.76614	0.36569
**Max-min**	0.32368	0.16184	0.58385	0.41340	0.25816

Table S37. Electron distribution of the S_0 and the lowest vertical $^3n,\pi^*$ state of compound **9**, calculated at CAM-B3LYP/6-311+G(d,p) level of theory. The difference between the largest and smallest value is also displayed.

Compound 9	S_0	$S_0(\alpha/\beta)$	$^3n,\pi^*$	$^3n,\pi^*(\alpha)$	$^3n,\pi^*(\beta)$
**N1**	1.11672	0.55836	1.40939	0.88183	0.52756
**C2**	0.93680	0.46840	1.03403	0.54999	0.48404
**C3**	0.93680	0.46840	1.03403	0.54999	0.48404
**N4**	1.11672	0.55836	1.40939	0.88183	0.52756
**C5**	0.93680	0.46840	1.03403	0.54999	0.48404
**C6**	0.93680	0.46840	1.03403	0.54999	0.48404
**Max-min**	0.17992	0.08996	0.37536	0.33184	0.04352

Table S38. Electron distribution of the S_0 and the lowest vertical $^3n,\pi^*$ state of compound **10**, calculated at CAM-B3LYP/6-311+G(d,p) level of theory. The difference between the largest and smallest value is also displayed.

Compound 10	S_0	$S_0(\alpha/\beta)$	$^3n,\pi^*$	$^3n,\pi^*(\alpha)$	$^3n,\pi^*(\beta)$
**N1**	1.25127	0.62564	1.57698	0.90569	0.67129
**P2**	0.81534	0.40767	1.19066	0.86132	0.32934
**C3**	1.08950	0.54475	1.10369	0.50129	0.60240
**C4**	0.92611	0.46306	1.01990	0.61490	0.40500
**C5**	1.01127	0.50564	1.12272	0.59925	0.52346
**C6**	0.88290	0.44145	0.93790	0.48162	0.45627
**Max-min**	0.43593	0.217965	0.63908	0.42407	0.34195

Table S39. Electron distribution of the S_0 and the lowest vertical $^3n,\pi^*$ state of compound **11**, calculated at CAM-B3LYP/6-311+G(d,p) level of theory. The difference between the largest and smallest value is also displayed.

Compound 11	S_0	$S_0(\alpha/\beta)$	$^3n,\pi^*$	$^3n,\pi^*(\alpha)$	$^3n,\pi^*(\beta)$
**N1**	1.14136	0.57068	1.48270	0.85733	0.62537
**C2**	0.98518	0.49259	1.04693	0.51209	0.53484
**P3**	0.92576	0.46288	1.09066	0.60882	0.48184
**C4**	1.02839	0.51420	1.22641	0.82534	0.40107
**C5**	0.98534	0.49267	0.97321	0.44078	0.53243
**C6**	0.91024	0.45512	1.12432	0.71142	0.41290
**Max-min**	0.23112	0.11556	0.50949	0.41655	0.22430

Table S40. Electron distribution of the S_0 and the lowest vertical $^3n,\pi^*$ state of compound **12**, calculated at CAM-B3LYP/6-311+G(d,p) level of theory. The difference between the largest and smallest value is also displayed.

Compound 12	S_0	$S_0(\alpha/\beta)$	$^3n,\pi^*$	$^3n,\pi^*(\alpha)$	$^3n,\pi^*(\beta)$
**N1**	1.16179	0.58090	1.51709	0.87632	0.64077
**C2**	0.89016	0.44508	0.99885	0.54608	0.45277
**C3**	1.10178	0.55089	1.13775	0.56018	0.57757
**P4**	0.83229	0.41615	1.16651	0.87896	0.28755
**C5**	1.10178	0.55089	1.13775	0.56018	0.57757
**C6**	0.89016	0.44508	0.99885	0.54608	0.45277
**Max-min**	0.32950	0.16475	0.51824	0.33288	0.35322

Table S41. Electron distribution of the S_0 and the lowest vertical $^3n,\pi^*$ state of compound **13**, calculated at CAM-B3LYP/6-311+G(d,p) level of theory. The difference between the largest and smallest value is also displayed.

Compound 13	S_0	$S_0(\alpha/\beta)$	$^3n,\pi^*$	$^3n,\pi^*(\alpha)$	$^3n,\pi^*(\beta)$
**N1**	0.98761	0.49381	1.4159	0.88745	0.52845
**C2**	0.89617	0.44809	0.99040	0.49398	0.49642
**C3**	0.82159	0.41080	0.95005	0.58059	0.36946
**O4**	1.55881	0.77941	1.67209	0.94046	0.73163
**C5**	0.82159	0.41080	0.95005	0.58059	0.36946
**C6**	0.89617	0.44809	0.99040	0.49398	0.49642
**Max-min**	0.73722	0.36861	0.72204	0.44648	0.36217

Table S42. Electron distribution of the S_0 and the lowest vertical $^3n,\pi^*$ state of compound **14**, calculated at CAM-B3LYP/6-311+G(d,p) level of theory. The difference between the largest and smallest value is also displayed.

Compound 14	S_0	$S_0(\alpha/\beta)$	$^3n,\pi^*$	$^3n,\pi^*(\alpha)$	$^3n,\pi^*(\beta)$
**N1**	1.24116	0.62058	1.3937	0.85166	0.54204
**C2**	0.97406	0.48703	1.04203	0.56191	0.48012
**C3**	1.06063	0.53032	1.12270	0.55757	0.56513
**C4**	0.66719	0.33360	1.18959	0.83363	0.35595
**C5**	1.06063	0.53032	1.12270	0.55757	0.56513
**C6**	0.97406	0.48703	1.04203	0.56191	0.48012
**Max-min**	0.57397	0.286985	0.35167	0.29409	0.20918

Table S43. Electron distribution of the S_0 and the lowest vertical $^3n,\pi^*$ state of compound **15**, calculated at CAM-B3LYP/6-311+G(d,p) level of theory. The difference between the largest and smallest value is also displayed.

Compound 15	S_0	$S_0(\alpha/\beta)$	$^3n,\pi^*$	$^3n,\pi^*(\alpha)$	$^3n,\pi^*(\beta)$
**O1**	1.48610	0.74305	1.67101	0.93756	0.73345
**C2**	0.75215	0.37608	0.90761	0.52645	0.38117
**C3**	0.75215	0.37608	0.90761	0.52645	0.38117
**O4**	1.48610	0.74305	1.67101	0.93756	0.73345
**C5**	0.75215	0.37608	0.90761	0.52645	0.38117
**C6**	0.75215	0.37608	0.90761	0.52645	0.38117
**Max-min**	0.73395	0.366975	0.76340	0.41111	0.35228

Table S44. Electron distribution of the S_0 and the lowest vertical $^3n,\pi^*$ state of compound **16**, calculated at CAM-B3LYP/6-311+G(d,p) level of theory. The difference between the largest and smallest value is also displayed.

Compound 16	S_0	$S_0(\alpha/\beta)$	$^3n,\pi^*$	$^3n,\pi^*(\alpha)$	$^3n,\pi^*(\beta)$
**O1**	1.57987	0.78994	1.66871	0.86318	0.80553
**C2**	0.59121	0.29561	0.58062	0.28076	0.29986
**O3**	1.57987	0.78994	1.66871	0.86318	0.80553
**C4**	0.64451	0.32226	1.05430	0.79634	0.25797
**C5**	0.94159	0.47080	0.94629	0.38189	0.56440
**C6**	0.64451	0.32226	1.05430	0.79634	0.25797
**Max-min**	0.98866	0.49433	1.08809	0.58242	0.54756

Table S45. Electron distribution of the S_0 and the lowest vertical $^3n,\pi^*$ state of compound **17**, calculated at CAM-B3LYP/6-311+G(d,p) level of theory. The difference between the largest and smallest value is also displayed.

Compound 17	S_0	$S_0(\alpha/\beta)$	$^3n,\pi^*$	$^3n,\pi^*(\alpha)$	$^3n,\pi^*(\beta)$
**C1**	0.82864	0.41432	1.15597	0.76425	0.39172
**C2**	1.07724	0.53862	1.11525	0.56553	0.54973
**C3**	1.07724	0.53862	1.11525	0.56553	0.54973
**C4**	0.82864	0.41432	1.15597	0.76425	0.39172
**C5**	1.07724	0.53862	1.11525	0.56553	0.54973
**C6**	1.07724	0.53862	1.11525	0.56553	0.54973
**Max-min**	0.24860	0.12430	0.04072	0.19872	0.15801

Table S46. Electron distribution of the S_0 and the lowest vertical ^{3}n,π* state of compound **18**, calculated at CAM-B3LYP/6-311+G(d,p) level of theory. The difference between the largest and smallest value is also displayed.

Compound 18	S_0	S_0(α/β)	^{3}n,π*	^{3}n,π*(α)	^{3}n,π*(β)
**C1**	0.78346	0.39173	0.92010	0.48412	0.43598
**C2**	1.07460	0.53730	1.28593	0.77406	0.51187
**C3**	0.78346	0.39173	0.92010	0.48412	0.43598
**C4**	1.11867	0.55934	1.15788	0.60459	0.55330
**C5**	1.08359	0.54180	1.29948	0.80486	0.49462
**C6**	1.11867	0.55934	1.15788	0.60459	0.55330
**Max-min**	0.33521	0.167605	0.37938	0.32074	0.11732

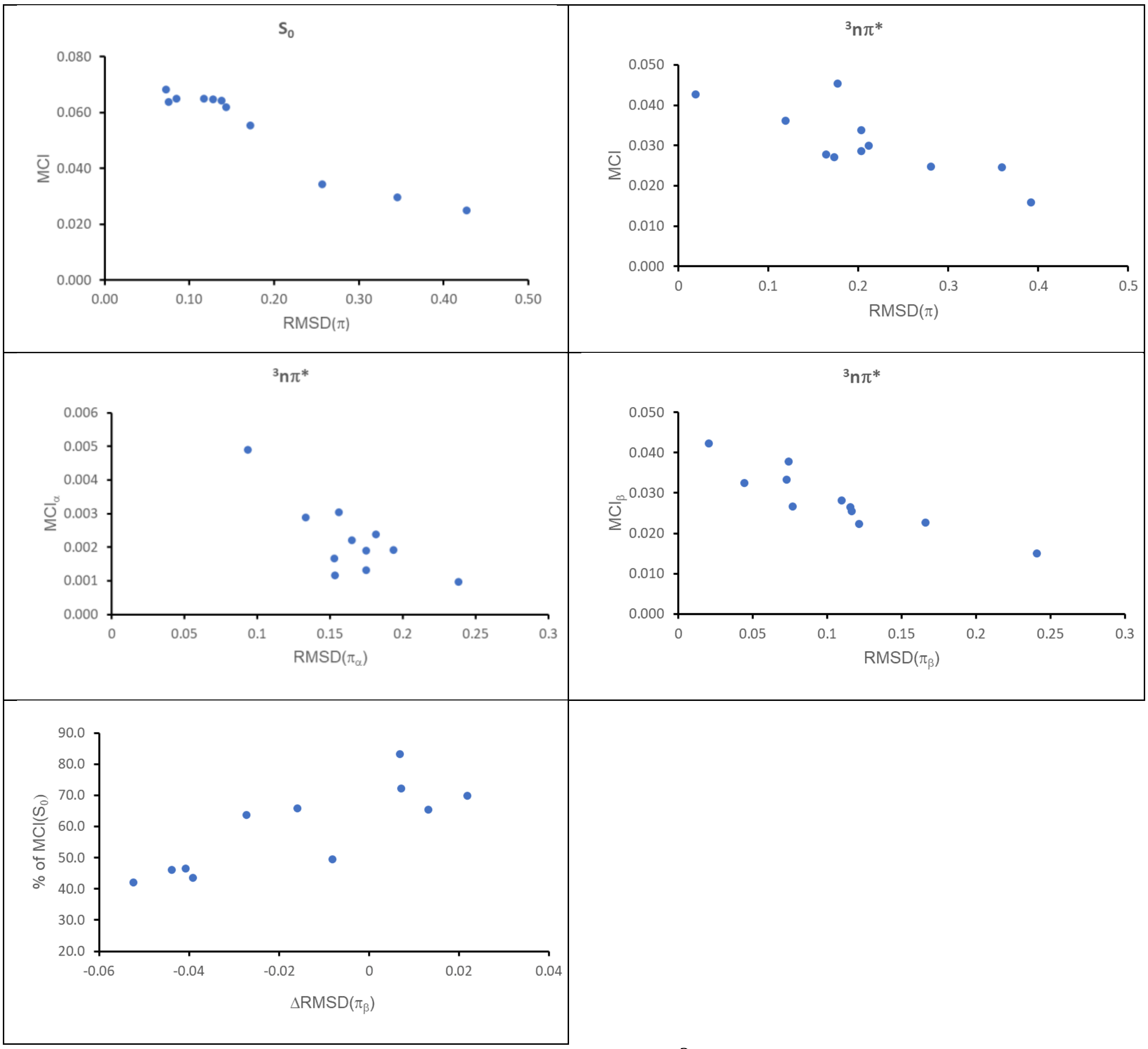

Figure S6. MCI results of the S_0 and the lowest vertical ^{3}n,π* state of the compounds of Group B versus the root mean square deviation of natural atomic p-orbital occupations (RMSD(π)) and % of MCI (S_0) versus the difference in RMSD(π_β) between the S_0 and the lowest vertical ^{3}n,π* states (ΔRMSD(π_β)).

3.4. Orbital Energies

Table S47. Absolute orbital energies of the compounds of group A, calculated at CAM-B3LYP/6-311+G(d,p) level of theory, as well as the difference between the unoccupied and the occupied orbitals. The π* orbitals are of type I (Figure 3B), unless otherwise state. Energies in eV.

	**n**	**π**	**π***	**E(π*-n)**	**E(π*- π)**
**1**	-8.90	-8.93	0.18	9.08	9.11
	HOMO	HOMO-1	LUMO		
**2**	-9.11	-8.30	-0.48	8.63	7.82
	HOMO-2	HOMO	LUMO		
**3**	-0.98	-2.86	5.63	6.61	8.49
	HOMO	HOMO-1	LUMO+5 (type II)		
			6.79	7.77	9.66
			LUMO+6 (type I)		
**4**	-2.10	-2.05	4.44	6.54	6.49
	HOMO-1	HOMO	LUMO+2		
**5**	-17.41	-15.33	-6.97	10.44	8.36
	HOMO-2	HOMO	LUMO		
**6**	-16.80	-15.02	-6.92	9.89	8.10
	HOMO-2	HOMO	LUMO		

Table S48. Caption: Orbital shapes and energies of compounds **1** and **9**, as well as of ***s***-triazine, computed at CAM-B3LYP/6-311+G(d,p) level of theory. Energies in a.u.

	**HOMO-2**	**HOMO-1**	**HOMO**	**LUMO**	**LUMO+1**
**1**	-0.3554	-0.3282	-0.3271	0.0067	0.01975
	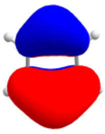	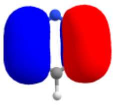	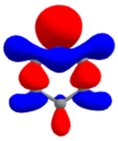	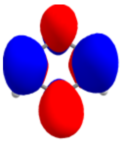	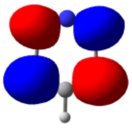
**9**	-0.3910	-0.3443	-0.3244	-0.0198	0.00745
	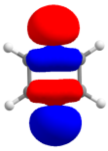	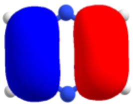	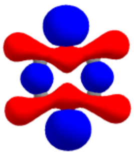	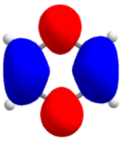	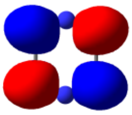
**s-triazine**	-0.4091	-0.3538	-0.3538	-0.0221	-0.0221
	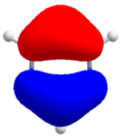	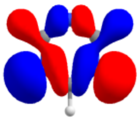	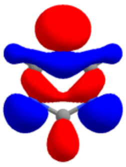	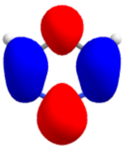	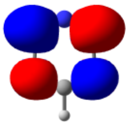

3.5. Relaxation

The only Group A compound with a relaxed T_1 state of pure n,π*character is **3**, and upon relaxation from its vertical ^{3}n,π* state, it shifts to a planar, anti-quinoidal structure. The degree of aromaticity is lowered slightly according to MCI, but remains relatively high. Curiously, this ^{3}n,π* state is the 1^3A_2 state (type II, Figure 3B), opposite to the lowest vertically excited ^{3}n,π* state, which is of B_1 symmetry (type I). The minimum of the latter state is only 0.15 eV higher in energy, and its relaxed structure is puckered but slightly more aromatic according to MCI and HOMA. Compounds **2**, **5**, and **6** keep planar structures in their relaxed ^{3}n,π* states, although, these states are 1.14 – 3.57 eV above the T_1 states, which are of π,π* character. Conversely, **1** and **4** pucker in their ^{3}n,π* states and become mixed n,π*/π,π* in character. Furthermore, for the three diazines, the C and N atoms of **9** (with a clear aromatic residual in the vertically excited state) remain in a nearly planar ring (the H atoms pucker out-of-plane by 5 – 32°) while **7** and **8**, with no aromatic residual, pucker.

Table S49. Absolute energies of the relaxed S_0 and T_1 states of compounds **1-9**, as well as the energy difference between these minima, calculated at CAM-B3LYP/6-311+G(d,p) level of theory.

	**Absolute Energy [Ha]**		**Energy Difference [eV]**
**Compound**	**S_0**	**Lowest opt ^{3}n,π***	**E(^{3}n,π*-S_0)**
**1**	-248.213108124	-248.078443987	3.66
**2**	-534.825465262	-534.725827597	2.71
**3 (type II)**	-231.519502370	-231.442091440	2.11
**3 (type I)**	-231.519502370	-231.436642816	2.25
**4**	-482.970830497	-482.880944664	2.45
**5**	-268.404038212	-268.280333784	3.37
**6**	-591.401412951	-591.284082665	3.19
**7**	-264.220904251	-264.124359174	2.63
**8**	-264.258906256	-264.125488412	3.63
**9**	-264.251656497	-264.124245853	3.47

Table S50. MCI results of the relaxed S_0 and T_1 states of compounds **1-9**, calculated at CAM-B3LYP/6-311+G(d,p) level of theory.

	**S_0**		**Lowest vertical ^{3}n,π***						
**Comp.**	**MCI**	**50% of MCI(S_0)**	**Character**	**MCI**	**% of MCI(S_0)**	**$MCI_α$**	**% of MCI(S_0)**	**$MCI_β$**	**% of MCI(S_0)**
**1**	0.0674	0.0337	n,π*- π,π* mix	0.0148	22%	0.0032	5%	0.0116	17%
**2**	0.0678	0.0339	π,π*	0.0009	1%	0.0016	2%	-0.0007	-1%
**3**	0.0619	0.031	n,π* (type II)	0.0361	58%	0.0017	3%	0.0345	56%
			n,π* (type I)	0.0394	64%	0.0055	9%	0.0338	55%
**4**	0.0419	0.021	n,π*- π,π* mix	0.0101	24%	0.001	2%	0.009	22%

**5**	0.0293	0.0146	π,π*	0.0049	17%	0.0044	15%	0.0005	2%
**6**	0.0562	0.0281	π,π*	-0.0014	-3%	0.0025	4%	-0.004	-7%
**7**	0.0682	0.0341	n,π*	0.0267	39%	0.0014	2%	0.0254	37%
**8**	0.0643	0.0321	n,π*	0.017	26%	0.0023	4%	0.0147	23%
**9**	0.0648	0.0324	n,π*	0.033	51%	0.003	5%	0.0299	46%

Table S51. π-electron ring current strengths (in nA T^{-1}) calculated as the average of all bonds in the given ring.

Group	Compound	S$_0$		Vertical ^{3}n,π*			Optimized ^{3}n,π*		
		total	total$_{\alpha/\beta}$	total	total$_\alpha$	total$_\beta$	total	total$_\alpha$	total$_\beta$
A	**1**	11.2	5.6	-19.9	-25.5	5.6	-4.7	-4.9	0.2
	**2**	11.3	5.7	-9.8	-14.4	4.6	-6.7	-3.2	-3.5
	**3 (type II)**	11.2	5.6	-83.5	-89.4	5.9	-30.7	-36.8	6.1
	**3 (type I)**	11.2	5.6	-83.5	-89.4	5.9	-30.6	-36.3	5.7
	**4**	10.0	5.0	-11.2	-16.0	4.8	-6.6	-6.0	-0.6
	**5**	9.3	4.6	-5.1	-9.0	3.8	-14.5	-10.2	-4.3
	**6**	10.5	5.3	-10.8	-9.0	-1.8	-7.0	-3.3	-3.7
B	**7**	10.7	5.3	-15.8	-21.9	6.1	-9.1	-13.8	4.8
	**8**	10.4	5.2	-36.2	-40.8	4.6	-5.2	-5.8	0.6
	**9**	10.8	5.4	-11.0	-19.3	8.3	-14.3	-21.5	7.2

Table S52. HOMA results for the different minima of compound **3**. Data are only shown for this species since it is the only compound in group A for which the T$_1$ state is of n,π* character. The calculations were performed at CAM-B3LYP/6-311+G(d,p) level of theory.

**Minimum**	**HOMA**
**S$_0$**	0.9403
**n,π* (type II)**	0.4757
**n,π* (type I)**	0.8444

3.6. Benchmark Calculations

The potential multireference character of excited states of monocyclic aromatic compounds is more important for the description of ππ* states, while single reference methods such as CCSD, KS-DFT (For T$_1$) or TD-DFT (For T$_n$ and S$_n$, n=1,2,3…) are expected to be useful for the study of the lowest excited states, specially of nπ* character, at the Franck-Condon region [49]. Still, we decided to perform CASSCF calculations to evaluate the multireference character of selected systems: **1**, **5**, **9-12**, **17** and **18**.

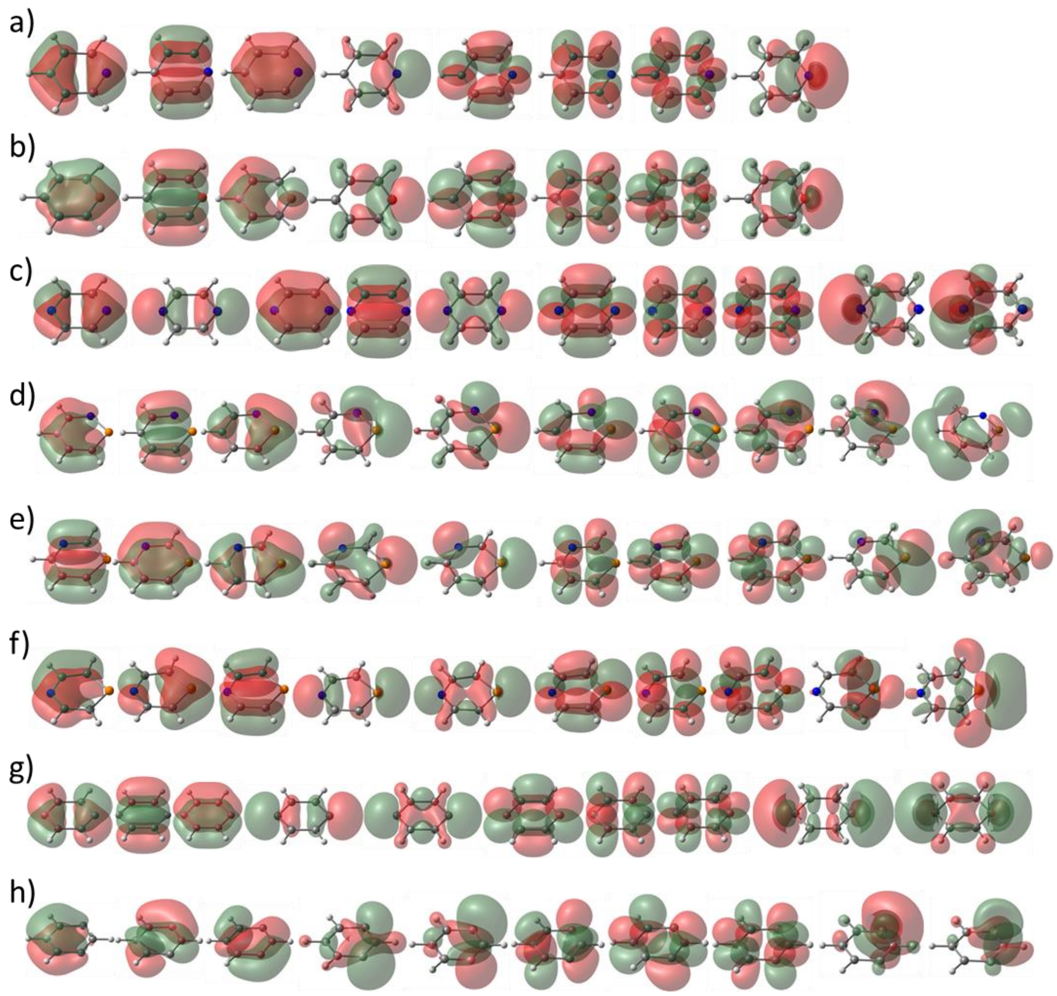

Figure S7. Natural orbitals selected for the active space of (a) **1**, (b) **5**, (c-f) **9-12**, and (g-h) **17-18**.

Table S53. Calculated Vertical Excitation Energies, in eV, for **1**, **5**, **9-12**, and **17-18**. The lowest singlet and triplet states of nπ* character are marked in bold.

system	state	character	configuration	weight	energy
**1**	S_0	-	22220000	0.86	0.00
	S_1	ππ*	2u22d000/u2220d00	0.46/0.31	5.12
	**S_2**	**nπ***	**222ud000**	**0.81**	**5.75**
	S_3	nπ*	222u0d00	0.81	6.56
	T_1	ππ*	2u220u00/u222u000	0.52/0.33	4.00
	T_2	ππ*	2u22u000/u2220u00	0.57/0.24	5.06
	**T_3**	**nπ***	**222uu000**	**0.84**	**5.13**
	T_4	nπ*	222u0u00	0.79	5.15
**5**	S_0	-	22220000	0.88	0.00
	S_1	ππ*	2u22d000/22u20d00	0.65/0.12	5.29
	S_2	ππ*	22u2d000/2u220d00	0.51/0.18	6.85
	**S_3**	**nπ***	**222ud000**	**0.81**	**7.46**
	S_6	nπ*	222u0d00/2u2d2000	0.63/0.16	9.71

	T_1	ππ*	2u22u000	0.92	4.23
	T_2	ππ*	22u2u000/2u220u00	0.70/0.21	5.25
	T_3	ππ*	2u220u00/22u2u000	0.68/0.19	5.77
	T_4	ππ*	22u20u00/2u2200u0	0.73/0.09	7.21
	**T_5**	**nπ***	**222uu000**	**0.85**	**7.25**
	T_7	nπ*	222u0u00/2u2u2000	0.71/0.11	9.55
**9**	S_0	-	2222200000	0.86	0.00
	**S_1**	**nπ***	**2222ud0000**	**0.79**	**4.77**
	S_2	ππ*	222u2d0000/u22220d000	0.52/0.23	5.12
	S_3	nπ*	2u222d0000/u222d20000	0.68/0.14	5.92
	S_4	nπ*	2222u0d000	0.78	5.96
	T_1	ππ*	222u20u000/22u22u0000	0.57/0.29	4.03
	**T_2**	**nπ***	**2222uu0000**	**0.80**	**4.12**
	T_3	ππ*	222u2u0000/22u220u000	0.72/0.12	4.83
	T_6	nπ*	2222u0u000	0.76	5.98
	T_7	nπ*	2u2220u000	0.61	7.19
**10**	S_0	-	2222200000	0.89	0.00
	**S_1**	**nπ***	**2222u0d000**	**0.81**	**4.72**
	S_2	nπ*	2222ud0000	0.82	5.44
	S_3	ππ*	22u22d0000/22u220d000	0.34/0.22	5.49
	T_1	ππ*	22u22u0000/22u220u000	0.55/0.31	3.85
	**T_2**	**nπ***	**2222uu0000**	**0.82**	**3.96**
	T_3	nπ*	222u2u0000/2222u0u000	0.51/0.31	4.81
**11**	S_0	-	2222200000	0.84	0.00
	S_1	ππ*	22u22d0000/u22220d000	0.38/0.28	4.50
	**S_2**	**nπ***	**2222u0d000**	**0.81**	**5.85**
	S_3	nπ*	2222ud0000	0.80	6.51
	T_1	ππ*	22u220u000	0.60	3.28
	T_2	ππ*	22u22u0000/u22220u000	0.48/0.29	4.56
	**T_3**	**nπ***	**2222uu0000/2222u0u000**	**0.27/0.49**	**4.67**
**12**	S_0	-	2222200000	0.86	0.00
	S_1	ππ*	2u222d0000/u22220d000	0.46/0.29	4.44
	**S_2**	**nπ***	**2222ud0000/22u22d0000**	**0.60/0.16**	**4.84**
	S_3	ππ*	22u22d0000/2222ud0000	0.53/0.17	5.89
	**T_1**	**nπ***	**2222uu0000/2u2220u000**	**0.56/0.13**	**2.67**
	T_2	ππ*	2u222u0000/u22220u000	0.48/0.19	3.69
	T_3	ππ*	2u2220u000	0.58	3.96
**17**	S_0	-	2222200000	0.88	0.00
	**S_1**	**nπ***	**2222ud0000**	**0.83**	**3.88**
	S_2	nπ*	222u2d0000	0.80	4.75
	S_3	nπ*	2222u0d000	0.81	4.81
	S_5	nπ*	222u20d000	0.75	5.49

	**T_1**	**nπ***	**2222uu0000/222u2u0000**	**0.51/0.31**	**4.05**
	T_2	nπ*	2222u0u000/222u20u000	0.62/0.21	4.17
	T_3	ππ*	2u2220u000/u2222u0000	0.53/0.34	4.19
	T_5	nπ*	222u2u0000/2222uu0000	0.48/0.28	4.89
	T_6	nπ*	222u20u000/2222u0u000	0.57/0.21	5.03
**18**	S_0	-	2222200000	0.89	0.00
	**S_1**	**nπ***	**2222ud0000**	**0.83**	**3.14**
	S_2	nπ*	222u2d0000	0.84	3.86
	S_3	nπ*	2222u0d000	0.79	4.50
	S_4	nπ*	222u20d000	0.79	5.43
	**T_1**	**nπ***	**2222u0u000/2222uu0000**	**0.40/0.23**	**2.99**
	T_2	nπ*	2222uu0000/2222u0u000	0.60/0.23	3.14
	T_3	nπ*	222u2u0000/2222u0u000	0.60/0.09	4.40
	T_4	nπ*	222u20u000/222u2u0000	0.52/0.15	4.58

From the CASSCF results in Table S53 we observe that in most cases the first singlet and triplet nπ* states are well described by only one configuration with a weight of ~0.8. Thus, multireference approach is not required to properly describe this excitation. However, this is not the case for the nπ* triplet states of **11**, **12**, **17** and **18** which appear to be described by more than one configuration at this level of theory.

3.7. 5-MRs

Throughout our study we noted that 5-MR heteroaromatics with one or two heteroatoms such as thiophene, furan and imidazole have their lowest nπ* states as higher-lying triplet and singlet excited states, never as T_1 and S_1. We asked what is the reason for the fact that the nπ* states of these compounds are higher-lying excited states? First, one can note that the calculated orbital energies for the highest occupied n and π-orbitals ($\varepsilon(n_\sigma)$ and $\varepsilon(\pi_3)$, respectively) are such that the n_σ orbital of the 5-MR heterocycles are generally lower in energy relative to that of π_3 orbital, compared to those of the 6-MRs with the same heteroatom (Figure S8A). Also the lowest unoccupied π-orbital (π_4*) is relatively higher in energy than the π_3 orbital when comparing the 5-MRs with their corresponding 6-MRs, although the difference is much smaller. This shows that the nπ* states are higher in energy in the smaller rings mainly due to the stabilization of the n_σ orbital.

A

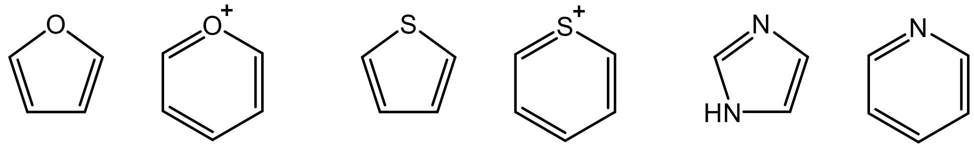

	Furan	Pyrylium	Thiophene	Thiopyrylium	Imidazole	Pyridine
Θ(C-E-C):	107.0	122.0	91.5	103.5	105.6	117.5
$\varepsilon(\pi_4^*) - \varepsilon_{HOMO}$:	9.19	8.36	8.81	8.10	9.50	9.08
$\varepsilon_{HOMO} - \varepsilon(n_\sigma)$:	4.08	2.09	2.96	1.78	1.21	0.00

B

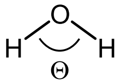

Θ(H-O-H):	100.0	105.0	110.0	115.0	120.0	125.0
$\varepsilon(\pi^*)$:	5.138	5.141	5.144	5.148	5.151	5.154
$\varepsilon(\pi)$:	-10.68	-10.66	-10.64	-10.62	-10.60	-10.57
$\varepsilon(n_\sigma)$:	-12.90	-12.65	-12.41	-12.17	-11.94	-11.72

Energies in eV and angles in degrees.

Figure S8. (A) Orbital energy differences between the orbital into which the electron is excited (π_4*), the HOMO and the orbital from which the excited electron leaves (n_σ). The π_4* orbital is the LUMO in all cases except furan and imidazole, for which it is the LUMO+1 and LUMO+2, respectively. For all shown compounds, n_σ is the HOMO-2 and π_3 is the HOMO, except for pyridine, for which the n_σ orbital is high enough in energy to be the HOMO. (B) Orbital energies of the π*, π and the n_σ orbitals of H_2O and how these vary with the H-O-H angle. The energies were calculated at UCAM-B3LYP/6-311+G(d,p) level.

As is well-established, for AH_2 molecules there is a close relationship between the n_σ (a_1) symmetric orbital and the H-A-H angle Θ so that the orbital energy goes to lower values as the angle becomes more acute (as described on pages 136-139 of Ref. 50). This is illustrated for H_2O with $100 \leq \Theta \leq 120$ in Figure S8B. Our findings for the 6- and corresponding 5-MRs match this expectation.

3.8. Order and Energy of Excited States vs. MCI and NICS(1)$_{zz}$

Here, the data that Figures 9A-C are based on are presented.

3.8.1. All heteroaromatics

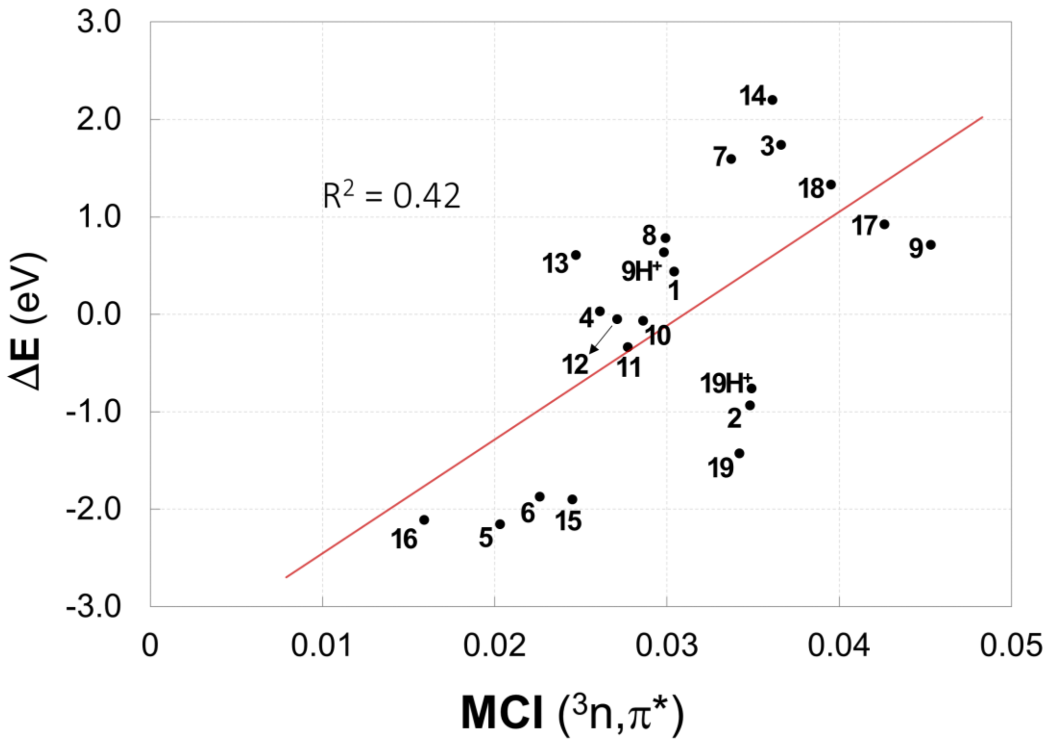

Figure S9. The Δ*E*($^3\pi,\pi^*$ - $^3n,\pi^*$) *vs.* MCI($^3n,\pi^*$) for **1** – **19H$^+$** as presented in Figure 9A, with the compound labels for each data point.

Table S54. Energies of the $^3\pi,\pi^*$ and $^3n,\pi^*$ states and Δ*E*($^3\pi,\pi^*$ - $^3n,\pi^*$) in eV, as well as MCI and NICS(1)$_{zz}$ aromaticity results for **1** – **19H$^+$**, calculated at CAM-B3LYP/6-311+G(d,p) level of theory.

Compound	*E*($^3n,\pi^*$)	*E*($^3\pi,\pi^*$)	Δ*E*($^3\pi,\pi^*$ - $^3n,\pi^*$)	MCI($^3n,\pi^*$)	NICS(1)$_{zz}$ ($^3n,\pi^*$)
**1**	4.30	4.74	0.44	0.0304	53.3
**2**	4.22	3.28	-0.94	0.0348	26.8
**3**	2.60	4.34	1.74	0.0366	213.4
**4**	2.88	2.90	0.03	0.0261	29.6
**5**	5.79	3.64	-2.15	0.0203	14.4
**6**	5.51	3.63	-1.87	0.0226	25.9

**7**	2.95	4.55	1.59	0.0337	42.8
**8**	4.13	4.91	0.78	0.0299	96.3
**9**	3.59	4.30	0.71	0.0453	30.3
**10**	3.34	3.27	-0.07	0.0286	41.6
**11**	3.60	3.26	-0.34	0.0277	96.2
**12**	3.56	3.51	-0.05	0.0271	30.0
**13**	2.44	3.05	0.61	0.0247	5.8
**14**	2.39	4.59	2.20	0.0361	82.6
**15**	3.83	1.93	-1.90	0.0245	-4.7
**16**	5.56	3.44	-2.11	0.0159	25.9
**17**	3.06	3.98	0.92	0.0426	172.4
**18**	2.75	4.08	1.33	0.0395	241.5
**19**	3.96	2.53	-1.43	0.0342	30.1
**9H$^+$**	2.98	3.61	0.63	0.0298	17.8
**19H$^+$**	3.38	2.62	-0.76	0.0349	25.7

3.8.2. Substituted pyrazines

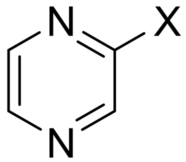

Figure S10. Substituted pyrazine.

Table S55. Energies of the $^3\pi,\pi^*$ and $^3n,\pi^*$ states, and $\Delta E(^3\pi,\pi^* - ^3n,\pi^*)$, in eV of substituted pyrazines, calculated at CAM-B3LYP/6-311+G(d,p) level of theory.

X	$E(^3n,\pi^*)$	$E(^3\pi,\pi^*)$	$\Delta E(^3\pi,\pi^* - ^3n,\pi^*)$
H	3.59	4.30	0.71
Me	3.60	4.10	0.50
SiH_3	3.35	4.13	0.78
$SiMe_3$	3.34	4.12	0.79
CF_3	3.58	4.24	0.65
Cl	3.76	4.07	0.31
F	3.84	4.03	0.19
OH	3.85	3.75	-0.11
NH_2	3.84	3.48	-0.36
CN	3.56	3.92	0.36
NO_2	3.59	4.02	0.43
C(=O)H	3.44	3.81	0.37
C(=O)NH_2	3.52	4.00	0.49

Table S56. MCI and NICS(1)$_{zz}$ results of the lowest vertical ^{3}n,π* states, as well as MCI results of the S_0 state, of substituted pyrazines, calculated at CAM-B3LYP/6-311+G(d,p) level of theory.

X	MCI(S_0)	MCI(^{3}n,π*)	NICS(1)$_{zz}$ (^{3}n,π*)
H	0.0648	0.0453	30.3
Me	0.0607	0.0430	25.7
SiH_3	0.0642	0.0431	30.2
$SiMe_3$	0.0634	0.0423	31.5
CF_3	0.0619	0.0425	31.8
Cl	0.0596	0.0393	29.8
F	0.0573	0.0348	22.5
OH	0.0515	0.0311	18.2
NH_2	0.0492	0.0323	16.6
CN	0.0592	0.0417	32.9
NO_2	0.0602	0.0416	33.3
C(=O)H	0.0595	0.0402	35.8
C(=O)NH_2	0.0602	0.0402	32.9

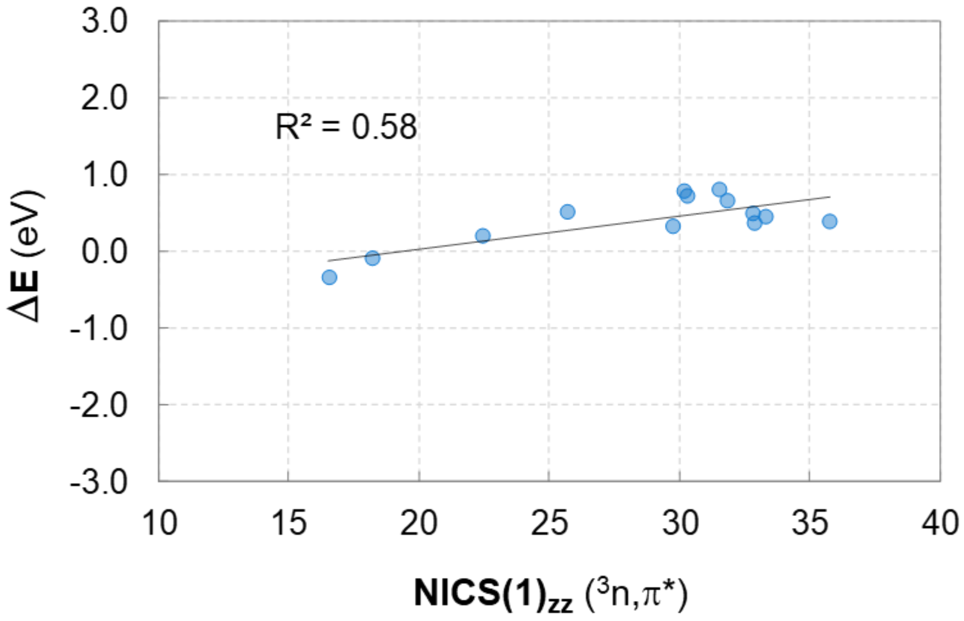

Figure S11. Δ*E*(^{3}π,π* - ^{3}n,π*) *vs.* NICS(1)$_{zz}$(^{3}n,π*) for **9** and monosubstituted pyrazines.

3.9. Distorted pyrazines

Here, the data that Figure 9D is based on are presented.

We evaluated the aromaticity in S_0 and T_1 states for an ensemble of non-equilibrium geometries of pyrazine. For a detailed description of the computational protocol, we refer to the Computational Details Section. Pyrazine was selected for being one of the systems with a residual of significant aromatic character in the lowest $^3n\pi^*$ state. The T_1 energies of these distorted geometries average around 3.40 eV, as shown in panel A of Figure S12, slightly lower than the vertical excitation energy of 3.59 eV at the equilibrium geometry. Despite this difference, the values are within an acceptable range for our analysis.

In order to determine the role of (anti)aromaticity in non-equilibrium geometries we analyzed the cases with notably high and low energy differences between the S_0 and T_1. In particular, values above 3.7 eV and below 3.1 eV, corresponding to the extremes of the scattered results from the Wigner sampling represented in panel E of Figure S12. In both cases, the S_0 MCI values remained closely aligned with those at equilibrium, deviating by no more than 0.007 units. However, T_1 showed greater deviations, averaging 0.015, and often resulting in a non-aromatic structure (MCI < 0.028). These results indicate that the aromaticity in the T_1 state is more susceptible to geometric distortions compared to S_0, making T_1 less stabilized by aromaticity when distorted.

Elaborating further on the influence of molecular conformation on aromaticity, we observe the following: The calculated MCI values for S_0 show a range close to the MCI value at the equilibrium geometry, which is 0.065 (panel B). In contrast, T_1 MCI values exhibit a broader range, varying from 0.002 to 0.048, and are not centered around the equilibrium MCI value of 0.045 (see Panel C and D). We attribute the wider distribution of T_1 MCI values to two primary factors. First, an inherent reduced aromaticity: The T_1, specifically the $^3n\pi^*$ state, presents 70% of the aromaticity of the ground state. Thus, distortions that diminish aromaticity have a more pronounced impact on this state. Second, a change in the excited state character: Depending on the distortion, the lowest excited triplet can change from n,π^* (like in the vertical case) to a state involving mixed n-π orbitals, which may be non-aromatic or even a π,π^* state, which will be antiaromatic. This results in an even more substantial decrease in T_1 MCI values compared to the equilibrium $^3n\pi^*$ state.

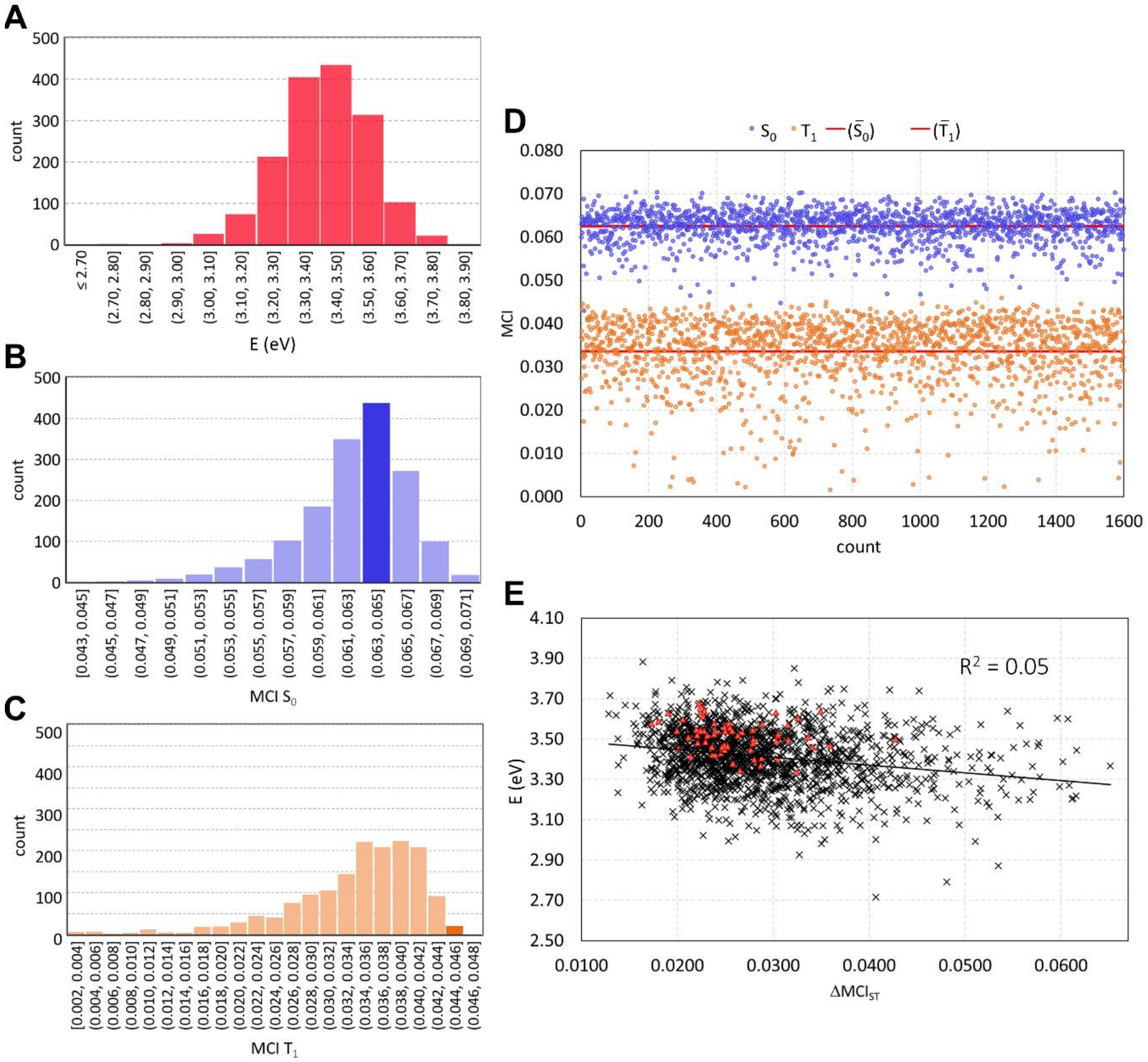

Figure S12. Analysis of MCI values for pyrazine based on an ensemble of 1600 geometries obtained through normal mode following algorithm. (A) Distribution of triplet excitation energy values (in eV). (B) Histogram of MCI values for the S_0 state, indicating a consistent range between 0.045–0.071, aligning with the MCI value of the equilibrium geometry of 0.065. (C) Histogram of MCI values for the T_1 (either n,π* or π,π*) state, showing a broader range 0.002–0.048, with the vertical triplet n,π* reference value being 0.045. (D) Comparison of S_0 (blue) and T_1 (orange) MCI values over the ensemble. (E) Correlation plot indicating the variance in T_1 vertical energies (eV) as a function of the difference in singlet and triplet MCI (ΔMCI_{ST}). The T_1 state arises from transitions between various orbitals (n,π*, π,π*, and mixed n-π) depending on the geometric distortion. Red points represent geometries where the S_0 state has an energy difference of less than 10.0 kcal/mol from its equilibrium value and correspond to the data used in Figure 9D.

3.10. Polyazaacenes

We also explored if the substituent effects on the order between the ^{3}n,π* and ^{3}π,π* states of pyrazines can be expanded to two bicyclic species containing pyrazine rings; quinoxaline (benzopyrazine) and 1,4,5,8-tetraazanaphthalene (Figure S13). For quinoxaline, the ^{3}n,π* and ^{3}π,π* states are isoenergetic, while for 1,4,5,8-tetraazanaphthalene the ^{3}n,π* state is well below the ^{3}π,π* state. Building on the finding for the monosubstituted pyrazines, we can indeed tune the E(^{3}n,π*) of quinoxalines relative to E(^{3}π,π*) by the substituents at the pyrazine ring (Figure S13). Thus, with silyl substituents at the pyrazine ring we achieve a quinoxaline with a ^{3}n,π* state as its T_1 state is 0.36 eV below the lowest ^{3}π,π* state. This points to a rationale for design of polyheteroaromatics with targeted states (n,π* or π,π*) as their lowest excited states, and it is noteworthy that substituted pyrazines recently were considered as replacements of analogous benzenes in photoluminescent materials [51].

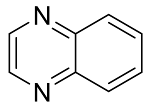

E(^{3}n,π*) = 3.34
E(^{3}π,π*) = 3.36
MCI(^{3}n,π*;6-MR) = 0.0287
MCI(^{3}n,π*;10-MR) = 0.0039

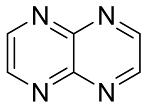

E(^{3}n,π*) = 2.96
E(^{3}π,π*) = 3.62
MCI(^{3}n,π*;6-MR) = 0.0280
MCI(^{3}n,π*;10-MR) = 0.0021

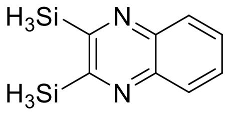

E(^{3}n,π*) = 2.95
E(^{3}π,π*) = 3.31
MCI(^{3}n,π*;6-MR) = 0.0257
MCI(^{3}n,π*;10-MR) = 0.0039

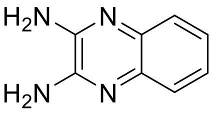

E(^{3}n,π*) = 4.20
E(^{3}π,π*) = 3.32
MCI(^{3}n,π*;6-MR) = 0.0194
MCI(^{3}n,π*;10-MR) = 0.0018

Figure S13: Polyazaacenes and the vertical excitation energies to ^{3}n,π* and ^{3}π,π* states in eV, together with MCI values of the pyrazine 6-MRs and the perimeter 10-MRs for the ^{3}n,π* state.

Table S57. MCI results of the S_0 and the lowest vertical ^{3}n,π* state of the polyazaacenes, calculated at CAM-B3LYP/6-311+G(d,p) level of theory, in part presented in Figure S13.

Molecule	Ring	S_0	Lowest ^{3}n,π*			
		MCI	MCI	% of MCI(S_0)	MCI_α	MCI_β
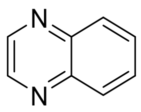	pyrazine 6-MR	0.0345	0.0287	0.8312	0.0027	0.0260
	benzene 6-MR	0.0359	0.0447	1.2448	0.0265	0.0182
	perimeter 10-MR	0.0082	0.0039	0.4774	-0.0003	0.0041

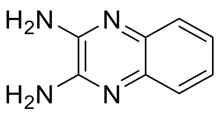	pyrazine 6-MR	0.0178	0.0194	1.0879	0.0021	0.0173
	benzene 6-MR	0.0421	0.0476	1.1299	0.0259	0.0217
	perimeter 10-MR	0.0042	0.0018	0.4266	-0.0001	0.0019
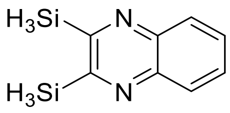	pyrazine 6-MR	0.0339	0.0257	0.7596	0.0027	0.0230
	benzene 6-MR	0.0354	0.0441	1.2452	0.0264	0.0177
	perimeter 10-MR	0.0080	0.0039	0.4821	-0.0003	0.0041
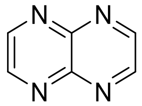	pyrazine 6-MR	0.0331	0.0280	0.8454	0.0091	0.0190
	perimeter 10-MR	0.0070	0.0021	0.2970	-0.0007	0.0028

3.11. Osmapyridinium and Osmapentalene complexes

Here we discuss further the osmapyridiniums **20** and **21**, which have been found through computations to be aromatic in both S_0 and T_1, a feature which has been labelled as *adaptive aromaticity* [52]. However, as presented in the main paper, the findings for T_1 states, which are of $^3\pi,\sigma^*$ and $^3\sigma,\pi^*$ character, can be explained within the general framework put forth by us for the $^3n,\pi^*$ states of heteroaromatics, as the $^3\pi,\sigma^*$ and $^3\sigma,\pi^*$ states also have differences in the number of π_α- and π_β-electrons. Thus, the aromaticity observed in the T_1 states of certain metallaaromatics arises when the residual between the two spin-components is not nil. As mentioned in the main text, we computed the spin-separate MCI and MICD of the two osmapyridiniums **20** and **21** [53], analysing both their vertical and relaxed $^3\sigma,\pi^*$ states. Noteworthy, the computed degree of aromaticity for the triplet σ,π^* or π,σ^* states of metallaaromatics labelled as adaptive aromatic is in most cases lower than for the closed-shell singlet state, which resembles what is observed for all heteroaromatics explored in their $^3n,\pi^*$ states when compared to S_0.

Our results for **20** and **21** compare qualitatively with previously reported trends, although our MCI values are lower. Now, based on the criteria set up in the main paper, only **21** should be considered to have an aromatic residual (Figure S14). The residual of osmapyridinium **20** in σ,π^* instead tends towards antiaromaticity, or alternatively, a nonaromatic character if based on the previously reported MCI results. In both cases, the residual is a result of a very low (negative) MCI_α-component and a higher MCI_β-contribution, with the latter having a greater impact on the residual, similar to what was found for the heteroaromatics.

The results from the MICD calculations generally support these findings. Both **20** and **21** sustain relatively strong diatropic current densities in the singlet state (Figures S14B and S15A), yet in the $^3\sigma,\pi^*$ state of **20** the global circulation practically completely vanishes, and in the corresponding state of **21** there are rather weak diatropic currents (Figures S14C and S15B). According to the calculated MICDs, **21** can be considered as aromatic in the singlet state, but only weakly aromatic in the $^3\sigma,\pi^*$ state. The aromatic character of **21** in the $^3\sigma,\pi^*$ state comes from both the diatropic current density contributions of π_β-electrons and from relatively weak

paratropic currents of π_α-electrons. The contribution of π_β-electrons in the $^3\sigma,\pi^*$ is less significant than the corresponding one in the singlet state. Therefore, the aromaticity of the singlet **21** is preserved in the triplet state mainly due to the fact that the α-HSOMO induce very weak paratropic current density contribution, which comes from a relatively large α-HOMO-LUMO gap (Table S62).

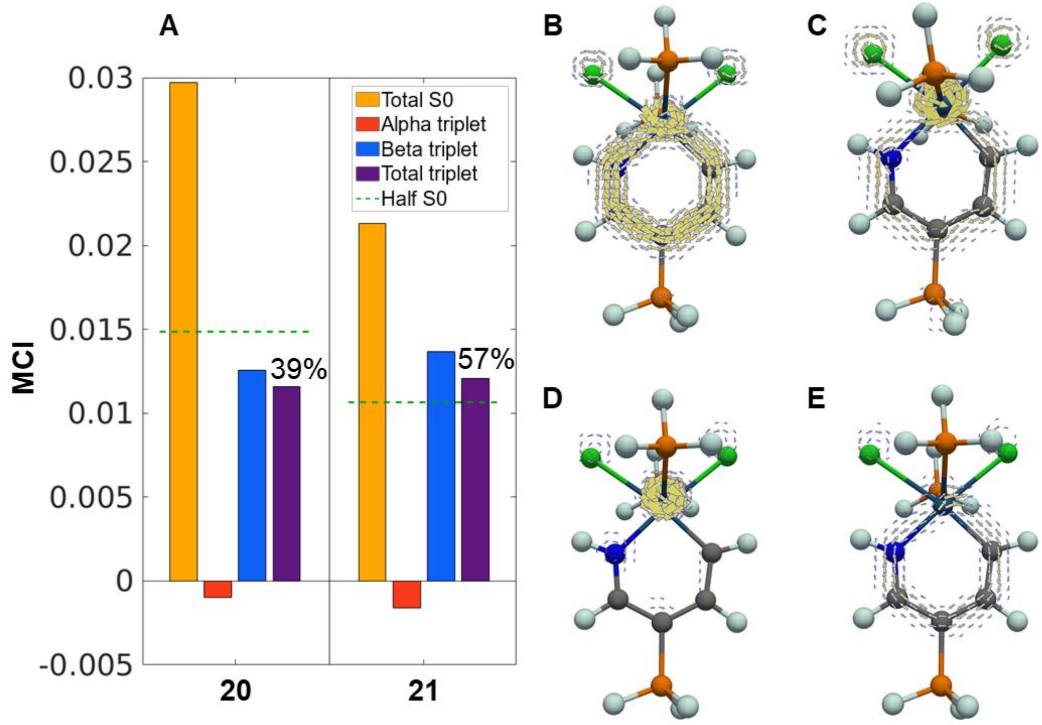

Figure S14: Results on the osmapyridiniums: (A) the vertically spin-separated MCI values; (B) – (E) π-electron MICD plots calculated 1 bohr above the molecular plane of **21**: S_0 state (B) and vertical $^3\sigma,\pi^*$ state (C) with the corresponding π_α- and π_β-electron contributions, (D) and (E). Clockwise circulation corresponds to diatropic (aromatic) currents.

Upon geometry relaxation, both compounds in their $^3\sigma,\pi^*$ states gain aromaticity according to MCI, and the relaxation energy reflects this change. When vertically excited from the optimal singlet state geometry, the $^3\sigma,\pi^*$ states of **20** and **21** are, respectively, 0.64 and 1.06 eV above the S_0 state, but after relaxation this changes to 0.12 and -0.20 eV (Tables S60 and S61), whereby the latter species has a triplet ground state (T_0), in line with previous findings [53]. Interestingly, the aromaticity gain comes about because of two different effects (Tables S58 and S59). For **20**, the increase in aromaticity mainly stems from an increased MCI_α-component, which means that the relaxation alleviates the antiaromatic π_α-contribution. In **21**, on the other hand, the MCI_α-component remains rather unchanged upon geometry relaxation while the aromaticity according to the MCI_β-component increases significantly.

However, unlike the MCI values, the calculated MICDs are practically insensitive to the effects of the geometry relaxation of the $^3\sigma,\pi^*$ state, in line with previous studies showing that MICD is mainly influenced by symmetry and nodal characteristics of the frontier orbitals, and far less by small geometry modifications [54].

Interestingly, according to MICD the vertically excited $^3\sigma,\pi^*$ states of both osmapyridiniums are even slightly more aromatic than the relaxed ones. A reason for the diatropic ring currents in **20** and **21** comes from a smaller paratropic π_α-contribution in their $^3\sigma,\pi^*$ states, in contrast to the $^3n,\pi^*$ states of the mono- and diheteroaromatic compounds. As the orbital energy gaps between α-HSOMO and α-LUMO are slightly larger (~0.25 a.u.) in the osmapyridiniums compared to the mono- and diheteroaromatics (0.14 – 0.23 a.u.), there would be larger paratropic contributions in the latter species. However, there should also be additional contributing factors that reduce the paratropicity in **20** and **21**. Thus, the absolute contributions of the spin components vary between electronic and magnetic descriptors.

Hence, it becomes clear from the osmapyridiniums that the approach of separating the α- and β-spin components of electronic states with different parity in the number of π_α- and π_β-electrons is a useful approach for analysis of the (anti)aromatic character of such states (see also the data in the tables below). It provides an overarching theoretical framework to rationalize computational observations of such states.

The data on which this discussion is based are presented in the tables below.

Table S58. MCI results of the S_0 and the lowest vertical $^3\sigma,\pi^*$ state of the osmapyridiniums, calculated at CAM-B3LYP/6-311+G(d,p) level of theory for all atom types except Os, for which the LANL2DZ basis set was used.

	**S_0**		**Lowest vertical $^3\sigma,\pi^*$**					
**Compound**	**MCI**	**50% of MCI(S_0)**	**MCI**	**% of MCI(S_0)**	**MCI_α**	**% of MCI(S_0)**	**MCI_β**	**% of MCI(S_0)**
**20**	0.0297	0.0149	0.0116	39%	-0.0010	-3%	0.01257	42%
**21**	0.0213	0.0107	0.0121	57%	-0.0016	-7%	0.0137	64%

Table S59. MCI results of the S_0 and the lowest optimised $^3\sigma,\pi^*$ state of the osmapyridiniums, calculated at CAM-B3LYP/6-311+G(d,p) level of theory for all atom types except Os, for which the LANL2DZ basis set was used. Coordinates for the optimised $^3\sigma,\pi^*$ states were obtained from Ref. 53.

	**S_0**		**Lowest optimised $^3\sigma,\pi^*$**					
**Compound**	**MCI**	**50% of MCI(S_0)**	**MCI**	**% of MCI(S_0)**	**MCI_α**	**% of MCI(S_0)**	**MCI_β**	**% of MCI(S_0)**
**20**	0.0297	0.0149	0.0122	41%	-0.0004	-1%	0.01258	42%
**21**	0.0213	0.0107	0.0149	70%	-0.0015	-7%	0.0164	77%

Table S60. Absolute energies of the S_0 and the lowest vertical $^3\sigma,\pi^*$ state, as well as the vertical excitation energy of the latter, of the osmapyridiniums. The energies were calculated at CAM-B3LYP/6-311+G(d,p) level of theory for all atom types except Os, for which the LANL2DZ basis set was used.

	**Absolute Energy [Ha]**		**Vertical Excitation Energy [eV]**
**Compound**	**S_0**	**Lowest $^3\sigma,\pi^*$**	**Lowest $^3\sigma,\pi^*$**
**20**	-1907.98429986	-1907.96088752	0.64
**21**	-2250.27152466	-2250.23253819	1.06

Table S61. Absolute energies of the S_0 and the lowest optimised $^3\sigma,\pi^*$ state, as well as the vertical excitation energy of the latter, of the osmapyridiniums. The energies were calculated at CAM-B3LYP/6-311+G(d,p) level of theory for all atom types except Os, for which the LANL2DZ basis set was used. Coordinates for the optimised $^3\sigma,\pi^*$ states were obtained from Ref. 53.

	**Absolute Energy [Ha]**		**Optimised Energy Relative to S_0 [eV]**
**Compound**	**S_0**	**Lowest $^3\sigma,\pi^*$**	**Lowest $^3\sigma,\pi^*$**
**20**	-1907.98429986	-1907.97989858	0.12
**21**	-2250.27152466	-2250.27891216	-0.20

Table S62. π-electron ring current strengths (in nA T^{-1}) of the osmapyridiniums, calculated as the average of all bonds in the given ring. For the vertical $^3n\pi^*$ state the total π_α–electron current strength was decomposed into the π_α-HSOMO contribution and the contribution of all other π_α orbitals (total - HSOMO). ΔE_{H-L} (in Hartrees) is the HOMO-LUMO energy gap for π_α orbitals.

Group	Compound	S_0		$^3n,\pi^*$					
		π	$\pi_{\alpha/\beta}$	π	π_α				π_β
					total	HSOMO	total - HSOMO	ΔE_{H-L}	
**C**	**20**	7.1	3.5	1.7	-0.8	-2.9	2.2	0.25	2.4
	**21**	8.9	4.5	3.2	-1.0	-2.5	1.5	0.26	4.2

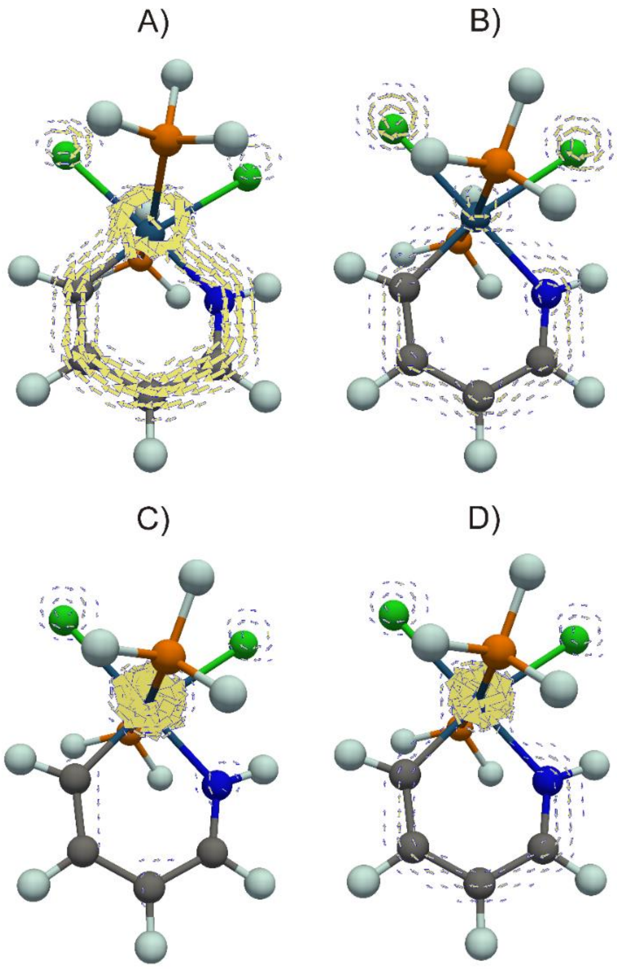

Figure S15. Maps of magnetically induced π-electron current densities calculated 1 bohr above the molecular plane of **20**: (A) S_0 state, (B) $^3\sigma,\pi^*$ state, (C) and (D) π_α- and π_β-electron contributions for the $^3\sigma,\pi^*$ state. Clockwise circulation corresponds to diatropic (aromatic) currents.

Additionally, the aromatic charter of two osmapentalenes (Figure S16) in their S_0 and T_1 states was explored. It has been demonstrated that **S1** exhibits so-called adaptive aromaticity, since through computations it is found to be aromatic in the both the S_0 and T_1 states [52]. On the other hand, **S2** was found to be aromatic in the S_0 and nonaromatic in the T_1. In what follows, we will show how the observed aromatic characteristics of the two osmapentalenes can be rationalized by using the spin separated MICDs.

First, it should be noted that the T_1 states of compounds **S1** and **S2** arise through different types of excitations. More specifically, the former is a state of π,σ^* character, whereas the latter is a σ,π^* state (and thus analogous to the heterocycles studied in in the main text). In the case of **S1**, the number of π_β electrons is reduced by one whereas the number of π_α electrons remains the same as in the S_0 state. Oppositely, for **S2** the number of π_β electrons remains unchanged and the number of π_α electrons increases by one. Both situations give rise to states with an odd number of π-electrons, even though the characters of the excited states are different.

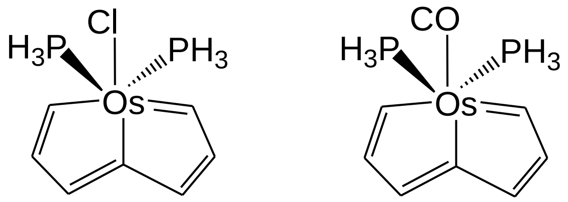

S1 **S2**

Figure S16. The structures of compounds **S1** and **S2**, previously investigated in Ref. 52.

π-electron current density maps were calculated for the S_0 and T_1 states of **S1** and **S2** (Figures S17 and S18). As can be seen from the current density plots, both molecules sustain diatropic (aromatic) currents in the S_0 state. In the T_1 state, **S1** exhibits diatropic global currents which are somewhat less intense than in the S_0 state. On the other hand, **S2** in the T_1 state practically has no global current densities, indicating a nonaromatic character of this molecule in this state. Our findings are in agreement with previously published results [52].

The observed T_1 state (non-)aromatic character of **S1** and **S2** can be understood by analysing the spin-separated MICD components. The diatropic current density in the T_1 state of **S1** predominantly comes from π_α electron contributions, while π_β electrons induce very weak currents (Figures S17C and S17D). Yet, in the T_1 state of **S2**, the π_α and π_β electrons induce currents of opposed tropicity, and of very similar intensity, which in total give negligibly small global current density (Figure S18). Thus, the *residuals* in these two cases are different, since adding the π_α- and π_β-contributions together lead to different aromatic character in the excited states of the two compounds. The residual can be either aromatic (as in the case of compound **S1**), non-aromatic (like compound **S2**) or antiaromatic. Insight into why either situation arises may only be gained by separating the spins, and it is important to realize that these different outcomes are all resulting from the fact that the states have an odd number of π-electrons.

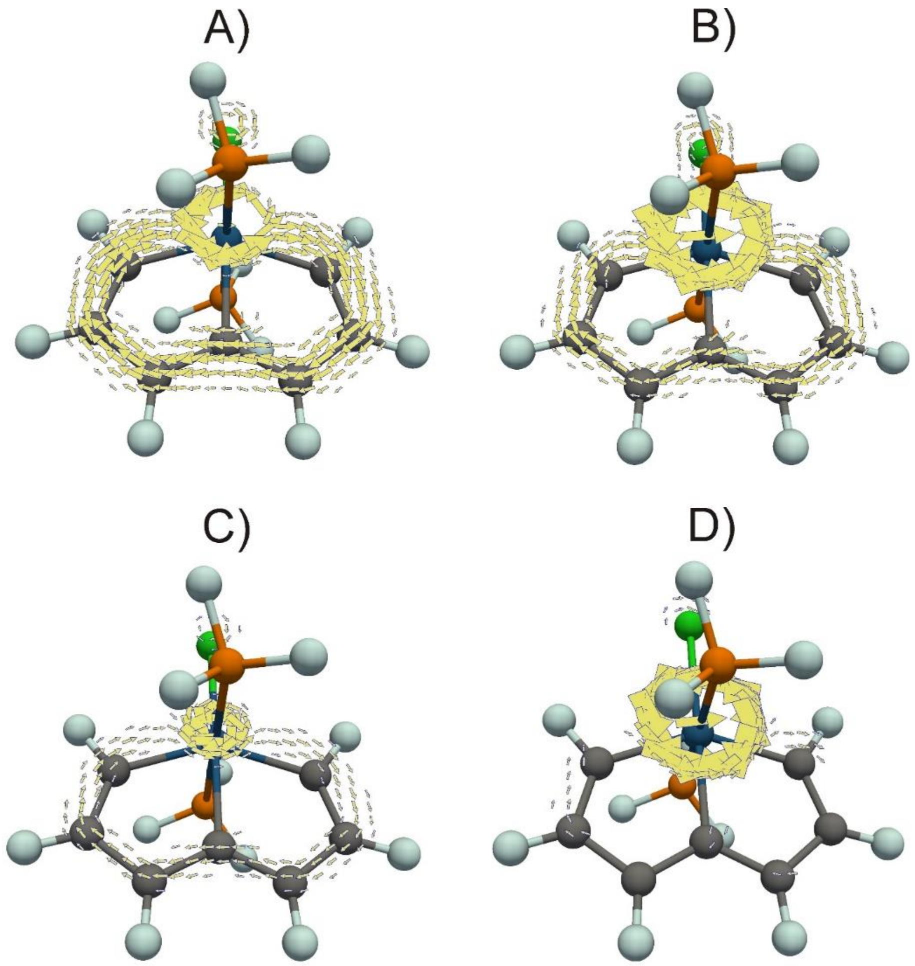

Figure S17. Maps of magnetically induced π-electron current densities calculated 1 bohr above the molecular plane of **S1**: (A) S_0 state, (B) $^3\pi,\sigma^*$ state, (C) and (D) π_α- and π_β-electron contributions for the $^3\pi,\sigma^*$ state. Clockwise circulation corresponds to diatropic (aromatic) currents.

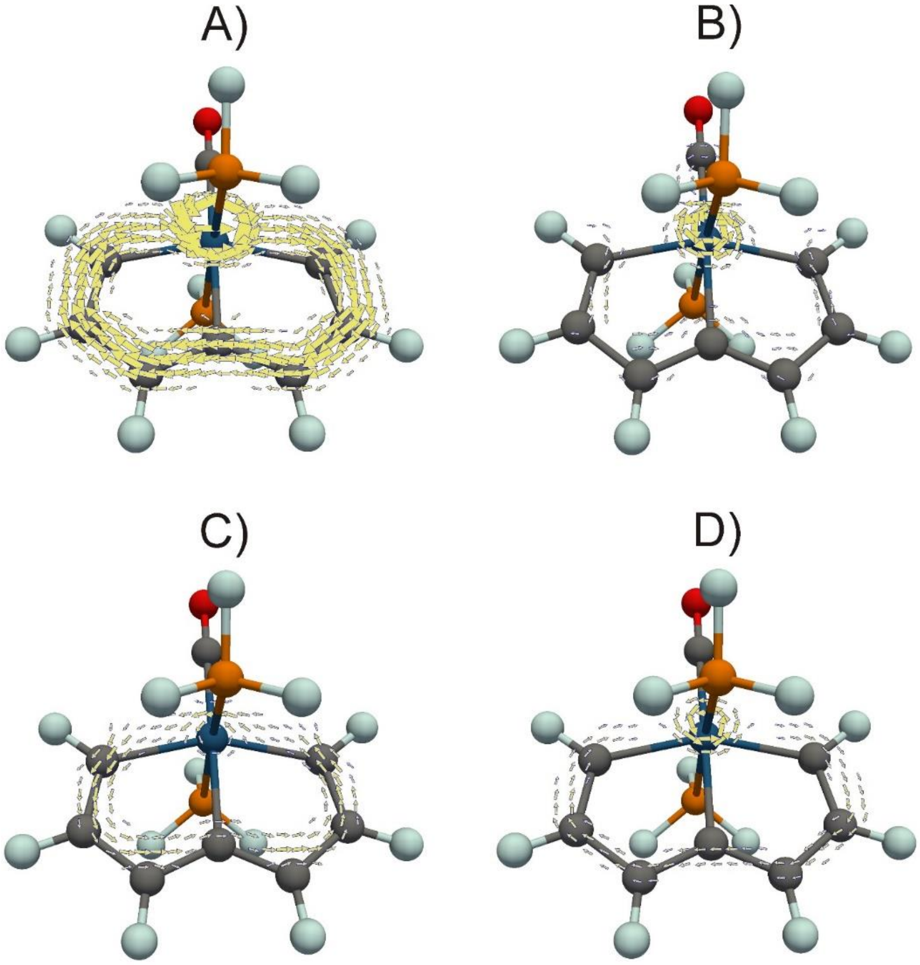

Figure S18. Maps of magnetically induced π-electron current densities calculated 1 bohr above the molecular plane of **S2**: (A) S_0 state, (B) $^3\sigma,\pi^*$ state, (C) and (D) π_α- and π_β-electron contributions for the $^3\sigma,\pi^*$ state. Clockwise circulation corresponds to diatropic (aromatic) currents.

4. Full-Scale MICD Plots

Clockwise circulation corresponds to diatropic (aromatic) currents, and *vice versa*.

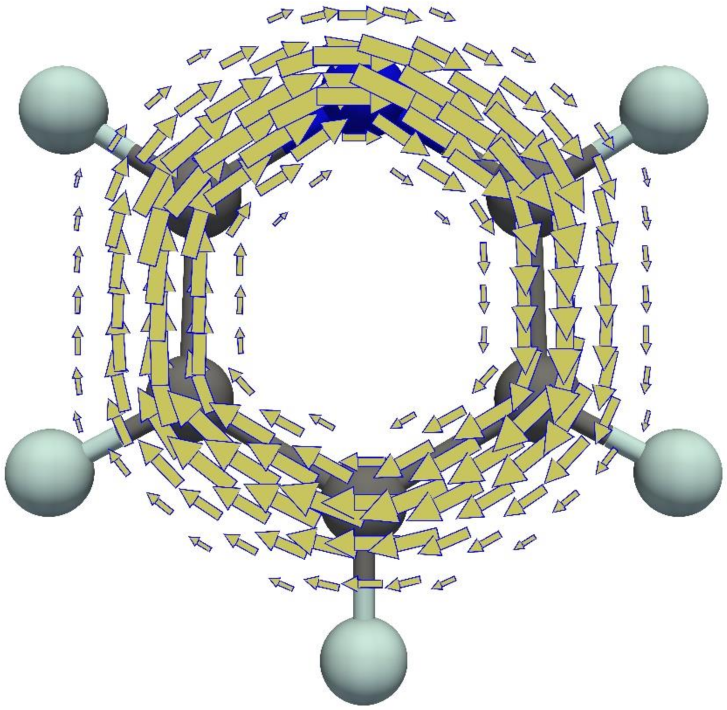

Figure S19. Map of the magnetically induced π-electron current density of the S_0 state of **1**, calculated 1 bohr above the molecular plane (Figure 6A).

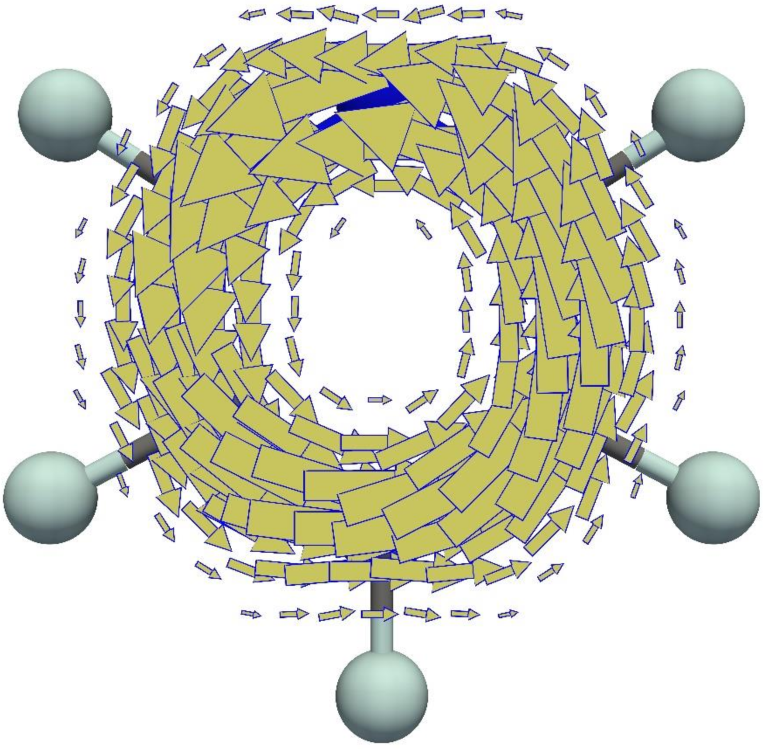

Figure S20. Map of the magnetically induced π-electron current density of the lowest vertical n,π* state of **1**, calculated 1 bohr above the molecular plane (Figure 6B).

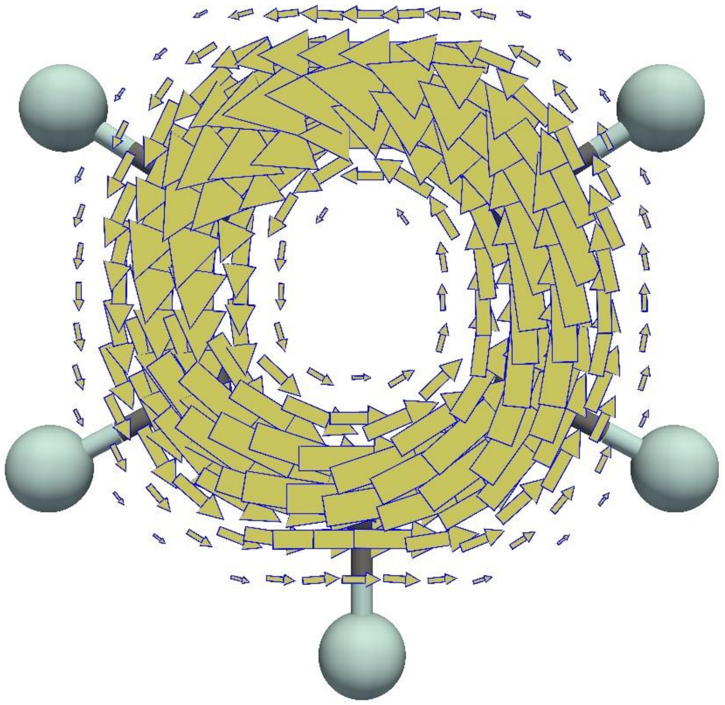

Figure S21. Map of the π_α-electron contribution to the magnetically induced π-electron current density of the lowest vertical n,π* state of **1**, calculated 1 bohr above the molecular plane (Figure 6C).

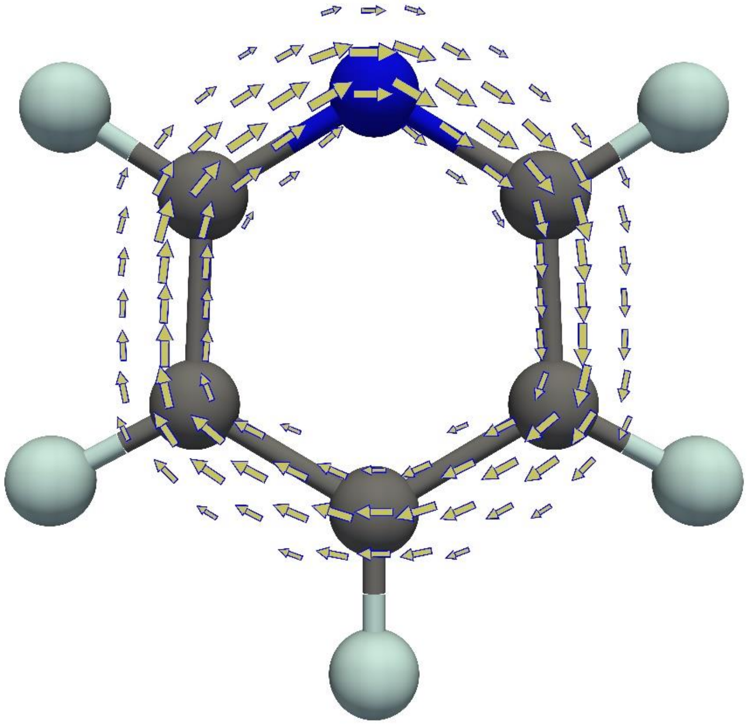

Figure S22. Map of the π_β-electron contribution to the magnetically induced π-electron current density of the lowest vertical n,π* state of **1**, calculated 1 bohr above the molecular plane (Figure 6D).

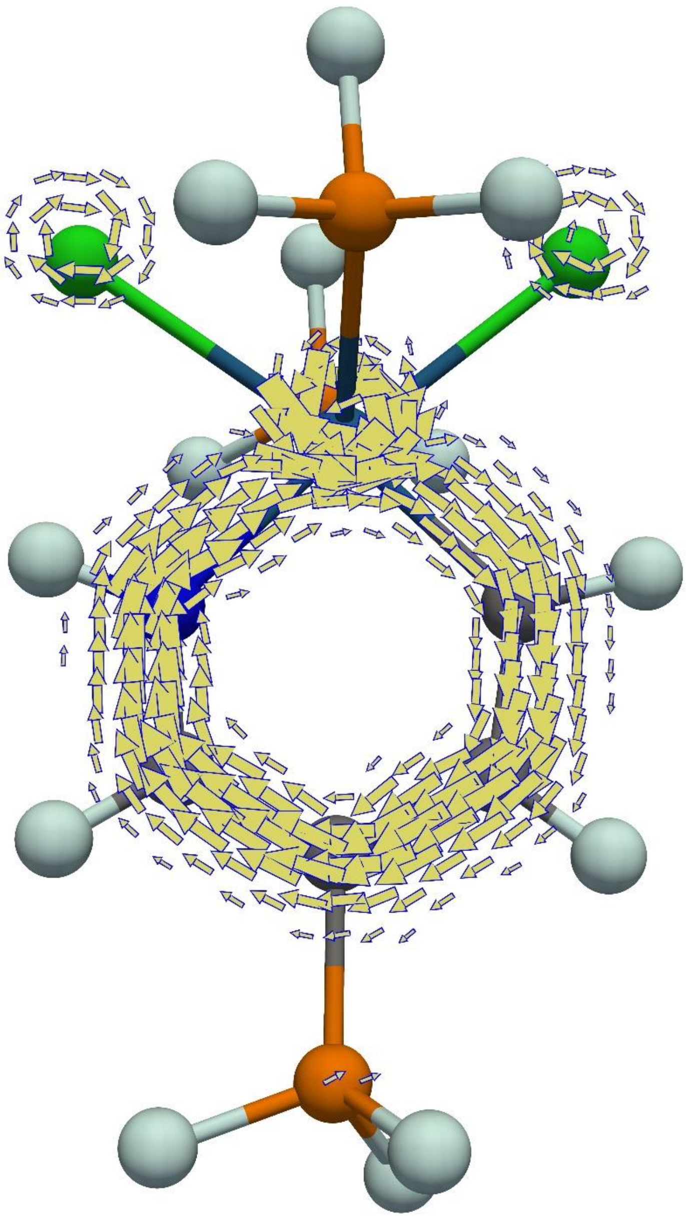

Figure S23. Map of the magnetically induced π-electron current density of the S_0 state of **21**, calculated 1 bohr above the molecular plane (Figure 10B).

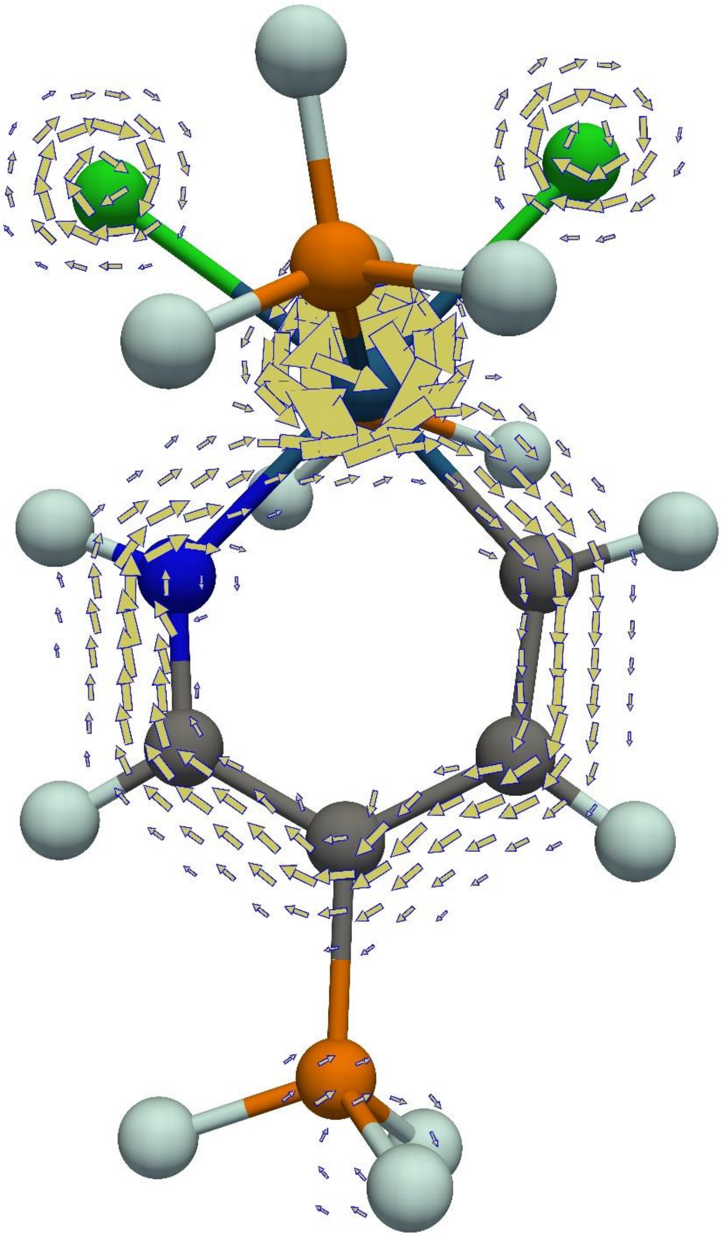

Figure S24. Map of the magnetically induced π-electron current density of the lowest vertical n,π* state of 21, calculated 1 bohr above the molecular plane (Figure 10C).

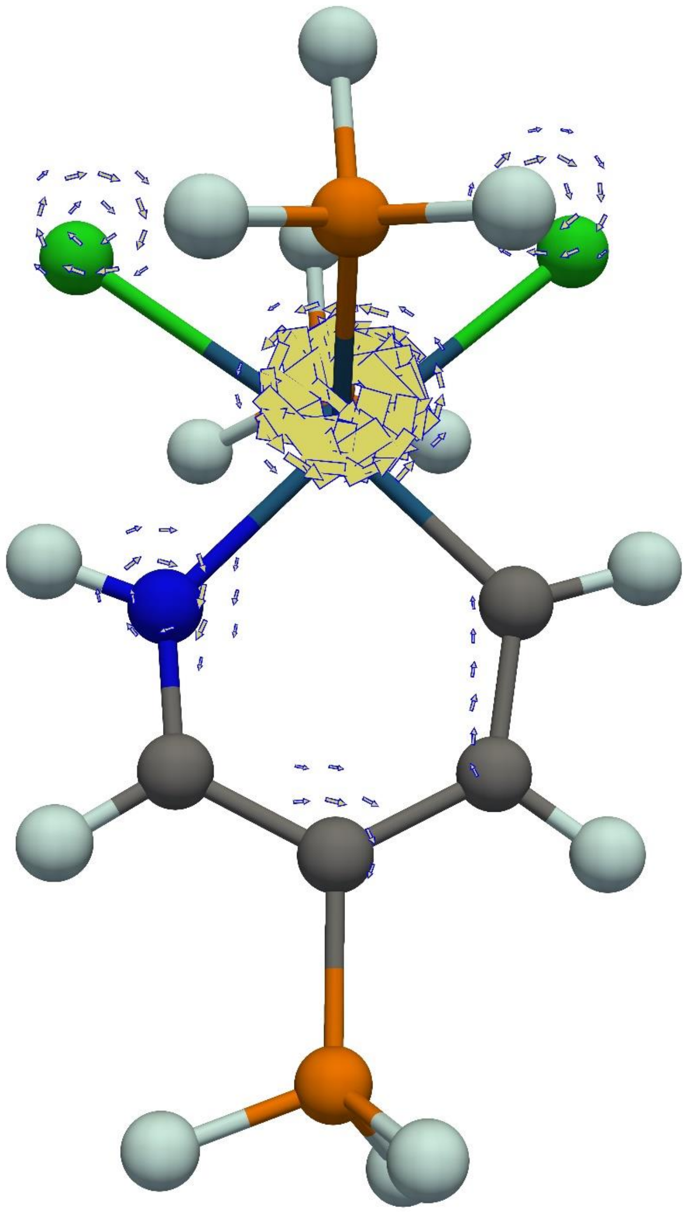

Figure S25. Map of the π_α-electron contribution to the magnetically induced π-electron current density of the lowest vertical n,π* state of **21**, calculated 1 bohr above the molecular plane (Figure 10D).

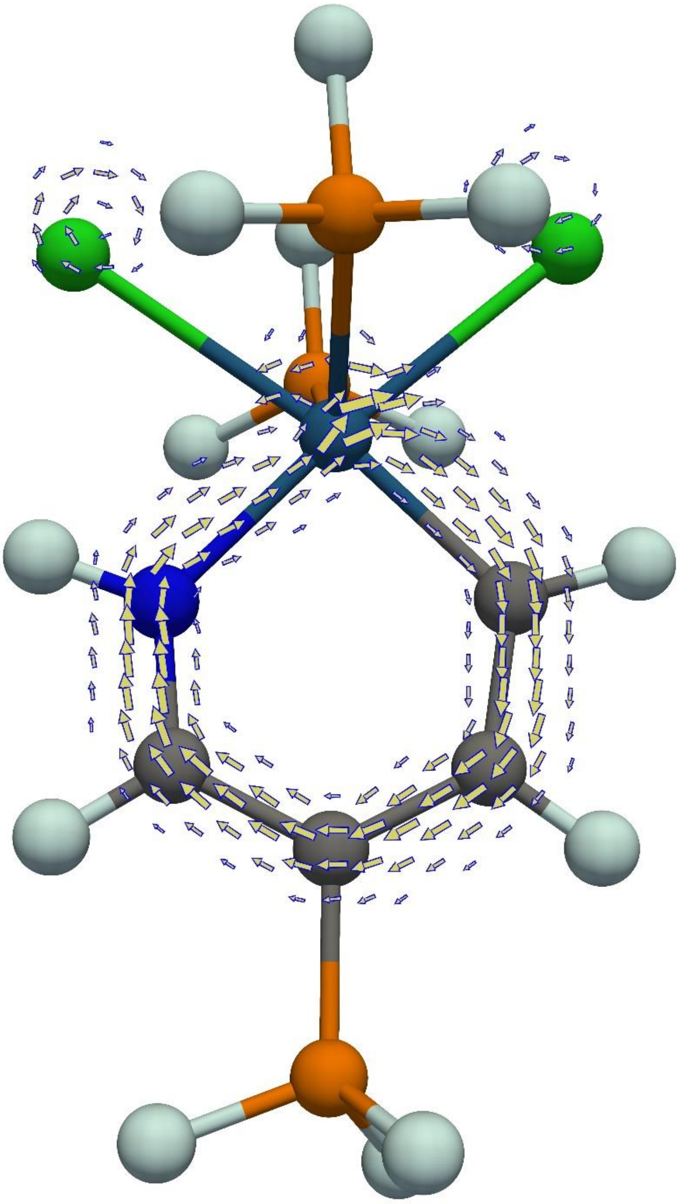

Figure S26. Map of the π_β-electron contribution to the magnetically induced π-electron current density of the lowest vertical n,π* state of 21, calculated 1 bohr above the molecular plane (Figure 10E).

5. Input Files and Procedures for Aromaticity Calculations

Table S63. Example describing the procedure of MCI calculations for **1** in the S_0 state.

A) Gaussian input file for producing the .wfx file needed as input for MCI calculations.

```
#p cam-b3lyp/6-311+g(d,p)  6d 10f out=wfx scf=qc

To get wfx file

0 1
 C          1.19177008   -0.66851505    0.00000000
 C          1.13720008    0.71850605    0.00000000
 C         -1.13720008    0.71850605    0.00000000
 N          0.00000000    1.40923610    0.00000000
 H          2.05161515    1.30374010    0.00000000
 H          2.14829915   -1.17581008    0.00000000
 H         -2.05161515    1.30374010    0.00000000
 C         -1.19177008   -0.66851505    0.00000000
 H         -2.14829915   -1.17581008    0.00000000
 C          0.00000000   -1.37668510    0.00000000
 H          0.00000000   -2.46028118    0.00000000

pyridine_s0.wfx
```

B) Generate integration files using AIMAll, by launching AIMQB in command line mode:

```
> aimqb.ish -nogui pyridine_s0.wfx
```

C) Input .bad file for ESI calculation:

```
$TITLE
Molecule pyridine_s0 Restricted wavefcn
$READWFN
pyridine_s0.wfx
$MCI
$RINGS
1 ring(s) found
6 membered ring
1 2 4 3 8 10
11 atoms
```

D) Run the ESI program in the folder with the .wfx and integration files:

```
> ESI pyridine_s0.bad > pyridine_s0.bad.out
```

Table S64. Example describing the procedure of MCI calculations for **1** in the T_1 state.

A) Gaussian input file for producing the .wfx file needed as input for MCI calculations.

```
#p cam-b3lyp/6-311+g(d,p)  6d 10f out=wfx scf=qc

To get wfx file

0 3
 C        1.19177008   -0.66851505    0.00000000
 C        1.13720008    0.71850605    0.00000000
 C       -1.13720008    0.71850605    0.00000000
 N        0.00000000    1.40923610    0.00000000
 H        2.05161515    1.30374010    0.00000000
 H        2.14829915   -1.17581008    0.00000000
 H       -2.05161515    1.30374010    0.00000000
 C       -1.19177008   -0.66851505    0.00000000
 H       -2.14829915   -1.17581008    0.00000000
 C        0.00000000   -1.37668510    0.00000000
 H        0.00000000   -2.46028118    0.00000000

pyridine_t1.wfx
```

B) Generate integration files using AIMAll, by launching AIMQB in command line mode:

```
> aimqb.ish -nogui pyridine_t1.wfx
```

C) Input .bad file for ESI calculation:

```
$TITLE
Molecule pyridine_s0 Unrestricted wavefcn
$READWFN
pyridine_s0.wfx
$MCI
$RINGS
1 ring(s) found
6 membered ring
1 2 4 3 8 10
11 atoms
$SPINAROMA
```

D) Run the ESI program in the folder with the .wfx and integration files:

```
> ESI pyridine_t1.bad > pyridine_t1.bad.out
```

Table S65. Example describing the procedure of EDDB calculations for **1** in the S_0 state.

A) Gaussian input file running NBO for producing the .fchk and .49 files needed as input for the EDDB script.

```
%chk=pyridine_s0.chk
#p CAM-B3LYP/6-311+g(d,p) gfinput density=current pop=nboread

To get fchk and 49 files

0 1
 C      1.19177008   -0.66851505    0.00000000
 C      1.13720008    0.71850605    0.00000000
 C     -1.13720008    0.71850605    0.00000000
 N      0.00000000    1.40923610    0.00000000
 H      2.05161515    1.30374010    0.00000000
 H      2.14829915   -1.17581008    0.00000000
 H     -2.05161515    1.30374010    0.00000000
 C     -1.19177008   -0.66851505    0.00000000
 H     -2.14829915   -1.17581008    0.00000000
 C      0.00000000   -1.37668510    0.00000000
 H      0.00000000   -2.46028118    0.00000000

$NBO SKIPBO FILE=pyridine_s0 DMNAO=W49 AONAO=W49 $END
```

B) Run the program with command line arguments. For $EDDB_H$:

```
> RunEDDB -q -i pyridine_s0.fchk pyridine_s0.49 -h -o EDDB_h.fchk > EDDB_h.out
```

Table S66. Examples describing the procedure of MICD calculations for **1** in the S_0 state.

A) Gaussian input file for producing the .wfx file needed as input for MICD calculations.

```
#p cam-b3lyp/6-311+g(d,p)  6d 10f nmr=csgt output=(wfx,csgtcx) scf=qc nosymm

To get NMR wfx files

0 1
 C      1.19177008   -0.66851505    0.00000000
 C      1.13720008    0.71850605    0.00000000
 C     -1.13720008    0.71850605    0.00000000
 N      0.00000000    1.40923610    0.00000000
 H      2.05161515    1.30374010    0.00000000
 H      2.14829915   -1.17581008    0.00000000
 H     -2.05161515    1.30374010    0.00000000
 C     -1.19177008   -0.66851505    0.00000000
 H     -2.14829915   -1.17581008    0.00000000
 C      0.00000000   -1.37668510    0.00000000
 H      0.00000000   -2.46028118    0.00000000

pyridine_s0.wfx
```

B) MICD input file for calculation of π-electron bond current strengths.

```
# MICD INPUT
**WFX NAME
pyridine_s0
**OUT NAME
pyridine_s0
**WAVE FUNCTION
restricted
**PRINT_MATRICES
*one_e_matrices
0
*pertubed_orbitals
0
*pertubed_densities
0
**MAGNETIC FIELD
0.000 0.000 1.000
**DECOMPOSITION
MO
*mol_orbitals
3
16
19
20
**RUN TYPE
integrate_cd
*integrate_type
from_ring_center
*number of bonds
6
*bond list
 1
 3 4
 1 0
 2
 8 3
 1 0
 3
10 8
 1 0
 4
 1 10
 1 0
 5
 2 1
 1 0
 6
 4 2
 1 0
*rings_data
1
  4  2  1 10  8  3  4
```

C) MICD input file for calculations of π-electron MICD in the plane 1 bohr above the molecular plane. These data are written in a .vtk file which can be visualized by the ParaView program.

```
# MICD INPUT
**WFX NAME
pyridine_s0
**OUT NAME
pyridine_s0
**WAVE FUNCTION
restricted
**PRINT_MATRICES
*one_e_matrices
0
*pertubed_orbitals
0
*pertubed_densities
0
**MAGNETIC FIELD
0.000 0.000 1.000
**DECOMPOSITION
MO
*mol_orbitals
3
16
19
20
**RUN TYPE
vtk_out
*file name
pyridine_s0_pi
*vtk_options
plot
*normal length
1.0
*plot origine
0.0000 0.0000 1.0000
*x_steps
2.0000 0.0000 1.0000
*y_steps
0.0000 2.0000 1.0000
*x_min_max
-20 20
*y_min_max
-20 20
*planar_opt
0
# END OF MICD INPUT
```

<table>
<tr><td>*xy_z_num_steps
100 100
END OF MICD INPUT</td><td></td></tr>
</table>

Table S67. Examples describing the procedure of MICD calculations for **1** in the T_1 state.

<table>
<tr><td colspan="3">A) Gaussian input file for producing the .wfx file needed as input for MICD calculations.

#p ucam-b3lyp/6-311+g(d,p) 6d 10f nmr=csgt output=(wfx,csgtcx) scf=qc nosymm

To get NMR wfx files

0 1
C 1.19177008 -0.66851505 0.00000000
C 1.13720008 0.71850605 0.00000000
C -1.13720008 0.71850605 0.00000000
N 0.00000000 1.40923610 0.00000000
H 2.05161515 1.30374010 0.00000000
H 2.14829915 -1.17581008 0.00000000
H -2.05161515 1.30374010 0.00000000
C -1.19177008 -0.66851505 0.00000000
H -2.14829915 -1.17581008 0.00000000
C 0.00000000 -1.37668510 0.00000000
H 0.00000000 -2.46028118 0.00000000

pyridine_t1.wfx</td></tr>
<tr><td>B) MICD input file for calculation of α-π-electron bond current strengths.

MICD INPUT
**WFX NAME
pyridine_t1-a
**OUT NAME
pyridine_t1-a
**WAVE FUNCTION
unrestricted
**PRINT_MATRICES
*one_e_matrices
0
*pertubed_orbitals
0
*pertubed_densities
0
**MAGNETIC FIELD
0.000 0.000 1.000
**DECOMPOSITION
MO
*mol_orbitals
4</td><td>C) MICD input file for calculation of β-π-electron bond current strengths.

MICD INPUT
**WFX NAME
pyridine_t1-b
**OUT NAME
pyridine_t1-b
**WAVE FUNCTION
unrestricted
**PRINT_MATRICES
*one_e_matrices
0
*pertubed_orbitals
0
*pertubed_densities
0
**MAGNETIC FIELD
0.000 0.000 1.000
**DECOMPOSITION
MO
*mol_orbitals
3</td><td>D) MICD input file for calculation of π-electron MICD in the plane 1 bohr above the molecular plane. These data are written in a .vtk file which can be visualized by the ParaView program.

MICD INPUT
**WFX NAME
pyridine_t1
**OUT NAME
pyridine_t1
**WAVE FUNCTION
unrestricted
**PRINT_MATRICES
*one_e_matrices
0
*pertubed_orbitals</td></tr>
</table>

15<br>20<br>21<br>22<br>**RUN TYPE<br>integrate_cd<br>*integrate_type<br>from_ring_center<br>*number of bonds<br>6<br>*bond list<br>1<br>3 4<br>1 0<br>2<br>8 3<br>1 0<br>3<br>10 8<br>1 0<br>4<br>1 10<br>1 0<br>5<br>2 1<br>1 0<br>6<br>4 2<br>1 0<br>*rings_data<br>1<br>4 2 1 10 8 3 4<br>*xy_z_num_steps<br>100 100<br># END OF MICD INPUT	38<br>41<br>42<br>**RUN TYPE<br>integrate_cd<br>*integrate_type<br>from_ring_center<br>*number of bonds<br>6<br>*bond list<br>1<br>3 4<br>1 0<br>2<br>8 3<br>1 0<br>3<br>10 8<br>1 0<br>4<br>1 10<br>1 0<br>5<br>2 1<br>1 0<br>6<br>4 2<br>1 0<br>*rings_data<br>1<br>4 2 1 10 8 3 4<br>*xy_z_num_steps<br>100 100<br># END OF MICD INPUT	0<br>*pertubed_densities<br>0<br>**MAGNETIC FIELD<br>0.000 0.000 1.000<br>**DECOMPOSITION<br>MO<br>*mol_orbitals<br>7<br>15<br>20<br>21<br>22<br>38<br>41<br>42<br>**RUN TYPE<br>vtk_out<br>*file name<br>pyridine_t1_pi<br>*vtk_options<br>plot<br>*normal length<br>1.0<br>*plot origine<br>0.0000 0.0000 1.0000<br>*x_steps<br>2.0000 0.0000 1.0000<br>*y_steps<br>0.0000 2.0000 1.0000<br>*x_min_max<br>-20 20<br>*y_min_max<br>-20 20<br>*planar_opt<br>0<br># END OF MICD INPUT

6. Cartesian Coordinates

A data set collection of the calculations is available through ioChem-BD repository [55] at http://dx.doi.org/10.19061/iochem-bd-4-51.

References

[1] M. J. Frisch, G. W. Trucks, H. B. Schlegel, G. E. Scuseria, M. A. Robb, J. R. Cheeseman, G. Scalmani, V. Barone, G. A. Petersson, H. Nakatsuji, X. Li, M. Caricato, A. V. Marenich, J. Bloino, B. G. Janesko, R. Gomperts, B. Mennucci, H. P. Hratchian, J. V. Ortiz, A. F. Izmaylov, J. L. Sonnenberg, F. Ding Williams, F. Lipparini, F. Egidi, J. Goings, B. Peng, A. Petrone, T. Henderson, D. Ranasinghe, V. G. Zakrzewski, J. Gao, N. Rega, G. Zheng, W. Liang, M. Hada, M. Ehara, K. Toyota, R. Fukuda, J. Hasegawa, M. Ishida, T. Nakajima, Y. Honda, O. Kitao, H. Nakai, T. Vreven, K. Throssell, J. A. Montgomery Jr., J. E. Peralta, F. Ogliaro, M. J. Bearpark, J. J. Heyd, E. N. Brothers, K. N. Kudin, V. N. Staroverov, T. A. Keith, R. Kobayashi, J. Normand, K. Raghavachari, A. P. Rendell, J. C. Burant, S. S. Iyengar, J. Tomasi, M. Cossi, J. M. Millam, M. Klene, C. Adamo, R. Cammi, J. W. Ochterski, R. L. Martin, K. Morokuma, O. Farkas, J. B. Foresman, D. J. Fox, Gaussian 16, Revision B.01, Gaussian, Inc., Wallingford, CT, **2016**.

[2] Yanai, T.; Tew, D. P.; Handy, N. C., *Chem. Phys. Lett.* **2004**, 393, 51–57.

[3] Becke, A. D., *J. Chem. Phys.* **1993**, 98, 5648–5652.

[4] Stephens, P. J.; Devlin, F. J.; Chabalowski, C. F.; Frisch, M. J., *J. Phys. Chem.* **1994**, 98, 11623–11627.

[5] Becke, A. D., *Phys. Rev. A* **1988**, 38, 3098–3100.

[6] Cizek, J., *Adv. Chem. Phys.* **1969**, 14, 35–89.

[7] Krishnan, R.; Binkley, J. S.; Seeger, R.; Pople, J. A., *J. Chem. Phys.* **1980**, 72, 650–654.

[8] Clark, T.; Chandrasekhar, J.; Spitznagel, G. W.; Schleyer, P. v. R., *J. Comput. Chem.* **1983**, 4, 294–301.

[9] Wadt, W. R.; Hay, P. J., *J. Chem. Phys.* **1985**, 82, 270–283.

[10] Wadt, W. R.; Hay, P. J., *J. Chem. Phys.* **1985**, 82, 284–298.

[11] Wadt, W. R.; Hay, P. J., *J. Chem. Phys.* **1985**, 82, 299–310.

[12] Dykstra, C. E., *Chem. Phys. Lett.* **1977**, 45, 466–469.

[13] Handy, N. C.; Pople, J. A.; Head-Gordon, M.; Raghavachari, K.; Trucks, G. W., *Chem. Phys. Lett.* **1989**, 164, 185–192.

[14] Gunnarsson, O.; Lundqvist, B. I., *Phys. Rev. B* **1976**, 13, 4274–4298.

[15] Glendening, E. D.; Reed, A. E.; Carpenter, J. E.; Weinhold, F., NBO 3.1, Theoretical Chemistry Institute, University of Wisconsin, Madison, **1990**.

[16] Giambiagi, M.; Segre de Giambiagi, M.; dos Santos Silva, C. D.; Paiva de Figueiredo, A.; *Phys. Chem.Chem. Phys.* **2000**, 2, 3381–3392.

[17] Bultinck, P.; Ponec, R.; Van Damme, S., *J. Phys. Org. Chem.* **2005**, 18, 706–718.

[18] Keith, T. A., AIMAll (Version 19.10.12), TK Gristmill Software, Overland Park KS, USA **2014**. http://aim.tkgristmill.com

[19] Matito, E., ESI-3D: Electron sharing indexes program for 3D molecular space partitioning. http://iqc.udg.es/~eduard/ESI, IQCC (Girona, Catalonia) and DIPC (Donostia, Euskadi), Spain **2015**.

[20] Matito, E.; Solà, M.; Salvador, P.; Duran, M., *Faraday Discuss*. **2007**, 135, 325–345.

[21] Poater, J.; Solà, M.; Duran, M.; Fradera, X., *Theor. Chem. Acc.* **2002**, 107, 362–371.

[22] Feixas, F.; Vandenbussche, J.; Bultinck, P.; Matito, E.; Solà, M., *Phys. Chem. Chem. Phys*. **2011**, 13, 20690−20703.

[23] Szczepanik, D. W.; Andrzejak, M.; Dyduch, K.; Żak, E.; Makowski, M.; Mazur, G.; Mrozek, J. A., *Phys. Chem. Chem. Phys*. **2014**, 16, 20514−20523.

[24] Szczepanik, D. W.; Andrzejak, M.; Dominikowska, J.; Pawełek, B.; Krygowski, T. M.; Szatylowicz, H.; Sola, M., *Phys. Chem. Chem. Phys*. **2017**, 19, 28970−28981.

[25] M. J. Frisch, G. W. Trucks, H. B. Schlegel, G. E. Scuseria, M. A. Robb, J. R. Cheeseman, G. Scalmani, V. Barone, B. Mennucci, G. A. Petersson, H. Nakatsuji, M. Caricato, X. Li, H. P. Hratchian, A. F. Izmaylov, J. Bloino, G. Zheng, J. L. Sonnenberg, M. Hada, M. Ehara, K. Toyota, R. Fukuda, J. Hasegawa, M. Ishida, T. Nakajima, Y. Honda, O. Kitao, H. Nakai, T. Vreven, J. A. Montgomery, Jr., J. E. Peralta, F. Ogliaro, M. Bearpark, J. J. Heyd, E. Brothers, K. N. Kudin, V. N. Staroverov, R. Kobayashi, J. Normand, K. Raghavachari, A. Rendell, J. C. Burant, S. S. Iyengar, J. Tomasi, M. Cossi, N. Rega, J. M. Millam, M. Klene, J. E. Knox, J. B. Cross, V. Bakken, C. Adamo, J. Jaramillo, R. Gomperts, R. E. Stratmann, O. Yazyev, A. J. Austin, R. Cammi, C. Pomelli, J. W. Ochterski, R. L. Martin, K. Morokuma, V. G. Zakrzewski, G. A. Voth, P. Salvador, J. J. Dannenberg, S. Dapprich, A. D. Daniels, O. Farkas, J. B. Foresman, J. V. Ortiz, J. Cioslowski and D. J. Fox, Gaussian 09, Revision D.01, Gaussian, Inc., Wallingford, CT, **2009**.

[26] Glendening, E. D.; Badenhoop, J. K.; Reed, A. E.; Carpenter, J. E.; Bohmann, J. A., Morales, C. M.; Landis, C. R.; Weinhold, F., NBO 6.0, Theoretical Chemistry Institute, University of Wisconsin, Madison, **2013**.

[27] Hanwell, M. D.; Curtis, D. E.; Lonie, D. C.; Vandermeersch, T.; Zurek, E.; Hutchison, G. R., Avogadro: An Open-Source Molecular Builder and Visualization Tool. 1.2.0; Slashdot Media: SourceForge, **2017**.

[28] Schleyer, P. V. R.; Maerker, C.; Dransfeld, A.; Jiao, H.; Van Eikema Hommes, N. J. R.; *J. Am. Chem. Soc.* **1996**, 118, 6317–6318.

[29] Chen, Z.; Wannere, C. S.; Corminboeuf, C.; Puchta, R.; Schleyer, P. v. R.; *Chem. Rev.* **2005**, 105, 3842–3888.

[30] Gershoni-Poranne, R.; Stanger, A.; *Chem. Soc. Rev.* **2015**, 44, 6597–6615.

[31] Lazzeretti, P.; *Prog. Nucl. Magn. Reson. Spectrosc.* **2000**, 36, 1–88.

[32] Mandado, M.; Graña, A. M.; Pérez-Juste, I.; *J. Chem. Phys.* **2008**, 129, 164114.

[33] Keith, T.A.; Bader, R.F.W.; *Chem. Phys. Lett.* **1993**, 210, 223–231.

[34] Keith, T.A.; Bader, R.F.W. *J. Chem. Phys.* **1993**, 99, 3669–3682.

[35] Lazzeretti, P.; Malagoli, M.; Zanasi, R., *Chem. Phys. Lett.* **1994**, 220, 299–304.

[36] Steiner, E.; Fowler, P.W., *J. Phys. Chem. A.* **2001**, 105, 9553–9562.

[37] Jusélius, J.; Sundholm, D.; Gauss, J., *J. Chem. Phys.* **2004**, 121, 3952–3963.

[38] Kruszewski, J.; Krygowski, T. M. M., *Tetrahedron Lett.* **1972**, 13, 3839–3842.

[39] Krygowski, T. M., *J. Chem. Inf. Model.* **1993**, 33, 70–78.

[40] Fdez. Galván, I.; Vacher, M.; Alavi, A.; Angeli, C.; Aquilante, F.; Autschbach, J.; Bao, J. J.; Bokarev, S. I.; Bogdanov, N. A.; Carlson, R. K.; Chibotaru, L. F.; Creutzberg, J.; Dattani, N.; Delcey, M. G.; Dong, S. S.; Dreuw, A.; Freitag, L.; Frutos, L. M.; Gagliardi, L.; Gendron, F.; Giussani, A.; González, L.; Grell, G.; Guo, M.; Hoyer, C. E.; Johansson, M.; Keller, S.; Knecht, S.; Kovačević, G.; Källman, E.; Manni, G. L.; Lundberg, M.; Ma, Y.; Mai, S.; Malhado, J. P.; Malmqvist, P. Å.; Marquetand, P.; Mewes, S. A.; Norell, J.; Olivucci, M.; Oppel, M.; Phung, Q. M.; Pierloot, K.; Plasser, F.; Reiher, M.; Sand, A. M.; Schapiro, I.; Sharma, P.; Stein, C. J.; Sørensen, L. K.; Truhlar, D. G.; Ugandi, M.; Ungur, L.; Valentini, A.; Vancoillie, S.; Veryazov, V.; Weser, O.; Wesołowski, T. A.; Widmark, P.-O.; Wouters, S.; Zech, A.; Zobel, J. P.; Lindh, R., *J. Chem. Theory Comput.* **2019**, 15, 11, 5925–5964.

[41] Kwan, E. E.; Liu, R. Y.; *J. Chem. Theory Comput.* **2015**, 11, 5083–5089.

[42] Mai, S.; Marquetand, P.; González L.; *WIREs Comput. Mol. Sci.* **2018**, 8, e1370.

[43] Barbatti, M.; Sen, K.; *Int. J. Quant. Chem.* **2016**, 116, 762–771.

[44] Cioslowski, J.; Matito, E.; Solà, M., *J. Phys. Chem. A* **2007**, 111, 6521–6525.

[45] Matito, E., *Phys. Chem. Chem. Phys.* **2016**, 18, 11839–11846.

[46] Lee, T. J.; Taylor, P. R.; *Int. J. Quant. Chem.* **1989**, 36, 199–207.

[47] Jayatilaka, D.; Lee, T. J.; *J. Chem. Phys.* **1993**, 98, 9734–9747.

[48] Rienstra-Kiracofe, J. C.; Allen, W. D.; Schaefer III, H. F.; *J. Phys. Chem. A* **2000**, 44, 9823–9840.

[49] Cai, Z.-L.; Reimers, J. R., *J. Phys. Chem. A* **2000**, 104, 36, 8389–8408.

[50] Albright, T. A.; Burdett, J. K.; Whangbo, M.-H.; *Orbital Interactions in Chemistry*, John Wiley & Sons, Inc., Hoboken, New Jersey, Second Edition, **2013**.

[51] Bai, Y.; Deng, J.; Xie, W.; Xiao, J.; Zhang, J.; Wang, Y.; Guo, X.; Wang, H., *J. Phys. Chem. A*, **2023**, 127, 9273–9282.

[52] Chen, D.; Shen, T.; An, K.; Zhu, J., *Commun. Chem.* **2018**, 1, 18.

[53] Shen T.; Chen D.; Lin L.; Zhu J., *J. Am. Chem. Soc.* **2019**, 141, 5720−5727.

[54] Havenith, R. W. A.; Jenneskens, L. W.; Fowler, P. W.; *Chem. Phys. Lett.*, **2003**, 367, 468–474.

[55] Álvarez-Moreno, M.; de Graaf, C.; López, N.; Maseras, F.; Poblet, J. M.; Bo, C., *J. Chem. Inf. Model.* **2015**, 55, 1, 95–103.
